# Effect of Probiotics on Psychiatric Symptoms and Central Nervous System Functions in Human Health and Disease: A Systematic Review and Meta-Analysis

**DOI:** 10.3390/nu14030621

**Published:** 2022-01-30

**Authors:** Charlotte Le Morvan de Sequeira, Charlotte Hengstberger, Paul Enck, Isabelle Mack

**Affiliations:** Department of Psychosomatic Medicine and Psychotherapy, University Medical Hospital, 72076 Tübingen, Germany; charlotte.le-morvan-de-sequeira@student.uni-tuebingen.de (C.L.M.d.S.); charlotte.hengstberger@student.uni-tuebingen.de (C.H.); paul.enck@uni-tuebingen.de (P.E.)

**Keywords:** probiotics, paraprobiotics, central nervous system (CNS), anxiety, depression, stress, mood, psychiatric distress, systematic review, meta-analysis

## Abstract

Background: The gut microbiota impacts on central nervous system (CNS) function via the microbiota–gut–brain axis. Thus, therapeutics targeting the gut microbiota such as probiotics have the potential for improving mental health. This meta-analysis synthesizes the evidence regarding the impacts of probiotics on psychological well-being, psychiatric symptoms and CNS functioning. Methods: The Preferred Reporting Items for Systematic Reviews and Meta-Analyses guidelines were applied for executing this review using the databases PubMed, Web of Science and Cochrane Library. The data were summarized at qualitative and quantitative level. Results: Fifty-four randomized placebo-controlled studies were included, of which 30 were eligible for meta-analysis. If investigated, the probiotics mostly exerted effects on CNS function. Most probiotics did not affect mood, stress, anxiety, depression and psychiatric distress when compared to placebo at the qualitative level. At quantitative level, depression and psychiatric distress improved slightly in the probiotic condition (depression: mean difference −0.37 (95% CI: −0.55, −0.20); *p* ≤ 0.0001; psychiatric distress: mean difference −0.33 (95% CI: −0.53, −0.13); *p* = 0.001). Conclusions: To date it is unclear to which extent and in which specific areas next generation probiotics selected and developed for their ability to improve psychiatric condition and potentially other CNS functions are promising.

## 1. Introduction

Mental health disorders such as anxiety and depression are highly prevalent and are steadily increasing. Their impact on quality of life of the patients and their families and their economic burden for society is enormous [1]. In addition, anxiety and depression are often comorbidities of other mental and somatic disorders, especially in chronic conditions [2]. Since the gastrointestinal (GI) microbiota influences brain function and behavior, understanding the mechanisms could provide resources for improving psychological well-being in health and maybe even in psychiatric conditions [3].

The GI microbiota is the total amount of living microorganisms colonizing the GI tract of a host organism [4]. The gut is exposed to numerous potential pathogens. Thus, it is necessary for the host to prevent their uncontrolled penetration into the body. Immune defense via unspecific mechanisms along with the gut-associated lymphoid tissue are essential in this interplay. Furthermore, the immune-modulating potential of the GI microbiota [5] and some probiotics has been reported [6]. However, the interactions between the indigenous microorganisms and the host are mostly beneficial. The former are important for the maintenance of the gut barrier function but also the overall health of the host [7,8]. Interestingly, the composition of the gut microbiota is different in patients with severe and moderate major depression disorder in comparison to healthy individuals [5]. In line with this, the gut microbiota can exert effects on central nervous system (CNS) functions via the microbiota–gut–brain axis, which has been extensively reviewed elsewhere [9,10,11].

Thus, it is logical, why therapeutics targeting the GI microbiota, such as prebiotics, probiotics and postbiotics/paraprobiotics (inactivated bacteria or fractions) [12,13] and fermented milks [14], are of potential interest for (i) influencing mood and stress resilience in health and disease and, (ii) adjunct treatment of psychiatric and functional CNS disorders [9]. In pre-clinical studies with rodents, the evidence for probiotics being beneficial for memory performance, improving stress response, and even reducing anxiety and depression, is convincing. However, not all pre-clinical studies report superiority of probiotics over placebo, the literature being reviewed elsewhere [9,15,16]. Besides probiotics, there are also other potential GI microbiota-modulating substances [17].

In humans, the situation is far less clear, not only because studies in rodents outnumber human studies, but also because a number of experiments can only be conducted in pre-clinical models due to ethical reasons and translation into human models is extremely challenging and not always possible. Beyond this, the lack in the field becomes obvious when considering that only six randomized controlled trials (RCT) [18,19,20,21,22,23] exist investigating the effects of probiotics on CNS function by brain imaging studies. Nevertheless, the field has been growing over the last decade and the questions arise whether or not probiotics and postbiotics are able to influence psychological well-being, psychiatric symptoms and central functions and, if yes, to which extent effects can be expected in humans. 

Thus, the aim of this systematic review and meta-analysis was to synthesize the evidence regarding the effects of probiotics and postbiotics on (i) psychological well-being (mood and stress response), (ii) psychiatric symptoms (depression, anxiety, psychiatric distress) and (iii) central functioning. The latter is defined as neural brain activity measured by electroencephalography (EEG) and by imaging methods in both healthy participants and patients with functional CNS or psychiatric disorders. 

## 2. Materials and Methods

### 2.1. Literature Information Sources and Search Strategy

The Preferred Reporting Items for Systematic Reviews and Meta-Analyses (PRISMA) guidelines were applied for executing this review [24]. The 3 databases Web of Science, PubMed, and Cochrane Library were searched on 20 March 2021 and updated on 24 September 2021. The strategy of the full search is reported in the Supporting Information Text S1, consisting of the two modules, i.e., probiotics and CNS function. This review is registered on the PROSPERO platform (CRD42021253080).

### 2.2. Eligibility Criteria

The five PICOS dimensions, i.e., participants (P), interventions (I), comparators (C), outcome (O) and study design (S) [25] were applied to define the eligibility criteria. 

Participants: Participants were adults of all ages and both sexes, either healthy or with a specified functional CNS or psychiatric disorder: attention-deficit hyperactivity disorders (ADHD), autism spectrum disorders (ASD), bipolar disorders (BD), depression, tension headache (TD), neurotic, stress-associated and somatoform disorders and affective disorders. Structural and neurodegenerative disorders, including multiple sclerosis, Alzheimer’s, Huntington’s, ALS, and Parkinson’s, conditions secondary to infections, conditions with proven (or suspected) genetic or other somatic origin and conditions with hormonal origins like pregnancy were excluded. This also excludes CNS diseases with a traumatic, vascular, metabolic, autoimmune and neoplastic origin. Somatoform disorders were excluded unless a psychologic component is a prerequisite for diagnosis; this restricts fibromyalgia syndrome (FMS) and chronic fatigue syndrome (CFS) to the subgroup with proven psychiatric comorbidity. Functional bowel disorders were excluded and reviewed elsewhere [26].

Interventions: Trials assessing the effectivity of viable and non-viable microorganisms or microbial cell extracts to be used as probiotics, paraprobiotics, bacterial lysate, psychobiotics, single- and/or multi-strain preparations. The minimum of treatment duration had to be 3 weeks. Exclusion criteria were studies investigating prebiotics, synbiotics or antibiotics.

Comparators: A blinded placebo control group was necessary for inclusion.

Outcome Measures: Behavioral measures, neuropsychological measures (psychometric tests) and neurophysiological measures (e.g., electroencephalography, magnetoencephalography and functional magnetic resonance imaging). In the qualitative analysis, studies that only involved neuroendocrine, neurochemical and neuroimmunologic measures (laboratory) were not included and only mentioned in the discussion (e.g., measuring neurochemical level, HPA axis activity). The reason is the heterogeneity of studies tested and their methodological differences. 

Study design: Randomized, double-blinded, placebo-controlled trials. 

### 2.3. Study Selection, Data Collection and Organization

First, the results of the database searches were combined. Next, the duplicates were removed followed by screening the titles and abstracts. Finally, the evaluation of the full-text articles for eligibility was performed (CLMdS and IM). Uncertainties were discussed between the authors (CLMdS, IM) (<5%). Discrepancies were solved by including a third person (PE). For classification, the studies were assigned to one of six groups.

1. Group 1—Probiotics and Depression
2. Group 2—Probiotics and Anxiety
3. Group 3—Probiotics and Stress
4. Group 4—Probiotics and Cognitive Function
5. Group 5—Probiotics and Mental Health and Mood
6. Group 6—Probiotics and other CNS states

### 2.4. Data Items and Statistics

For study characterization, the information on year of publication, country of origin, study type, probiotic intervention, method for data collection, study outcomes including behavioral or neuropsychological validated questionnaires, sample characteristics (sample size, age, sex) was extracted from each included study. Across the studies, the characteristics are reported as frequency and per cent (%) or median (interquartile range), minimum and maximum for sample size, intake time, sex and age.

The evaluation of data was performed qualitatively and also quantitatively (meta-analysis) where possible.

The qualitative analyses synthesized the findings for their direction of change between the groups. This was necessary, because (i) some studies reported insufficient data (e.g., studies only mentioned that no group differences existed without reporting the data) and/or (ii) the heterogeneity of the applied measurements was too high for a quantitative summary [27,28]. 

A random-effect model for parallel-group designed studies was applied for meta-analysis, using the software package Review Manager, version 5.4 [29]. The data of the questionnaires are presented as mean and SD separately for the control and intervention groups. The difference is expressed as standardized mean difference and 95% confidence interval and visualized as forest plots. Statistical heterogeneity was explored by visual inspection of forest plots and using the I^2^ statistics to quantify inconsistency between the studies. Subgroup analyses were conducted for intake period (4–8 weeks versus 9–24 weeks intake time), the type of probiotic preparation (single versus multi-strain probiotics), the location of the study (Asia vs Not Asia), the application form (liquid/powder versus capsule/pills) and the health state (healthy vs diagnosed psychiatric disorder) for reducing heterogeneity and/or to advance the understanding of the findings. Participants were considered as healthy if they had not been diagnosed with a psychiatric disorder according to validated guidelines such as the Diagnostic and Statistical Manual of Mental Disorders (DSM). 

Several studies used more than one questionnaire to assess the same outcome. For example, to assess the symptoms of anxiety, the State Trait Anxiety Inventory (STAI), the Beck Anxiety Inventory (BAI) and Hamilton Anxiety Rating Scale (HAM-A) were used in parallel in one study [30]. To compare the data across different questionnaires, in case of multiple questionnaires, for anxiety the STAI state was used and for depression the Beck Depression Inventory (BDI). The reason is that these were the most utilized questionnaires for the respective outcomes. However, in addition, all data are also reported questionnaire-wise (in the Supplementary Materials) but without total summary for methodological correctness.

Missing data were requested from authors and 46.15% (6 out of 13) responded to the inquiry.

### 2.5. Risk of Bias

The risk of bias was assessed using the Cochrane risk-of-bias tool for randomized trials (RoB 2) [31]. It is structured into five domains addressing different types of bias: randomization process, deviations from the intended interventions, missing outcome data, measurement of the outcome and selection of the reported result. First, specific questions in each domain need to be answered for each single study. Next, the RoB2 algorithm calculates the risks of the individual domains. Finally, an overall risk is computed and expressed as “low risk of bias”, “risk with some concerns” or “high risk of bias”.

## 3. Results

**Figure 1:**
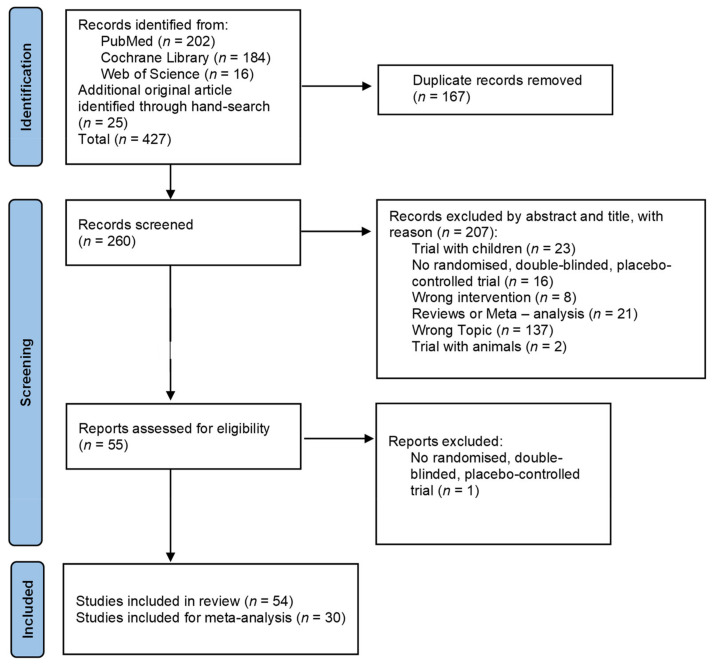
PRISMA flow chart for study inclusion.

The literature search process is presented in Figure 1. The search identified 427 studies of which 54 remained for analysis.

### 3.1. Study Selection and Categorization

The characteristics of the single trials are reported in Table 1 along with the detailed information on bacterial species of the probiotics provide in Table S1 and an overview of the applied questionnaires and tests and their abbreviations in Table S2. The characteristics across the studies are given below and in Table S3.

### 3.2. Summary of Study Characteristics

The publishing dates of the studies were between 2007 and 2021. Most studies were conducted in Asia (*n* = 28; 51.9%) [30,32,33,36,41,43,44,45,47,48,50,51,53,54,55,56,57,58,62,63,69,70,72,74,75,77,78,79], followed by Europe (*n* = 18; 33.3%) [18,19,20,22,34,35,37,40,42,46,49,52,59,60,64,65,67,73], America (*n* = 7; 12%) [21,38,39,61,68,71,76] and Oceania (*n* = 1; 1.9%) [66]. In total, the 54 trials included 4449 randomized participants; 3700 participants finished trials and 4130 participants were analyzed. The median age was 35.6 (24.9–50.1) years and 60.1% were women. The intervention durations ranged between 3 and 24 weeks with a median length of 8 (4–12) weeks. Most probiotics were ingested as powders (*n* = 19; 35.2%) followed by liquids such as milk (*n* = 12; 22.2%), capsules (*n* = 13; 24.1%) and applications with tablets or pills (*n* = 7; 13%). Three studies did not report the application form (5.6%).

The intake ranged from one to four applications of probiotics per day. Mostly, probiotic intake was once per day (*n* = 46; 85.2%). The studies investigated single-strain (*n* = 29; 53.7%) and multi-strain probiotics (*n* = 25; 46.3%). The latter consisted of two to twenty different probiotic strains. The median number of colony-forming units (CFUs) was 1 × 10^10^ (2.5 × 10^9^–2.1 × 10^10^) CFUs per day. The range was between 2.3 × 10^7^ and 1.4 × 10^11^ CFUs per day. Five studies did not report the CFU (9.3%)

Most trials compared a probiotic with a placebo group. Twelve (22.2%) studies investigated more than two groups. Four studies compared the placebo group with several probiotic groups with different CFU concentration [36,54,72,76], while two studies compared them with different species [46,48]. The study with the most probiotic groups investigated five different groups [76]. Three studies compared the results of the probiotic groups with the results of a placebo group and also with the results of a no interventional group [18,19,21]. Five studies were conducted as cross-over design and all other studies as parallel group design [34,37,49,70,78].

Several studies added to their probiotic treatment other substances such as vitamin D (*n* = 3; 5.6%) [41,62,64], selective serotonin reuptake inhibitors (SSRI) (*n* = 2; 3.7%) [30,67], selenium [63] or α-lactalbumin [69]. While Benton et al. (2007) never mentioned any division of the participants in groups [35], another study, Mohammadi et al. (2016), used combinations with capsules and yoghurts with and without probiotics [53].

Only four studies examined the impact on CNS function by using functional magnetic resonance imaging [18,19,20,21], while only six studies used electroencephalography (EEG) [33,34,43,49,56,75]. There was also one study applying magnetoencephalography (MEG) [22], another using actigraphic measurements [78] and one study with electrodermal responses (EDR) [33]. The other studies investigated anxiety, depression, stress, psychiatric distress and other mental or behavioral states by validated questionnaires. An overview of the results of the most frequently used questionnaires are presented study- and questionnaire-wise in Table S4.

Overall, 41 studies presented data of healthy participants with no diagnosed psychiatric disorder (75.9%) and 13 studies (24.1%) of participants with diagnosed psychiatric disorders according to DSM, such as major depressive disorder (*n* = 2; 3.7%) [32,67], depression (*n* = 2; 3.7%) [48,79] and schizophrenia (*n* = 3; 5.6%) [38,41,71]. In addition, studies also included participants with mild cognitive impairment [44], bipolar I/schizoaffective disorder in a manic episode [39], general anxiety disorder [30], chronic fatigue syndrome [61], chronic primary insomnia [43] and fibromyalgia [65]. In addition, five studies only included participants when they achieved a pathological score in questionnaires (9.3%).

Tak Gen Zist Pharmaceutical Company (Tehran, Iran) provided or sponsored six trials from the sample (11.1%), as well as Yakult (Minato, Japan) (*n* = 6; 11.5%), Lallemand Institute (Montréal, QC, Canada) (*n* = 6; 11.1%), Winclove Probiotics (Amsterdam, The Netherlands) (*n* = 5; 9.3%), Asahi Group Holdings (Sumida, Japan) (*n* = 4; 7.7%), Alimentary Health (Cork, Ireland) (*n* = 3; 5.6%), Morinaga Milk Industry (Minato-ku, Japan) (*n* = 3; 5.6%), Pfizer (New York, NY, USA) (*n* = 2; 3.7%) and HOMEOSYN (Rome, Italy) (*n* = 2; 3.7%). From 54 studies in this sample, 37 studies were sponsored or provided by these few manufacturers (68.5%). Among the 15 studies from Japan, 10 (66.7%) were provided from Yakult (*n* = 3; 20%), Asahi Group Holdings (*n* = 4; 26.7%) and Morinaga Milk Industry (*n* = 3; 20%). In Iran, six out of seven studies (85.7%) were provided by Tak Gen Zist Pharmaceutical Company.

For the meta-analysis, 3017 participants out of 30 trials were eligible. There were 2563 participants at the end of the trials and the data of 2595 participants were analyzed. The median age was 36.2 (22.8–51.4) years and 62.9% were women. The intervention periods ranged between 4 and 24 weeks. The median length was 8 (4.3–12) weeks. Most probiotics were ingested as powder (*n* = 8; 26.7%) and were either single-strains (*n* = 16; 53.3%) or multi-strains (*n* = 14; 46.7%). The latter consisted of two to twenty different probiotic strains. The intake ranged from one to four servings per day. Mostly, the intake was once per day (*n* = 24; 80%). The median number of colony-forming units (CFU) was 9 × 10^9^ (3.5 × 10^9^–2 × 10^10^) CFU per day with a range from 2.4 × 10^7^ to 1.4 × 10^11^ CFU per day. An overview of characteristics is provided Table S3.

### 3.3. Summary of Study Outcomes

**Figure 2:**
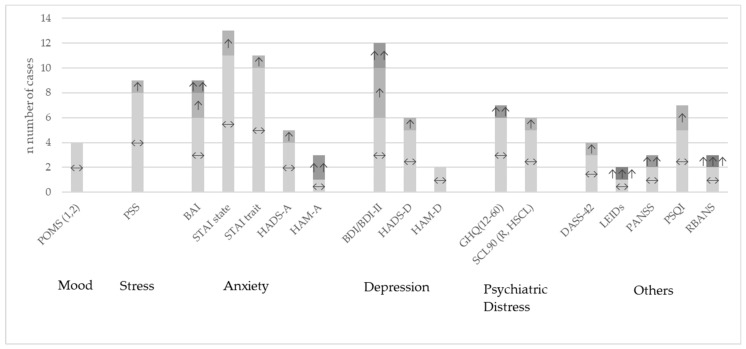
Study outcomes at qualitative level for the most used questionnaires compared between probiotic versus placebo intervention. ↔: no significant differences between the groups; ↑: improvement *p* < 0.05; ↑↑: improvement *p* < 0.01; ↑↑↑: improvement *p* < 0.001; BAI: Beck Anxiety Inventory; BDI: Beck Depression Inventory; DASS-42: Depression, Anxiety and Stress Scale; GHQ: General Health Questionnaire; HADS-A/-D: Hospital Anxiety and Depression Scale; HAM-A: Hamilton Anxiety Rating Scale; HAM-D: Hamilton Depression Rating Scale; LEIDs: Leiden index of depression severity; POMS: Profile of Mood States; PSQI: Pittsburgh Sleep Quality Index; PSS: Perceived Stress Scale; RBANS: Repeatable Battery for the Assessment of Neuropsychological Status; SCL90: Symptoms Checklist-90; STAI: State Trait Anxiety Inventory.

Overall, the heterogeneity of studies was high. Many different questionnaires were used to describe different outcomes with regard to behavioral or neuropsychological aspects. Table S4 shows the most common questionnaires in the review sample and Table S5 specifically for the meta-analytical sample with their results of the comparison between probiotic and placebo group for every single study. An overview across studies at the qualitative level is presented in Figure 2. One study did not report their results differentiated by groups [79] and among the others which reported their results, most studies found no significant differences between the probiotic treatment and the placebo groups, often only stating this finding and not reporting the figures.

**Figure 3:**
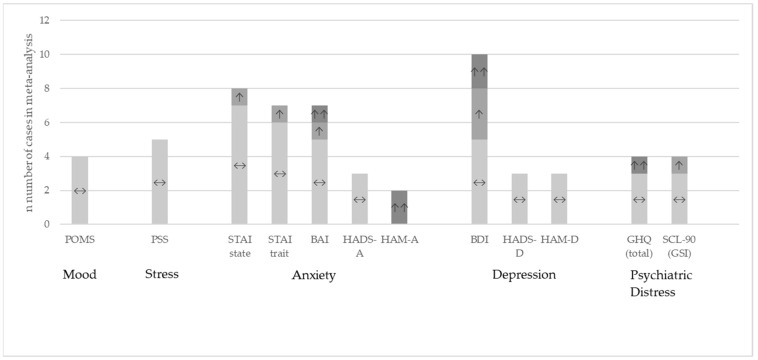
Study outcomes at qualitative level for the studies included in the meta-analysis. Comparison between the probiotic and placebo group [20,30,32,33,37,38,41,42,44,45,46,47,48,49,51,52,55,57,58,63,64,66,67,68,69,70,71,72,77,78]. ↔: no significant differences between the groups; ↑: significant improvement in the probiotic group versus placebo *p* < 0.05; ↑↑: significant improvement in the probiotic group versus placebo *p* < 0.01.

Figure 3 shows the results at qualitative level for the questionnaires eligible for meta-analysis where the data were available from the publication or provided by the authors upon request.

#### 3.3.1. Mood States and Stress

**Figure 4:**
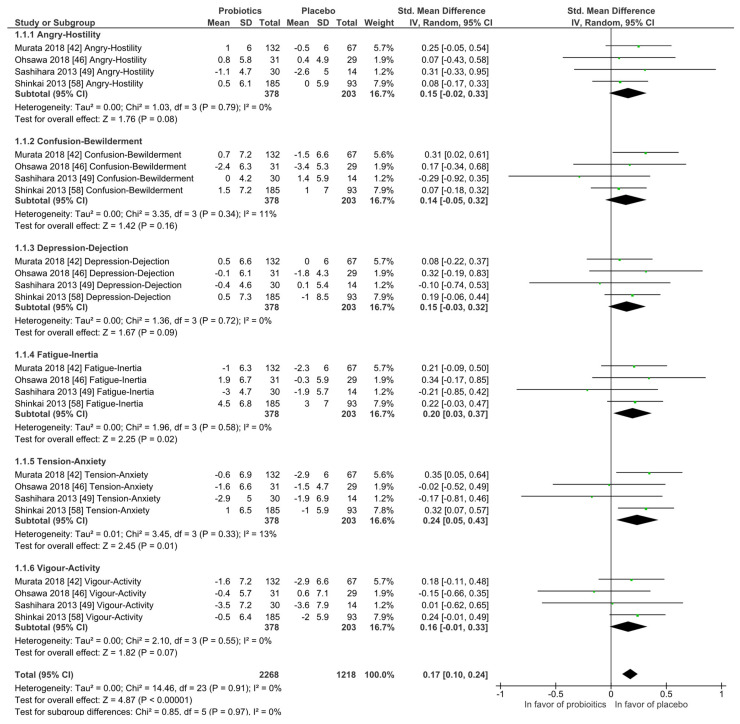
Quantitative analysis for Profile of Mood States (POMS) of randomized controlled trials receiving either probiotics or placebo treatment. Murata 2018 [54], Ohsawa 2018 [58], Sashihara 2013 [69], Shinkai 2013 [72].

Mood states: The Profile of Mood States (POMS) questionnaire was investigated by four studies and at qualitative level no differences between the probiotic and placebo groups were observed. In the meta-analysis, as shown in Figure 4, the placebo interventions were in all subscales slightly in favor when compared to the probiotic groups, while the heterogeneity was low and never higher than I^2^ = 13%. All participants were healthy and without any diagnosed psychiatric disorder.

**Figure 5:**
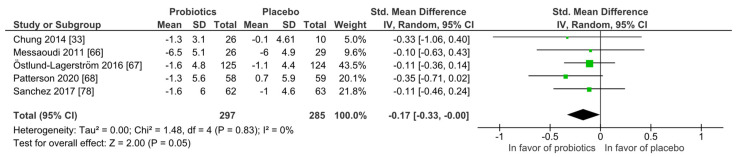
Quantitative analysis for Perceived Stress scale (PSS) of randomized controlled trials receiving either probiotics or placebo treatment. Chung 2014 [36], Messaoudi 2011 [52], Östlund-Lagerström 2016 [59], Patterson 2020 [60], Sanchez 2017 [68].

Stress: Similar to mood, none of the five studies found superiority of the probiotic intervention when applying the Perceived Stress scale (PSS) at qualitative level. With the meta-analysis (Figure 5), the probiotic interventions were slightly in favor when compared to the placebo groups (mean PSS difference −0.17 (95% CI: −0.33, −0.00); *p* = 0.05), while the heterogeneity was very low with I^2^ = 0%. All participants were healthy and without any diagnosed psychiatric disorder.

#### 3.3.2. Anxiety, Depression and Psychiatric Distress

Anxiety: Overall, anxiety improved in both groups, regardless of group allocation. The most used questionnaires for detecting anxiety in the study samples when data were available were the Beck Anxiety Inventory (BAI), the Hospital Anxiety Scale (HADS-A), the Hamilton Anxiety Rating Scale (HAM-A) and the State-Trait Anxiety Inventory (STAI; state and trait score). From the review sample of 54 trials, the mentioned questionnaires were collected and the results presented for 41 instances from 23 studies. The reason for this is that six studies used different questionnaires for the same outcome [30,49,55,56,60,70].

For the group comparisons (probiotic versus placebo treatment) at qualitative level, 32 instances (78%) showed no differences between the groups and nine studies were in favor of the probiotic group (22%). Of these, one study was conducted in patients with diagnosed anxiety [30]. Here several questionnaires were applied and no difference between groups was reported for BAI and STAI trait, but there was superiority for the probiotic intervention for the STAI state.

**Figure 6:**
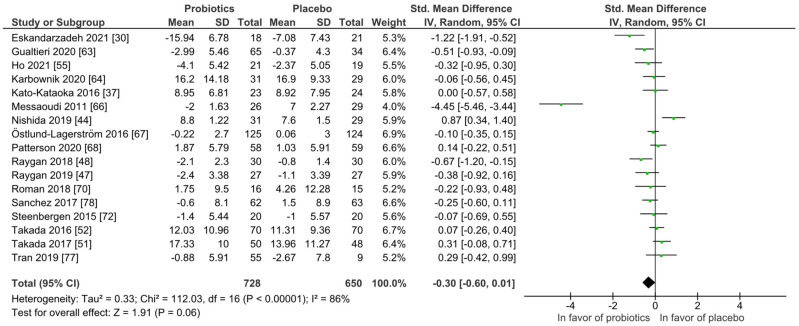
Quantitative analysis for anxiety of randomized controlled trials receiving either probiotics or placebo treatment. Eskandarzadeh 2021 [30], Gualtieri 2020 [42], Ho 2021 [43], Karbownik 2020 [46], Kato-Kataoka 2016 [47], Messaoudi 2011 [52], Nishida 2019 [56], Östlund-Lagerström 2016 [59], Patterson 2020 [60], Raygan 2018 [62], Raygan 2019 [63], Roman 2018 [65], Sanchez 2017 [68], Steenbergen 2015 [73], Takada 2016 [74], Takada 2017 [75], Tran 2019 [76].

For quantitative analysis, 17 studies remained and the results are presented as forest plots in Figure 6. The probiotic intervention was not in favor compared to the placebo group (mean difference −0.30 (95% CI: −0.60, 0.01); *p* = 0.06) accompanied with high heterogeneity (I^2^ = 86%). To reduce heterogeneity, subgroup analyses were performed for study length, probiotic strain numbers, country, application form and health status as presented in Figures S1–S6, not changing the outcome of the full analysis presented in Figure 6. Heterogeneity dropped considerably only in the single-strain subgroup with I^2^ = 52%.

Depression: Overall, depression improved in both groups, regardless of group allocation. The most used questionnaires for detecting depression were the Beck Depression Inventory (BDI), Hospital Depression Scale (HADS-D) and Hamilton Depression Rating Scale (HAM-D). From the review sample of 54 trials, 19 studies reported findings for the above-mentioned questionnaires. Among these, one study used more than one questionnaire for the same outcome [64].

For the group comparisons at qualitative level, 12 studies (63%) showed no differences between the groups and 7 studies were in favor of the probiotic group (37%). Of these, 4 studies were conducted in patients with diagnosed depression. Half of the studies found no group differences and the other half favored the probiotic group.

**Figure 7:**
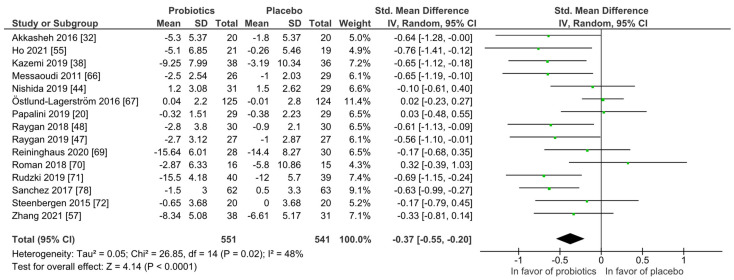
Quantitative analysis for depression of randomized controlled trials receiving either probiotics or placebo treatment. Akkasheh 2016 [32], Ho 2021 [43], Kazemi 2019 [48], Messaoudi 2011 [52], Nishida 2019 [56], Östlund-Lagerström 2016 [59], Papalini 2019 [20], Raygan 2018 [62], Raygan 2019 [63], Reininghaus 2020 [64], Roman 2018 [65], Rudzki 2019 [67], Sanchez 2017 [68], Steenbergen 2015 [73], Zhang 2021 [79].

For quantitative analysis, 15 studies were available and the results are presented as forest plots in Figure 7. The probiotic interventions were favorable when compared to the placebo groups (mean difference −0.37 (95% CI: −0.55, −0.20); *p* ≤ 0.0001) with small effect size, while the heterogeneity was moderate with I^2^ = 48%. For a differentiated understanding of the findings, subgroup analyses for probiotic strain numbers, country, application form and health status were performed and presented in Figures S7–S12. Heterogeneity decreased remarkably in the multi-strain subgroup (I^2^ = 4%), the Asia subgroup (I^2^ = 0%) and the subgroup with diagnosed depression disorder (I^2^ = 0%).

Psychiatric Distress: Overall, psychiatric distress improved in both groups, regardless of group allocation. The most used questionnaires for detecting psychiatric distress were the General Health Questionnaire (GHQ) and the Symptoms Checklist-90 (SCL-90). From the review sample of 54 trials, 14 studies reported findings of the above-mentioned questionnaires. 

For the group comparisons (probiotic versus placebo treatment) at qualitative level, 12 studies (85.7%) showed no differences between the groups and two studies were in favor of the probiotic group (14.3%). Of these, one study was conducted in patients with diagnosed depression and no group differences were reported.

**Figure 8:**
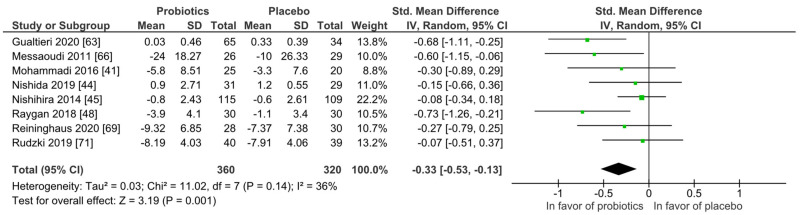
Overview of psychiatric distress symptom outcomes according to questionnaire. Gualtieri 2020 [42], Messaoudi 2011 [52], Mohammadi 2016 [53], Nishida 2019 [56], Nishihira 2014 [57], Raygan 2018 [62], Reininghaus2020 [64], Rudzki 2019 [67].

For quantitative analysis, eight studies were available, and the results are presented as forest plots in Figure 8. The probiotic interventions were favorable when compared to the placebo groups (mean difference −0.33 (95% CI: −0.53, −0.13); *p* = 0.001) with small effect size, while the heterogeneity was moderate with I^2^ = 36%. For a differentiated understanding of the findings, subgroup analyses for study strain numbers, country, application form and health status were performed and presented in Figures S13–S18. Heterogeneity decreased remarkably in the short-term treatment subgroup of 4 to 8 weeks (I^2^ = 0%), and the subgroup with the application form capsules/pills (I^2^ = 0%).

#### 3.3.3. Central Nervous System (CNS) Findings in EEG and Brain Imaging

Six studies recorded EEG, three of them during sleep to assess the effects of probiotics on sleep parameters [43,56,75]; these studies reported improvements of sleep quality. Two further studies found that probiotic intake improved memory function [34] and modulated brain regions associated with relaxation and attention [33]. In contrast, Kelly et al. (2017) found no significant difference between the probiotic and placebo treatment in the EEG measurements for cognitive functions in hypothalamic–pituitary adrenal response while socially evaluated cold pressor test, paired associates learning, attention witching task, emotional troop and emotion recognition task [49].

In total, five imaging studies were found. Tillisch et al. (2013) investigated the effects of a multi-strain probiotic using fMRI during identification of emotional face expressions and found that probiotic treatment affected brain activity of regions that control central processing of emotion and sensation [21]. Bagga et al. (2018, 2019) published two fMRI studies using the same multi-strain probiotic and found that the probiotic improved memory performance and altered brain activation patterns [19] and that behavior was modulated towards a shift of efficient attentional control and changes in functional, but not structural, connectivity [18]. Papalini et al. (2019) investigated the effects of a multi-strain probiotic by fMRI using the emotional face-matching, emotional face–word Stroop and the classic color–word Stroop paradigms and found no difference between probiotic and placebo treatment. [20]. Finally, the next generation probiotic *Bifidobacterium longum 1714* was tested under social stress (octracism) conditions by (Wang et al. (2019)) by applying the cyber ball game and was proved to reduce stress effects in a magnetoencephalography paradigm [22].

### 3.4. Risk of Bias

The risk of bias assessment is presented in Table 2. The overall risk of bias was low in 18 studies (33.3%), with some concerns in 18 studies (33.3%) and high in 18 studies (33.3%). Overall, the studies with intention-to-treat-analysis (ITT), there was a low risk of bias in 11 studies (42.3%), some concerns in 7 studies (26.9%) and in 8 studies high (30.8%). While in the trials with per-protocol-analysis (PP), 7 studies had a low bias (25%), 11 had some concerns (39.3%) and in 10 studies the risk of bias was high (35.7%).

For studies included in the meta-analysis, the overall risk of bias was low in 9 studies (30%), with some concern in 9 studies (30%) and high in 12 studies (40%). Eighteen of the trials were analyzed per protocol and not per intention-to-treat. While most of the intention-to-treat studies remained with low risk of bias (5 of 12, 41.7%), the per-protocol studies had six studies with some concerns (33.3%) and eight with a high risk of bias (44.4%).

## 4. Discussion

This systematic review and meta-analysis investigated the effects of probiotic treatment on (i) psychological well-being (mood and stress), (ii) psychiatric symptoms (anxiety, depression, psychiatric distress) and (iii) central functions.

At qualitative level we found that probiotic treatment mostly was not superior to placebo treatment when evaluating at questionnaire level. Only a fraction of these studies was included in meta-analysis following quality of study assessment.

Regarding mood and stress, at qualitative level the studies showed no superiority of the probiotic intervention and at quantitative level, placebo was slightly in favor for mood and the probiotics for stress, suggesting that psychological well-being at this level was not affected. Similarly, for psychiatric symptoms including anxiety, depression and psychiatric distress, most studies found no group differences at qualitative level. In line with this, at the quantitative level, no differences between groups were found for anxiety and, regarding depression and psychiatric distress, the probiotic group was only slightly in favor. However, also bearing in mind the findings at the qualitative level, the clinical relevance of these findings is questionable. Nevertheless, it needs to be taken in account that only a fraction of studies included a patient collective. For patients with diagnosed depression, the small effect of probiotic treatment on symptom improvement was stable when considering the substantially low heterogeneity.

In addition, EEG and imaging studies summarized in this review proved that probiotics are able to also exert effects on CNS function in humans and not only in pre-clinical models, although the number of studies is still low. Thus, there appears to be potential for probiotics in this area for prevention and treatment, but to which extent and in which specific areas such an approach is promising is unclear to date.

Experience from extensive probiotic research in irritable bowel syndrome and other GI-related disciplines over the last four decades shows that the results are still conflicting due to several reasons. One is that most probiotics are marketed as nutritional supplements [80] and not as drugs [12]. As a consequence of this, clinical trials usually do not match all standards imposed by the US Food and Drug Administration (FDA) or European Medical Agency (EMA) [80]. Furthermore, not all probiotics can be expected to be of similar efficacy (number of strains, type of strain(s)) [81], and they may not be effective across different patient collectives and even patient subgroups [12]. What is more, in the field of probiotic research in the GI-related disciplines, effects of probiotics on psyche were not thoroughly investigated in the past and, if reported, no effects were observed [26]. At this point it should be considered that if probiotics exert clinical meaningful psychological effects, it is unlikely that no one has noticed this within 40 years of research. However, it needs to be also considered that these probiotics were not clearly defined for targeting CNS function. Additionally, many traditional multi-strain products are used with the interactions between the strains having been rarely or not at all investigated. The multi-strain probiotics mostly have their origin in the milk industry where probiotic mixtures were composed due to practical reasons. Probiotic research has the potential to improve in all fields, with next generation probiotics being now available. These probiotics are derived from next generation sequencing and are selected for specific properties and specific indication [82]. In this area of research, next generation probiotics selected and developed for their ability to improve psychiatric condition and potentially other CNS functions are suitable. In this review, with regard to the imaging studies, only one study investigated the effects of such a next generation probiotic and found stress-reducing effects under social stress conditions [22]. In IBS (reviewed elsewhere [26]), one more next generation probiotic was tested using fMRI and found reduced limbic reactivity towards fearful stimuli [23]. Both studies hint at the potential of these probiotics for modulating mood and stress in health and disease. Whether or not they have the potential to also influence pathological mental health states is completely unclear. If yes, it is most likely that probiotics themselves may not deliver satisfying results, warranting a multicomponent treatment.

This study has strength and limitations. A clear strength is the methodological approach taken according to PRISMA criteria. To provide homogeneity of the trials, the search was limited to RCTs in adults only using probiotics and postbiotics, but neither synbiotics nor prebiotics. Still, despite clear eligibility criteria, the studies were heterogeneous regarding study design, probiotic strain(s), application form and duration, sample population and outcomes. Since many studies were not able to report positive outcomes for the probiotic intervention, the data were often not reported and the result was only mentioned in the text, not allowing us to include the findings in the meta-analysis. Thus, we contacted the corresponding authors at least three times to obtain as many data for meta-analysis as possible. However, we were not always successful, and two studies could not provide the data because the sponsor had not agreed. To be as objective as possible, we therefore always reported the data at both the qualitative and quantitative level. Overall, the heterogeneity of the meta-analyses was acceptable and only for anxiety were the findings high. To reduce heterogeneity and for a more detailed understanding of the data, subgroup analyses were performed. These further reduced heterogeneity in several cases and also allowed us to get insights on the role of participant status (patient versus healthy) in these analyses.

## 5. Conclusions

Overall, we found that probiotics have the potential to exert effects on CNS function in humans. However, most probiotics did not affect mood, stress, anxiety, depression and psychiatric distress when compared to placebo at the qualitative level. At quantitative level, depression and psychiatric distress improved slightly in the probiotic condition. It remains unclear to which extent next generation probiotics selected and developed for their ability to improve psychiatric condition and potentially other CNS functions (sometimes called psychobiotics), are beneficial for improving mental well-being and psychiatric symptoms in health and disease.

## Figures and Tables

**Table 1:** Trial characteristics of single studies. A summary of the raw data as direction of change of the applied questionnaires are presented. It is questionnaire specific whether or not a decrease implies improvement or deterioration and accordingly, whether or not an increase implies improvement or deterioration. Psychiatric symptoms and CNS changes in the probiotic versus the placebo group: ↔: no statistical differences between the groups; ↓: significant reduction *p* < 0.05; ↓↓: significant reduction *p* < 0.01; ↓↓↓: significant reduction *p* < 0.001; ↑: significant increase *p* < 0.05; ↑↑: significant increase *p* < 0.01; A: analyzed; αLA: α-Lactalbumin; AUT: Austria; CAN: Canada; CFU: Colony forming unit; CHN: China; CN: Control group; CTG: Combined treatment group; DEU: Deutschland; DSM-5: American Diagnostic and Statistical Manual of Mental Disorders (5th Edition); DTG: Dietary treatment group (hypocaloric diet); ESP: Spain; FMS: Fibromyalgia; FRA: France; IRL: Ireland; IRN: Iran; ITA: Italy; IU: International Unit; JPN: Japan; KOR: Korea; LHFM: Lactobacillus helveticus-fermented milk; LP: Lactobacillus paracasei MCC1849; MCI: Mild cognitive impairment; MDD: Major depressive disorder; MYS: Malaysia; N: Number; NI: No intervention group; NLD: Netherlands; NR: Not reported; NWL: Normal weight lean; NWO: Normal weight obese; NZL: New Zealand; PL: Placebo group; POL: Poland; PR: Probiotic group; PRE: Prebiotic group; PreobOB: Pre-obese/obese; RCT: Randomized controlled trial; SEM: Standard error of mean; SE: Standard error; SD: Standard deviation; SSRI: Selective Serotonin Reuptake Inhibitor; SWE: Sweden; TWN: Taiwan; VAS: Visual Analog Scale. More explanations are provided in Table S2.

Author (Year)	Country	Subject	Intake Length (Week)	SampleSize (A); Sex (f%); AgeMean (SD); HealthCondition; Groups (N)	Probiotic Species (N); Dose; Times Intake per Day; Application	Outcomes
Akkasheh et al. (2016) [32]	IRN	Depression	8	40 (40); f: 85%; age: PL→36.2 (8.2), PR→38.3 (12.1); MDD; groups: PL (20), PR (20)	Tak Gen Zist Pharmaceutical Company (**3**); each 2 × 10^9^ CFU/g; 1/day; capsule	Behavioral: BDI ↓
Adikari et al. (2020) [33]	MYS	Anxiety	8	20 (19); f: 0%; age: PL→19 ± 0.66, PR→19 ± 0.81; healthy; groups: PL (9), PR (10)	*Lactobacillus casei Shirota* (**1**); 3 × 10^10^ CFU; 1/day; liquid	Neuropsychological: DVT reaction time ↓, accuracy percentage ↔ Neurophysiological: EEG ↔ (week 8), ↑ theta brain wave + delta brain wave for probiotic group (week 4); EDR ↔
Allen et al. (2016) [34]	IRL	Stress	8	27 (22); f: 0%; age: 25.5 (1.2); healthy; RCT Crossover : 4 weeks placebo, 4 weeks probiotics, no washout	*Bifidobacterium longum 1714* (**1**); 1 × 10^9^ CFU; 1/day; powder	Behavioral: Cohen PSS: ↓; STAI: ↔ Neuropsychological: PAL (total errors): ↔ Neurophysiological: EEG Mobility ↑, Cz theta power ↓
Bagga et al. (2018) [19]	AUT	Emotional brain signatures	4	45 (NR); f: 48.9%; age: PL→27.25 (5.78), PR→28.27 (4.2), NI→26.87 (4.97); healthy; groups: PL (15), PR (15), NI (15)	Ecologic 825 by Winclove Probiotics (**9**); 7.5 × 10^9^ CFU/g; 1/day; powder	Behavioral: Post-hoc comparisons: PANAS ↑↑; SCL-90 ↔; ADS ↔; LEIDS: Subscales hopelessness ↓↓ and risk aversion ↓; PRP with less decision change for unpleasant stimuli ↑↑ RAU (response accuracy for unpleasant stimuli) Neurophysiological: fMRI BOLD contrast correlated with PANAS positive score ↑; Emotional decision-making task (neutral > baseline) contrast: significant differences (p value NR) in brain activity in left anterior cingulum compared to NI
Bagga et al. (2019) [18]	AUT	Functional connectivity in brain	4	45 (NR); f: 51.1%; age: 26.24 (4.76); healthy; groups: PL (15), PR (15), CN (15)	Ecologic 825 by Winclove Probiotics (**9**); 7.5 × 10^6^ CFU/g; 1/day; powder	Neurophysiological: significant changes in the functional connectivity (FC) comparing PRP with CON and PLP comparing to CON: ↑ Salience network in Cingulate gyrus + Precuneus cortex), ↓ Middle and superior frontal gyrus network in Frontal pole + Frontal medial cortex; comparing to PLP: ↓ Visual network in Postcentral gyrus + Precuneus and Default mode network in Frontal pole, Superior frontal gyrus + Paracingulate gyrus probiotic intervention did not influence the structural connectivity
Benton et al. (2007) [35]	UK	Mood and Cognition	3	132 (126); f: 59.52%; age: 61.8 ± 7.3; healthy; groups: NR	*Lactobacillus casei Shirota* (**1**); 6.5 × 10^9^ CFU; 1/day; liquid	Behavioral: POMS: ↔ Neuropsychological: Memory: Wechsler Memory scale, Retrieval from long-term memory, Test of verbal fluency: ↔; NART: converted to Z scores: ↔
Chung et al. (2014) [36]	KOR	Cognitive Fatigue	12	39 (36); f: 44.4%; age: 65.00 ± 4.14; >24 in MMSE-K; groups: PL (10), LHFM 500 mg (10), LHFM 1000 mg (7), LHFM 2000 mg (9)	*Lactobacillus helveticus IDCC3801* (**1**); dose NR; 4/day; tablet	Behavioral: PSS ↔, GDS-SF ↔ Neuropsychological: DST, SRL, VLT, Serial 3 s and 7 s: ↔, RVIP: 1000 mg ↑ correct responses (post-hoc), ST: error responses 500 mg ↓
De Lorenzo et al. (2017) [37]	ITA	Psychological Profile	3	48 (48); f: 100%; age: NWL30.18 ± 2.04, NWO: 40.00 ± 12.54, PreobOB: 33.57 ± 10.57; healthy; groups: PL (24), PR (24); RCT Crossover: 3 weeks intervention, 3 weeks wash-out, 3 weeks other intervention	Biocult Strong, HOMEOSYN (**9**); each 1.5 × 10^10^ CFU/3 g; 1/day; powder	Behavioral: SCL90R: ↔; BUT ↓ (S)
Dickerson et al. (2014) [38]	USA	Schizophrenia	14	65 (65); f: 35.4%; age: PL→48.1 (9.4), PR→44.4 (11.0); diagnosed schizophrenia with antipsychotic medication; groups: PL (32), PR (33)	Bifiform Balance (**2**); each 10^9^ CFU; 1/day; tablet	Behavioral: PANSS ↔
Dickerson et al. (2018) [39]	USA	Acute Mania	24	66 (66); f: 63.6%; age: PL→33.3 (±13.3), PR→37.9 (±11.7); maniac episode; groups: PL (33), PR (33)	Chr. Hansen (**2**); >10^8^ CFU; 1/day; tablet	Behavioral: YMRS, MADRS, BPRS ↔
Diop et al. (2008) [40]	FRA	Stress	3	75 (75); f: 72%; age: 38 ± 11; healthy; groups: PL (37), PR (38)	Probio stick (**2**); 3 × 10^9^ CFU; 1/day; powder	Behavioral: Questionnaire assessing stress-induced symptoms (62 items) VAS 10 cm: ↓ abdominal pain, ↓ nausea/vomiting
Eskandarzadeh et al. (2021) [30]	IRN	Generalized Anxiety Disorder	8	48 (39); f: 81.25%; age: PL→33.67 ± 6.56, PR→34.17 ± 6.14; GAD-7 score > 7; groups: PL (24), PR (24); Intake with sertraline in both groups	Takgene Zist Company (**4**); 1.8 × 10^10^ CFU; 1/day; capsule	Behavioral: HAM-A ↓↓, State-Anxiety score ↓, Trait-Anxiety score ↔, BAI ↔, WHO-QOL-BREF ↔
Ghaderi et al. (2019) [41]	IRN	Schizophrenia	12	60 (60); f: 6.7%; age: PL→43.2 ± 6.0, PR→44.8 ± 8.3; diagnosed schizophrenia; groups: PL (30), PR (30); probiotic group within take of 50,000 IU of Vitamin D3 every 2 weeks	Lacto Care (**4**); each 2 × 10^9^ CFU; 1/day; capsule	Behavioral: PANSS ↓↓ in general and total subscore Neuropsychological: BPRS ↔
Gualtieri et al. (2020) [42]	ITA	Anxiety	12	142 (97); f: 61.9%; age: 41.29 (±14.9); healthy; groups: PL (71), PR (71)	Biocult Strong, HOMEOSYN (**9**); each 1.5 × 10^10^ CFU/3 g; 1/day; powder	Behavioral: HAM-A↓↓ (especially ↓ in IL-1β rs16944 carriers than in non-carrier), BUT ↔, SCL-90 ↔
Ho et al. (2021) [43]	TWN	Depression	4 (30 days)	40 (40); f: 67.5%; age: PL→25.47 ± 4.64, PR→26.43 ± 5.95; PSQI > 5, ISI > 13; and met the DSM-5 criteria for chronic primary insomnia; group: PL (19), PR (21)	*Lactobacillus plantarum PS128* (**1**); 3 × 10^10^ CFU; 2/day; capsule	Behavioral: BDI-II ↓, BAI ↔, PSQI ↔, ISI ↔, ESS ↔, VAS fatigued before sleep ↓↓ Neurophysiological: sleep EEG ↔ (During N1: theta power % ↓ at day 15)
Hwang et al. (2019) [44]	KOR	Mild Cognitive Impairment	12	100 (100); f: 66%; age: PL→69.2 (7.00), PR→68.0 (5.12); healthy with MCI; groups: PL (50), PR (50)	*DW2009 Lactobacillus plantarum C29* (**1**); 1.25 × 10^10^ CFU/g; 1/day; 2 capsules (800 mg)	Neuropsychological: VLT, ACPT, DST: ↑ combined cognitive function (subscore: Attention/Prefrontal function ↑ in composite score)
Inoue et al. (2018) [45]	JAP	Cognitive Function	12	39 (38); f: 64.1%; age: 70.3 (±3.1); healthy; groups: PL (19), PR (20); intake with physical training	Morinaga Milk Industry Co., Ltd. (**4**); each 1.25 × 10^10^ CFU; 1/day; powder	Behavioral: PHQ-9 ↔, GAD-7 ↔ Neuropsychological: MoCA-J ↔
Karbownik et al. (2020) [46]	POL	Stress	4	92 (92); f: 60%; age: 22.6 ± 1.3; healthy; groups: PL (29), PR-Lactobacillus (32), PR-Saccharomyces (31)	LacidoEnter (**1**): 5 × 10^9^; Dicoflor 60 (**1**): 6 × 10^9^; 1/day; capsule	Behavioral: STAI ↔ Neuropsychological: Performance in Academic Examination ↔
Kato-Kataoka et al. (2016) [47]	JAP	Stress	8	49 (47); f: 44.9%; age: PL→22.8 (±0.3), PR→22.8 (±0.4); healthy; groups: PL (25), PR (24)	*L.casei strain Shirota YIT 9029* (**1**); 1.0 × 10^11^ CFU; 1/day; liquid	Behavioral: feelings of stress by 100 mm VAS ↓, STAI ↔, NEO-FFI ↔
Kazemi et al. (2019) [48]	IRN	Depression	8	110 (110); f: 70.9%; age: 36.47 (8.03); mild to moderate major depression; groups: PL (36), PR (38), PRE (36)	Lallemand Health Solution (**2**); >10 × 10^9^ CFU; 1/day; powder	Behavioral: BDI ↓
Kelly et al. (2017) [49]	IRL	Stress and Cognitive Performance	8	29 (NR); f: 0%; age: 24.59 (0.75); healthy; groups: PL-PR (15), PR-PL (14); RCT Crossover: switch of treatment after 4 weeks, no washout	*Lactobacillus rhamnosus* (JB-1) (**1**); 1 × 10^9^ CFU; 1/day; capsule	Behavioral: BDI, BAI, PSS, STAI, SCL-90, PSQI, CCL: ↔ Neuropsychological: Test from CANTAB battery ↔; SECPT ↔ Neurophysiological: EEG ↔
Kobayashi et al. (2019) [50]	JAP	Memory	12	121 (117); f: 50.4%; age: PL→61.6 (6.37), PR→61.5 (6.83); subjective memory complaints and MMSE score 22–27; groups: PL (60), PR (61)	*Bifidobacterium breve A1* (**1**); >1 × 10^10^ CFU; 1/day; capsule	Neuropsychological: RBANS ↔, MMSE ↔
Lew et al. (2019) [51]	MYS	Stress, Anxiety, Memory, Cognition	12	132 (103); f: 76.7%; age: PL→32.1 ± 11.4, PR→31.3 ± 10.8; healthy with score of moderate stress level on PSS-10; groups: PL (51), PR (52)	*Lactobacillus plantarum P8* (**1**); 2 × 10^10^ CFU; 1/day; powder	Behavioral: PSS-10: ↔; DASS-42 total: ↓ week 4 + 12; DASS-42 stress: ↓ week 4, 8, 12; DASS-42 anxiety: ↓ week 4, 12; DASS-42 depression ↔ Neuropsychological: CBB speed for social emotional cognition (in women) ↓↓; international shopping list memory test ↑
Messaoudi et al. (2011) [52]	FRA	Anxiety, depression, stress and coping	4 (30 days)	55 (55); f: 74.5%; age: PL→43.2 (8.5), PR→42.4 (7.5); score of <12 in the HADS-anxiety subscale and the HADS-depression subscale; groups: PL (29), PR (26)	Institut Rosell-Lallemand (**2**); 3 × 10^9^ CFU; 1/day; powder	Behavioral: HSCL-90-GSI↓, HADS↓, HADS-A ↔, HADS-D ↔, PSS ↔, CCL ↔
Mohammadi et al. (2016) [53]	IRN	mental health	6	75 (70); f: 48.6%; age: PL→33.1 ± 6.1, PRyogurt→33.2 ± 6.4, PRcapsule→31.5 ± 5.8; healthy; groups: PL (20), PRyogurt (25), PRcapsule (25)	yogurt: Pegah Company (**2**), capsules: ZistTakhmir Co. (**7**); CFU: different, see Table S1; 1/day	Behavioral: GHQ ↔, DASS ↔
Murata et al. (2018) [54]	JAP	Mood States	12	241 (202); f: 100%; age: PL→20.2 ± 0.1, PR (10LP)→20.9 ± 0.5, PR (30LP)→21.1 ± 0.6; healthy; groups: PL (70), PR10LP (69), PR30LP (63)	LAC-Shield (**1**); 10PL: 1 × 10^10^ CFU, 30PL: 3 × 10^10^ CFU; 1/day; powder	Behavioral: POMS2 10LP group: ↓ T-scores for Friendliness week 6 + 12, ↓ T-scores for Vigour-Activity week 6
Nishida et al. (2017) [55]	JAP	Stress, Sleep Quality	5	32 (32); f: 34.4%; age: PL→21.31 ± 0.9, PR→34.4%; healthy; groups: PL (16), PR (16)	heat-inactivated *Lactobacillus gasseri CP2305* (**1**); 1 × 10^10^ CFU; 1/day; liquid	Behavioural: GHQ-28 total score ↔, ↑ somatic symptom score, significant interaction of treatment and sex; Zung-SDS ↔; HADS ↔; STAI ↔; PSQI ↔
Nishida et al. (2019) [56]	JAP	Stress	24	60 (NR); f: 31.7%; age: PL→25.3 ± 0.6, PR→24.9 ± 0.5; healthy; groups: PL (31), PR (29)	*Lactobacillus gasseri CP2305* (**1**); 1 × 10^10^ bacterial cells; 2 tablets 1/day	Behavioral: STAI-state ↔, STAI-trait ↓; GHQ28 total ↔, depression ↓; HADS ↔; PSQI ↓ Neurophysiological: EEG: REM and non-REM sleep times ↔, total delta power ↔, ↑ ration of EEG delta power in the first sleep cycle, ↓ sleep latency of the first N3 stage and wake time after sleep onset
Nishihira et al. (2014) [57]	JAP	Stress	12	238 (224); f: 69.2%; age: PL→54.25 ± 10.93, PR→53.61 ± 11.31; healthy; groups: PL (109), PR (115)	MegMilk Snow Brand Co. Ltd. (**2**); SBT2055 > 5 × 10^8^ CFU, SBT2928 > 1 × 10^9^ CFU; 1/day; yogurt	Behavioral: GHQ-28 ↔
Ohsawa et al. (2018) [58]	JAP	Cognitive Function	8	61 (60); f: 56.7%; age: PL→57.8 ± 5.9, PR→58.5 ± 6.5; healthy, baseline RBANS total score 29–52; groups: PL (29), PR (31)	*Lactobacillus helveticus CM4* (**1**); dose: NR; 1/day; liquid	Behavioral: POMS ↔ Neuropsychological: RBANS total ↔, five index scores: ↑ attention score, subtest index score: ↑ Coding
Östlund-Lagerström et al. (2016) [59]	SWE	Wellbeing	12	290 (249); f: PR→57%, PL→65.6%; age: PR→72.6 (5.8), PL→72 (5.6); mentally and physically fit; groups: PL (124), PR (125)	*Lactobacillus reuteri DSM 17938* (**1**); 1 × 10^8^ CFU; 2/day; powder	Behavioral: HADS ↔,PSS ↔
Papalini et al. (2019) [20]	NLD	Neurocognition	4 (28 days)	61 (58); f: 100%; age: PL→22 (SEM: 0.5), PR→21 (SEM: 0.4); healthy; groups: PL (29), PR (29)	Ecologic Barrier (**9**); 5 × 10^9^ CFU; 1/day; powder	Behavioral: BDI ↔, LEIDS-r ↔, BIS-BAS ↔ Neuropsychological: SECPT↔; Digit Span Test ↔, Emotional face-matching paradigm ↔, Emotional face-word stroop paradigm ↔, Classic colour-word stroop paradigm↔ Neurophysiological: fMRI ↔
Patterson et al. (2020) [60]	DEU	Stress, Anxiety	5	120 (117); f: 49.6%; age: PL→23.25 (4.20), PR→23.73 (4.27); healthy; groups: PL (59), PR (58)	*Lacticaseibacillus paracasei Lpc-37* (**1**); 1.75 × 10^10^ CFU; 1/day; capsule	Behavioral: STAI ↔, VAS Stress ↔, VAS Insecurity ↔ (↓male), VAS Anxiety ↔ (↓male), VAS Exhaustion ↔, PSS ↔ (↑ female), BAI ↔, DASS-24 ↔, Online Diary ↔ (Perceived Productivity + Perceived Health Status ↑ and Sleep Related Recovery ↑↑ in high chronic stress subgroup)
Rao et al. (2009) [61]	CAN	Chronic Fatigue Syndrome	8	39 (35); f: 77.1%; age: 18–65; healthy, diagnostic criteria for CFS; groups: PL (16), PR (19)	*Lactobacillus casei strain Shirota* (**1**); 8 × 10^9^ CFU; 3/day; powder	Behavioral: BDI ↔, BAI ↓
Raygan et al. (2018) [62]	IRN	Mental health	12	60 (60); f: 50%; age: PL→67.3 ± 11.0, PR→71.5 ± 10.9; diagnosed with type 2 diabetic and coronary heart disease (2–3 vessel); groups: PL (30), PR (30); Intervention Group with 50,000 IU vitamin D every 2 weeks	LactoCare (**4**); 8 × 10^9^ CFU; 1/day; application: NR	Behavioral: BDI ↓↓, BAI ↓↓, GHQ-28 ↓↓
Raygan et al. (2019) [63]	IRN	Mental health	12	60 (54); f: 61.1%; age: PL→62.4 ± 13.1, PR→64.8 ± 8.3; diagnosed with type 2 diabetic and coronary heart disease (2–3 vessel); groups: PL (27), PR (27); Intervention Group with 200 µg/day selenium	LactoCare (**4**); 8 × 10^9^ CFU; 1/day; application: NR	Behavioral: BDI ↓↓, BAI ↓, PSQI ↔
Reininghaus et al.(2020) [64]	AUT	Depression	4 (28 days)	82 (61); f: 77.0%; age: PL→40.11 (11.45), PR→43.00 (14.31); depressive episode by MINI; groups: PL (33), PR (28); bothgroups: 125 mg (D-Biotin) Vitamin D7	OMNi-BiOTiC Stress Repair (**9**); 7.5 × 10^9^ CFU; 1/day; powder	Behavioral: HAMD, BDI-II, SCL-90R, MSS, GLQI: ↔
Roman et al. (2018) [65]	ESP	Fibromyalgia	8	40 (31); f: 92.5%; age: PL→50.27 ± 7.86, PR→55.00 ± 8.37; diagnosed with FMS; groups: PL (20), PR (20)	ERGYPHILUS Plus (**4**); 6 × 10^6^ CFU; 4/day; capsule	Behavioral: STAI ↔, BDI ↔ Neuropsychological: MMSE ↔, two-choice task ↓, IGT ↔
Romijn et al. (2017) [66]	NZL	Depression	8	79 (79); f: 78.5%; age: PL→35.1 (14.5), PR→35.8 (14); healthy; ≥11 on QIDS-SR16 or ≥14 on DASS-42; groups: PL (39), PR (40)	Lallemand Health Solution (**2**); ≥ 3 × 10^9^ CFU; 1/day; powder	Behavioral: MADRS, iCGI, QIDS-SR16, GAF, DASS-42: ↔
Rudzki et al. (2019) [67]	POL	Depression	8	79 (60); f: 71.7%; age: PL→38.9 (12), PR→39.13 (9.96); major depression during SSRI monotherapy or drug free; groups: PL (39), PR (40); with SSRI treatment	Sanprobi IBS (**1**); 10 × 10^9^ CFU; 2/day; capsule	Behavioral: HAM-D 17 ↔, SCL-90 ↔, PSS-10 ↔ Neuropsychological: APT ↑↑, Stroop Test A + B ↔, RFFT ↔, TMT A + B ↔, CVLT ↑
Sanchez et al. (2017) [68]	CAN	Depression, Anxiety and Stress	24	126 (125); f: 61.6%; age: PL→37 ± 10, PR→35 ± 10; BMI between 29 and 41kg/m; groups: PL (63), PR (62)	*Lactobacillus rhamnosus CGMCC1.3724* (**1**); 3.24 × 10^8^ CFU/day; 2/day; capsules	Behavioral: BDI ↓, STAI ↔, PSS ↔
Sashihara et al. (2013) [69]	JAP	Mental Condition	4	44 (44); f: 0%; age: PL→20.2 ± 1.1, PR+αLA→19.9 ± 0.9, PR→19.8 ± 0.9; engaged in high-intensity training ≥5 days/week; groups: PL (14), PR (15), PR+αLA (15)	*Lactobacillus gasseri OLL2809* (**1**); 1 × 10^10^ CFU; 2 tablets 3/day	Behavioral: POMS and VAS for fatigue ↔
Sawada et al. (2017) [70]	JAP	Mental and Sleep Quality	4	24 (NR); f: 0%; age: NR; healthy; RCTCrossover; group1: placebo (4 weeks), washout (3 weeks), probiotic (4 weeks), group2: probiotic (4 weeks), washout (3 weeks), placebo (4 weeks)	*Lactobacillus gasseri CP2305* (**1**); 1 × 10^10^ CFU; 1/day; powder	Behavioral: GHQ28 ↔, Zung-SDS ↔, HADS depression + anxiety ↓, STAI state ↓, PSQI global + disturbance score ↓
Severance et al. (2017) [71]	USA	Schizophrenia	14	65 (56); f: 33.9%; age: PL→48.11 ± 9.6, PR→44.66 ± 11.4; diagnosis of schizophrenia or schizoaffective disorder; groups: PL (26), PR (30)	Bifiform Balance (**2**); each 10^9^ CFU; 1/day; application: NR	Behavioral: PANSS ↔
Shinkai et al. (2013) [72]	JAP	Mood and Quality of Life	20	300 (278); f: 50.4%; age: PL→70.9 ± 2.7, PRlowdose→71.0 ± 4, PRhighdose→70.8 ± 3.4; healthy; groups: PL (93), PRlowdose (92), PRhighdose (93)	*Lactobacillus pentosus strain b240* (**1**); low-dose: 2 × 10^9^ CFU, high dose: 2 × 10^10^ CFU; 1/day; tablets	Behavioral: POMS ↔, SF-36 ↓
Steenbergen et al. (2015) [73]	NLD	Sad Mood	4	40 (NR); f: 80%; age: PL→19.7 (1.7), PR→20.2 (2.4); healthy; groups: PL (20), PR (20)	Ecologic Barrier (**8**); 2.5 × 10^9^ CFU/g; 1/day; powder	Behavioral: LEIDS-r total ↓↓↓: aggression ↓↓; rumination ↓↓↓; BDI II ↔; BAI ↔
Takada et al. (2016) [74]	JAP	Stress	8	149 (140); f: 45.7%; age: PL→22.8 ± 0.2, PR→23.0 ± 0.2; healthy; groups: PL (70), PR (70)	*Lactobacillus casei YIT 9029* (**1**); 1 × 10^9^ CFU/mL; liquid (100 mL); 1/day	Behavioral: STAI ↔
Takada et al. (2017) [75]	JAP	Stress and Sleep	11	98 (94); f: 41.5%; age: PL→22.6 ± 0.2, PR→22.8 ± 0.2; healthy; groups: PL (48), PR (50)	*L. casei strain Shirota YIT 9029* (**1**); 1 × 10^9^ CFU/mL; 100 mL liquid; 1/day	Behavioral: GHQ ↔; NEO-FFI ↔; STAI ↔; PSQI ↔; total OSA ↔; subdivided factors (subsequently compared): sleepiness on rising ↑, sleep length↑↑ Neurophysiological: Sleep EEG: WASO ↑; N3 sleep ↓↓
Tillisch et al. (2013) [21]	USA	Brain Activity	4	36 (NR); f: 100%; age: 30 ± 10.4; healthy; groups: PL (11), PR (12), NI (13)	Danone Research facilities (**4**);dose: different, see Table S1; 2/day; liquid	Behavioral: Diary mood symptoms ↔; HAD ↔ Neurophysiological: Neuroimaging Acquisition and Analysis fMRI ↓↓↓ BOLD activity in the primary viscerosensory and somatosensory cortices
Tran et al. (2019) [76]	USA	Anxiety	4 (28 days)	90 (68); f: 75.6%; age: 20.59 (2.65); healthy; ConditionA (17): highCFU[50billion] + high species count [18], ConditionB (19): highCFU[50billion] + low species count [10], ConditionC (16): control/placebo group, ConditionD (19): lowCFU[15billion]+high species count[18], ConditionE (19): lowCFU[10billion]+low species count [10]	commercially available as over-the-counter products (e.g., Amazon) (**from 10 till 20**); 1 × 10^10^–5 × 10^10^ CFU; 1/day; pills	Behavioural: BAI, ACQ-R, PANAS, NMR, PSWQ ↔
Wang et al. (2019) [22]	DEU	Stress	4	43 (40); f: 65%; age: PL→33.00 ± 2.83, PR→31.00 ± 2.28; healthy; groups: PL (20), PR (20)	*Bifidobacterium longum 1714* (**1**); 1 × 109 CFU; 1/day; powder	Behavioural: SF-36↔ Neurophysiological: resting state MEG: ↑ theta band power, ↓ beta-3 band power in different brain region; during social distress: ↑ (S) theta band power,↑ alpha band power in different brain region; in both conditions’ inclusion/exclusion; NTS ↔; SEP ↔; MQ ↔
Xiao et al. (2020) [77]	JAP	Memory	16	80 (80); f: 51.3%; age: PL→60.9 (6.9), PR→61.3 (7.7); MMSE score 22 or more; group: PL (40), PR (40)	*Bifidobacterium breve A1* (**1**); 1 × 10^10^ CFU; 1/day; capsule	Neuropsychological: RBANS ↓↓↓ in RBANS total score, Immediate memory, Visuospatial/Constructional, Delayed memory; JMCIS ↔
Yamamura et al. (2009) [78]	JAP	Sleep	3	30 (29); f: 20.7%; age: PL→70.6 ± 5.65, PR→72.14 ± 5.67; healthy, no use of substances that influence sleep; groups: PLfirst (15), PRfirst (14); **RCT Crossover**→placebo first group: placebo (3 weeks), washout (3 weeks), probiotic (3 weeks), probiotic first group: probiotic (3 weeks), washout (3 weeks), placebo (3 weeks)	*Lactobacillus helveticus strain CM4* (**1**); dose: NR; 1/day; liquid	Behavioural: SHRI ↔, SF-36 ↔ Neurophysiological: Actigraphy ↔
Zhang et al. (2021) [79]	CHN	Depression	9	82 (69); f: 63.8%; age: PL→49.7 ± 9.6, PR→45.8 ± 12.3; diagnosed depression (DSM-5); groups: PL (31), PR (38)	*Lacticaseibacillus paracasei YIT 9029* (strain Shirota: LcS) (**1**); 1.0 × 10^10^ CFU; 1/day; liquid	Behavioural: BDI, HAMD: decreased significantly in both groups, no comparison between groups

**Table 2:** Risk of Bias. +: Low risk, !: Some concerns, −: High risk, D1: Randomisation process, D2: Deviations from the intended interventions, D3: Missing outcome data, D4: Measurement of the outcome, D5: Selection of the reported result.

Intention-to-Treat	D1	D2	D3	D4	D5	Overall
Akkasheh 2016 [32]	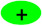	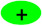	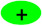	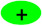	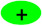	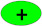
Allen 2016 [59]	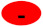	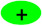	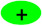	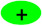	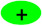	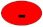
Bagga 2018 [19]	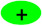	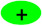	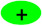	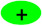	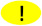	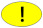
Bagga 2019 [18]	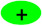	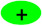	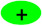	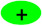	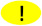	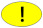
Benton 2007 [60]	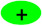	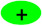	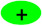	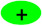	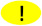	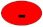
De Lorenzo 2017 [61]	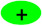	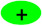	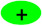	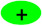	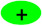	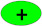
Dickerson 2018 [73]	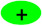	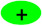	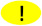	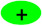	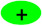	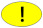
Diop 2008 [62]	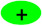	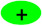	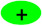	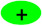	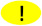	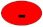
Ghaderi 2019 [34]	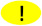	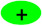	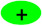	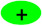	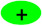	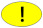
Ho 2021 [55]	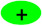	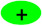	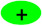	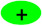	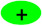	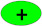
Hwang 2019 [35]	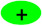	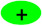	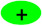	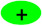	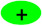	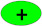
Karbownik 2020 [64]	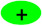	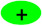	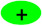	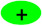	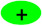	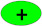
Kato-Kataoka 2016 [37]	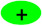	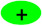	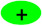	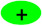	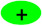	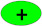
Kazemi 2019 [38]	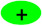	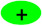	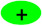	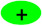	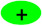	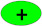
Kelly 2017 [65]	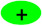	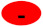	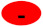	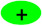	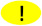	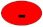
Nishida 2017 [43]	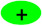	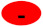	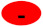	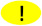	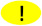	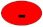
Nishida 2019 [44]	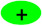	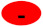	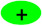	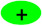	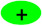	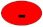
Östlund-Lagerström 2016 [67]	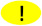	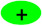	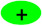	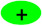	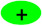	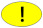
Patterson 2020 [68]	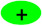	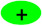	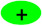	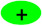	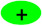	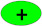
Raygan 2018 [48]	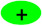	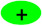	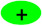	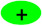	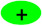	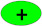
Romijn 2017 [79]	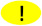	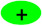	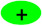	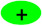	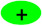	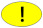
Sanchez 2017 [78]	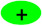	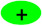	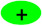	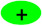	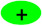	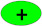
Sashihara 2013 [49]	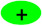	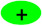	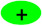	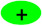	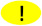	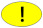
Sawada 2017 [50]	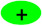	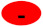	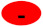	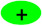	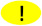	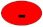
Steenbergen 2015 [72]	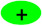	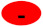	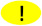	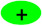	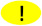	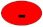
Xiao 2020 [56]	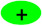	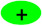	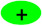	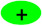	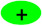	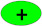
**Per-protocol**	**D1**	**D2**	**D3**	**D4**	**D5**	**Overall**
Adikari 2020 [54]	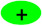	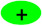	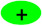	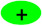	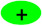	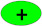
Chung 2014 [33]	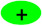	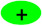	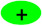	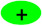	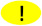	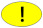
Dickerson 2014 [74]	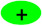	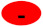	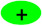	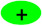	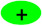	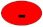
Eskandarzadeh 2021 [30]	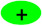	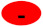	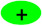	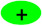	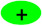	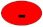
Gualtieri 2020 [63]	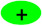	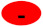	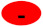	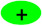	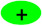	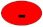
Inoue 2018 [36]	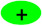	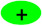	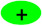	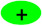	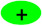	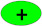
Kobayashi 2019 [39]	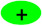	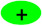	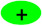	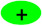	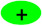	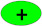
Lew 2019 [40]	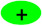	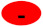	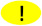	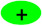	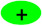	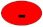
Messaoudi 2011 [66]	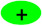	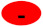	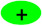	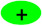	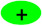	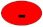
Mohammadi 2016 [41]	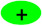	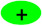	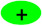	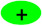	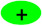	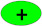
Murata 2018 [42]	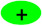	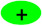	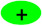	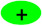	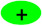	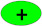
Nishihira 2014 [45]	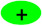	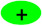	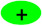	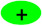	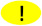	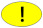
Ohsawa 2018 [46]	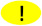	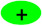	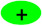	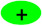	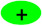	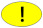
Papalini 2019 [20]	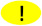	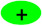	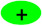	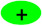	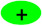	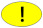
Rao 2009 [75]	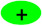	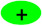	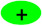	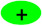	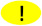	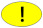
Raygan 2019 [47]	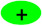	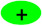	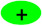	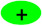	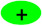	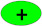
Reininghaus2020 [69]	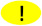	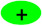	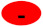	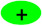	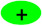	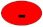
Roman 2018 [70]	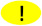	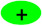	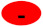	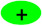	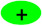	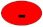
Romijn 2017 [79]	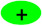	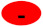	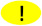	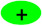	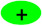	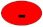
Rudzki 2019 [71]	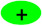	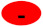	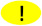	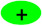	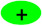	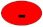
Shinkai 2013 [58]	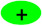	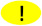	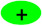	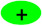	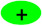	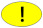
Takada 2016 [52]	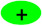	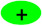	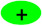	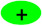	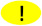	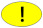
Takada 2017 [51]	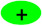	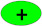	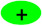	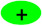	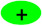	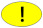
Tillisch 2013 [21]	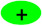	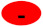	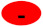	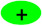	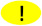	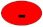
Tran 2019 [77]	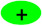	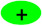	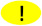	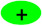	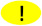	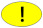
Wang 2019 [22]	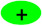	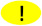	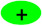	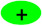	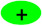	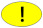
Yamamura 2009 [53]	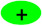	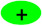	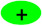	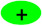	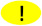	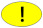
Zhang 2021 [57]	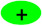	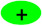	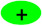	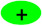	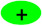	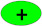
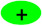	Low risk					
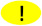	Some concerns					
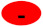	High risk					
D1	Randomisation process					
D2	Deviations from the intended interventions					
D3	Missing outcome data					
D4	Measurement of the outcome					
D5	Selection of the reported result					

## Data Availability

All data are reported in the manuscript and the Supplementary Materials.

## Supplementary Materials

The following supporting information can be downloaded at: https://www.mdpi.com/article/10.3390/nu14030621/s1, Text S1: Search Strategy, Table S1: Bacterial Species of Probiotics, Table S2: Questionnaires and tests applied by the included studies, Table S3: Study characteristics across studies, Table S4: Overview of the most used questionnaires and their results of all included studies, Table S5: Study outcomes at qualitative level for the most used questionnaires of the studies included in the meta-analysis, Figure S1: Effect of intake period on anxiety of randomized controlled trials receiving either probiotics or placebo treatment, Figure S2: Effect of single-strain versus multi-strain probiotics on Anxiety in randomized controlled trials receiving either probiotics or placebo treatment, Figure S3: Effect of studies from Asia versus studies from Europe on anxiety in randomized controlled trials receiving either probiotics or placebo treatment, Figure S4: Effect of application form on anxiety of randomized controlled trials receiving either probiotics or placebo treatment, Figure S5: Effect of studies with healthy participants on anxiety in randomized controlled trials receiving either probiotics or placebo treatment, Figure S6: Overview of anxiety symptom outcomes according to questionnaire, Figure S7: Effect of intake period on depression of randomized controlled trials receiving either probiotics or placebo treatment, Figure S8: Effect of single-strain versus multi-strain probiotics on depression in randomized controlled trials receiving either probiotics or placebo treatment, Figure S9: Effect of studies from Asia versus studies from Europe on depression in randomized controlled trials receiving either probiotics or placebo treatment, Figure S10: Effect of application form on depression of randomized controlled trials receiving either probiotics or placebo treatment, Figure S11: Effect of studies with healthy participants on depression in randomized controlled trials receiving either probiotics or placebo treatment, Figure S12: Overview of depression symptom outcomes according to questionnaire, Figure S13: Effect of intake period on psychiatric distress of randomized controlled trials receiving either probiotics or placebo treatment, Figure S14: Effect of single-strain versus multi-strain probiotics on psychiatric distress in randomized controlled trials receiving either probiotics or placebo treatment, Figure S15: Effect of studies from Asia versus studies from Europe on psychiatric distress in randomized controlled trials receiving either probiotics or placebo treatment, Figure S16: Effect of application form on psychiatric distress of randomized controlled trials receiving either probiotics or placebo treatment, Figure S17: Effect of studies with healthy participants on psychiatric distress in randomized controlled trials receiving either probiotics or placebo treatment, Figure S18: Overview of psychiatric distress symptom outcomes according to questionnaire.
