# Supplementary material for: Effect of Probiotics on Psychiatric Symptoms and Central Nervous System Functions in Human Health and Disease: A Systematic Review and Meta-Analysis
Source: Nutrients. 2022 Jan 30;14(3):621. doi: 10.3390/nu14030621 (PMC8839125; doi:10.3390/nu14030621)

Effect of Probiotics on Central Nervous System Functions in health and disease of humans: A Systematic Review and Meta-Analysis

Charlotte Le Morvan de Sequeira[1], Charlotte Hengstberger[1], Paul Enck[1] and Isabelle Mack[1]*

[1]Department of Psychosomatic Medicine and Psychotherapy, University Medical Hospital, 72076 Tübingen, Germany
*Correspondence: isabelle.mack@uni-tuebingen.de

SUPPORTING INFORMATION:

Text S1. Search Strategy.

PubMed-
(((("Healthy Volunteers"[Mesh] OR Healthy*[tiab]) OR ("Fatigue Syndrome, Chronic"[Mesh] OR Chronic Fatigue Syndrome*[tiab] OR CFS[tiab] OR "Anxiety Disorders"[Mesh] OR Agoraphobia*[tiab] OR Separation Anxiety[tiab] OR Neurocirculatory Asthenia[tiab] OR Effort Syndrome[tiab] OR Cardiac Neurosis[tiab] OR Cardiac Neuroses[tiab] OR Hyperkinetic Heart Syndrome[tiab] OR Neurotic Disorder*[tiab] OR Psychoneuroses[tiab] OR Neuroses[tiab] OR Obsessive Compulsive Disorder*[tiab] OR Anankastic Personality[tiab] OR Hoarding Disorder*[tiab] OR Panic Disorder*[tiab] OR Panic Attack*[tiab] OR Phobic Disorder*[tiab] OR Phobic Neuroses[tiab] OR phobic neurosis[tiab] OR Phobia*[tiab] OR Claustrophobia*[tiab] OR Social Anxiety Disorder*[tiab] OR "Bipolar and Related Disorders"[Mesh] OR Bipolar Disorder*[tiab] OR Manic Depressive Psychosis[tiab] OR Bipolar Affective Psychosis[tiab] OR Manic Depressive Psychoses[tiab] OR Psychoses, Manic Depressive[tiab] OR Mania*[tiab] OR Manic State*[tiab] OR Bipolar Depression*[tiab] OR Manic Disorder*[tiab] OR ("Feeding and Eating Disorders"[Mesh] NOT ("Feeding and Eating Disorders of Childhood"[Mesh] OR "Pica"[Mesh] OR "Relative Energy Deficiency in Sport"[Mesh] OR "Rumination Syndrome"[Mesh])) OR Eating Disorder*[tiab] OR Appetite Disorder*[tiab] OR Anorexia Nervosa[tiab] OR Avoidant Restrictive Food Intake Disorder[tiab] OR Food Neophobia[tiab] OR Food Neophobic[tiab] OR ARFID[tiab] OR Binge Eating Disorder*[tiab] OR Bulimia Nervosa[tiab] OR Diabulimia[tiab] OR Food Addiction*[tiab] OR Night Eating Syndrome[tiab] OR "Mood Disorders"[Mesh] OR Mood Disorder*[tiab] OR Affective Disorder*[tiab] OR Cyclothymic Disorder*[tiab] OR Depression*[tiab] OR Depressive[tiab] OR Dysthymic Disorder*[tiab] OR Premenstrual Dysphoric Disorder[tiab] OR "Seasonal Mood Disorder"[tiab] OR "Seasonal Mood Disorders"[tiab] OR Seasonal Affective Disorder*[tiab] OR "Neurocognitive Disorders"[Mesh:NoExp] OR Neurocognitive Disorder*[tiab] OR Traumatic Psychoses[tiab] OR "Amnesia"[Mesh] OR Amnesia*[tiab] OR ("Cognition Disorders"[Mesh] NOT "Huntington Disease"[Mesh]) OR Cognition Disorder*[tiab] OR Overinclusion*[tiab] OR Auditory Perceptual Disorders[tiab] OR "Auditory Perceptual Disorder"[tiab] OR Auditory Processing Disorder*[tiab] OR "Auditory Comprehension Disorders"[tiab] OR Cognitive Dysfunction*[tiab] OR Cognitive Impairment*[tiab] OR Cognitive Decline*[tiab] OR Mental Deterioration[tiab] OR Postoperative Cognitive Complications[tiab] OR Consciousness Disorder*[tiab] OR "Neurodevelopmental Disorders"[Mesh] OR (Neurodevelopmental Disorder*[tiab] NOT ("Stereotypic Movement Disorder"[Mesh] OR "Tic Disorders"[Mesh])) OR Separation Anxiety[tiab] OR Attention Deficit Disorders with Hyperactivity[tiab] OR Attention Deficit Hyperactivity Disorder*[tiab] OR Hyperkinetic Syndrome[tiab] OR ADDH[tiab] OR Attention Deficit Hyperactivity Disorder[tiab] OR Attention Deficit Disorder*[tiab] OR Minimal Brain Dysfunction[tiab] OR Conduct Disorder*[tiab] OR Child Behavior Disorders[tiab] OR Autism Spectrum Disorder*[tiab] OR Asperger*[tiab] OR Autism[tiab] OR Autistic[tiab] OR Communication Disorder*[tiab] OR "Communicative Dysfunction"[tiab] OR "Communicative Dysfunctions"[tiab] OR Communication Disabilit*[tiab] OR Fluency Disorder*[tiab] OR Speech Sound Disorder*[tiab] OR Phonological Disorder*[tiab] OR Developmental Disabilit*[tiab] OR Intellectual Disabilit*[tiab] OR Intellectual Development Disorder*[tiab] OR Mental Retardation[tiab] OR Idiocy[tiab] OR Mental Deficienc*[tiab] OR Learning Disabilit*[tiab] OR Learning Disorder*[tiab] OR Learning Disturbance*[tiab] OR Dyscalculia*[tiab] OR Acalculia[tiab] OR Dyslexia*[tiab] OR Word Blindness[tiab] OR Reading Disorders[tiab] OR "Reading Disorder"[tiab] OR Alexia[tiab] OR Language Disorder*[tiab] OR Language Impairment*[tiab] OR Motor Skills Disorder*[tiab] OR Developmental Coordination Disorder*[tiab] OR Mutism*[tiab] OR Reactive Attachment Disorder[tiab] OR "Reactive Attachment Disorders"[tiab] OR "Neurotic Disorders"[Mesh] OR Neurotic Disorder*[tiab] OR Psychoneuroses[tiab] OR

Neuroses[tiab] OR "Personality Disorders"[Mesh] OR Personality Disorder*[tiab] OR Inadequate Personalit*[tiab] OR Sociopathic Personality[tiab] OR "Sociopathic Personalities"[tiab] OR Psychopathic Personality[tiab] OR Antisocial Personalit*[tiab] OR Dyssocial Behavior[tiab] OR Borderline[tiab] OR Hysteria*[tiab] OR Paranoid[tiab] OR Schizoid[tiab] OR Schizotypal[tiab] OR "Schizophrenia Spectrum and Other Psychotic Disorders"[Mesh] OR Psychosis[tiab] OR Psychotic[tiab] OR Capgras[tiab] OR delusional parasitosis[tiab] OR Psychogenic Parasitosis[tiab] OR Ekbom[tiab] OR Delusory Parasitosis[tiab] OR Morgellons[tiab] OR Paranoia*[tiab] OR Paranoid[tiab] OR Schizoaffective[tiab] OR Schizophreniform[tiab] OR Schizophrenia*[tiab] OR Schizophrenic[tiab] OR "Sleep Wake Disorders"[Mesh] OR Sleep Wake Disorder*[tiab] OR Sleep Disorder*[tiab] OR "Sleep Related Neurogenic Tachypnea"[tiab] OR Long Sleep*[tiab] OR Short Sleep*[tiab] OR Dyssomnia*[tiab] OR Sleep Deprivation*[tiab] OR Sleep Fragmentation*[tiab] OR Insufficient Sleep Syndrome[tiab] OR Circadian Rhythm Sleep Disorder*[tiab] OR Sleep Wake Cycle Disorder*[tiab] OR Advanced Sleep Phase Syndrome[tiab] OR Delayed Sleep Phase Syndrome[tiab] OR Jet Lag[tiab] OR Sleep State Misperception[tiab] OR Hypersomnia*[tiab] OR Kleine Levin[tiab] OR Paroxysmal Sleep[tiab] OR Narcoleptic[tiab] OR Gelineau[tiab] OR Narcolepsy[tiab] OR Cataleptic[tiab] OR Restless Legs[tiab] OR Disorders of Initiating and Maintaining Sleep[tiab] OR DIMS[tiab] OR Early Awakening[tiab] OR Insomnia*[tiab] OR Sleeplessness[tiab] OR Parasomnia*[tiab] OR "Sleep Drunkenness"[tiab] OR Nocturnal Paroxysmal Dystonia[tiab] OR Sleep Paralysis[tiab] OR Sleep Arousal Disorder*[tiab] OR Sleep Terror Disorder[tiab] OR Night Terror*[tiab] OR Pavor Nocturnus[tiab] OR Somnambulism[tiab] OR Sleepwalking[tiab] OR Sleep walking[tiab] OR Sleep Bruxism[tiab] OR Sleep Wake Transition Disorder*[tiab] OR Nocturnal Leg Cramp*[tiab] OR Jactatio Capitis Nocturna[tiab] OR "Trauma and Stressor Related Disorders"[Mesh] OR Reactive Disorder*[tiab] OR Adjustment Disorder*[tiab] OR Transient Situational Disturbance*[tiab] OR Stress Disorder*[tiab] OR Battered Child Syndrome[tiab] OR Combat Disorders[tiab] OR Shell Shock[tiab] OR psychological trauma*[tiab] OR "Fibromyalgia"[Mesh] OR Fibromyalgia*[tiab] OR FMS[tiab])) AND (("Probiotics"[Mesh] OR Probiotic*[tiab] OR Paraprobiotic*[tiab] OR bacterial lysate*[tiab] OR Psychobiotic*[tiab]) NOT (Synbiotic*[tiab] NOT ("Probiotics"[Mesh] OR Probiotic*[tiab] OR Paraprobiotic*[tiab] OR bacterial lysate*[tiab] OR Psychobiotic*[tiab])))) AND ("Treatment Outcome"[Mesh] OR Treatment Outcome[tiab] OR Clinical Effectiveness[tiab] OR Treatment Effectiveness[tiab] OR Treatment Efficacy[tiab] OR Clinical Efficacy[tiab] OR "Electroencephalography"[Mesh] OR EEG[tiab] OR Electroencephalogram*[tiab] OR Electroencephalograph*[tiab] OR "Magnetoencephalography"[Mesh] OR Magnetoencephalograph*[tiab] OR "Magnetic Resonance Imaging"[Mesh] OR Magnetic Resonance Imaging[tiab] OR NMR Imaging[tiab] OR NMR Tomography[tiab] OR MRI[tiab] OR Zeugmatography[tiab] OR Shift Imaging[tiab] OR "Functional MRI"[tiab] OR "Functional Magnetic Resonance Imaging"[tiab] OR ((Measur*[tiab] OR Test*[tiab]) AND (Behaviour[tiab] OR Behavior[tiab] OR Neuropsychologic*[tiab] OR Psychometric*[tiab])))) AND (("Randomized Controlled Trial"[Publication Type] OR (randomized[tiab] AND controlled[tiab] AND trial*[tiab]) OR (Placebo[tiab] AND controlled[tiab] AND trial*[tiab])) OR ("Double-Blind Method"[Mesh] OR Double Blind Method[tiab] OR Double Blind Methods[tiab] OR Double Masked Method[tiab] OR Double Masked Methods[tiab] OR Double Masked Stud*[tiab] OR Double Blind Stud*[tiab]))

Cochrane Library-

[mh "Healthy Volunteers"] OR (Healthy*):ti,ab,kw[mh "Fatigue Syndrome, Chronic"] OR ("Chronic Fatigue Syndrome*" OR CFS):ti,ab,kw OR [mh "Anxiety Disorders"] OR (Agoraphobia* OR "Separation Anxiety" OR "Neurocirculatory Asthenia" OR "Effort Syndrome" OR "Cardiac Neuros?s" OR "Hyperkinetic Heart Syndrome" OR "Neurotic Disorder*" OR Psychoneuros?s OR Neuros?s OR "Obsessive Compulsive Disorder*" OR "Anankastic Personality" OR "Hoarding Disorder*" OR "Panic Disorder*" OR "Panic Attack*" OR "Phobic Disorder*" OR "Phobic Neuros?s" OR Phobia* OR Claustrophobia* OR "Social Anxiety Disorder*"):ti,ab,kw OR [mh "Bipolar and Related Disorders"] OR ("Bipolar Disorder*" OR "Manic Depressive Psychos?s" OR "Bipolar Affective Psychos?s" OR Mania* OR "Manic State*" OR "Bipolar Depression*" OR "Manic Disorder*"):ti,ab,kw OR ([mh "Feeding and Eating Disorders"] NOT ([mh "Feeding and Eating Disorders of Childhood"] OR [mh "Pica"] OR [mh "Relative Energy Deficiency in Sport"] OR [mh "Rumination Syndrome"])) OR ("Eating Disorder*" OR "Appetite Disorder*" OR "Anorexia Nervosa" OR "Avoidant Restrictive Food Intake Disorder" OR "Food Neophobia" OR "Food Neophobic" OR ARFID OR "Binge Eating Disorder*" OR "Bulimia Nervosa" OR Diabulimia OR "Food Addiction*" OR "Night Eating Syndrome"):ti,ab,kw OR [mh "Mood Disorders"] OR ("Mood Disorder*" OR "Affective Disorder*" OR "Cyclothymic Disorder*" OR Depression* OR Depressive OR "Dysthymic Disorder*" OR "Premenstrual Dysphoric Disorder" OR "Seasonal Mood Disorder*" OR "Seasonal Affective Disorder*"):ti,ab,kw OR [mh ^"Neurocognitive Disorders"] OR ("Neurocognitive Disorder*" OR "Traumatic Psychoses"):ti,ab,kw OR [mh "Amnesia"] OR

(Amnesia*):ti,ab,kw OR ([mh "Cognition Disorders"] NOT [mh "Huntington Disease"]) OR("Cognition Disorder*" OR Overinclusion* OR "Auditory Perceptual Disorder*" OR "Auditory Processing Disorder*" OR "Auditory Comprehension Disorders" OR "Cognitive Dysfunction*" OR "Cognitive Impairment*" OR "Cognitive Decline*" OR "Mental Deterioration" OR "Postoperative Cognitive Complications" OR "Consciousness Disorder*"):ti,ab,kw OR [mh "Neurodevelopmental Disorders"] OR (("Neurodevelopmental Disorder*"):ti,ab,kw NOT ([mh "Stereotypic Movement Disorder"] OR [mh "Tic Disorders"])) OR ("Separation Anxiety" OR "Attention Deficit Disorders with Hyperactivity" OR "Attention Deficit Hyperactivity Disorder*" OR "Hyperkinetic Syndrome" OR ADDH OR "Attention Deficit Hyperactivity Disorder" OR "Attention Deficit Disorder*" OR "Minimal Brain Dysfunction" OR "Conduct Disorder*" OR "Child Behavior Disorders" OR "Autism Spectrum Disorder*" OR Asperger* OR Autism OR Autistic OR "Communication Disorder*" OR "Communicative Dysfunction" OR "Communicative Dysfunctions" OR "Communication Disabilit*" OR "Fluency Disorder*" OR "Speech Sound Disorder*" OR "Phonological Disorder*" OR "Developmental Disabilit*" OR "Intellectual Disabilit*" OR "Intellectual Development Disorder*" OR "Mental Retardation" OR Idiocy OR "Mental Deficienc*" OR "Learning Disabilit*" OR "Learning Disorder*" OR "Learning Disturbance*" OR Dyscalculia* OR Acalculia OR Dyslexia* OR "Word Blindness" OR "Reading Disorders*" OR Alexia OR "Language Disorder*" OR "Language Impairment*" OR "Motor Skills Disorder*" OR "Developmental Coordination Disorder*" OR Mutism* OR "Reactive Attachment Disorder*"):ti,ab,kw OR [mh "Neurotic Disorders"] OR ("Neurotic Disorder*" OR Psychoneuros?s OR Neuros?s):ti,ab,kw OR [mh "Personality Disorders"] OR ("Personality Disorder*" OR "Inadequate Personalit*" OR "Sociopathic Personalit*" OR "Psychopathic Personalit*" OR "Antisocial Personalit*" OR "Dyssocial Behavio?r" OR Borderline OR Hysteria* OR Schizoid OR Schizotypal):ti,ab,kw OR [mh "Schizophrenia Spectrum and Other Psychotic Disorders"] OR (Psychosis OR Psychotic OR Capgras OR "delusional parasitosis" OR "Psychogenic Parasitosis" OR Ekbom OR "Delusory Parasitosis" OR Morgellons OR Paranoia* OR Paranoid OR Schizoaffective OR Schizophreniform OR Schizophrenia* OR Schizophrenic):ti,ab,kw OR [mh "Sleep Wake Disorders"] OR ("Sleep Wake Disorder*" OR "Sleep Disorder*" OR "Sleep Related Neurogenic Tachypnea" OR "Long Sleep*" OR "Short Sleep*" OR Dyssomnia* OR "Sleep Deprivation*" OR "Sleep Fragmentation*" OR "Insufficient Sleep Syndrome" OR "Circadian Rhythm Sleep Disorder*" OR "Sleep Wake Cycle Disorder*" OR "Advanced Sleep Phase Syndrome" OR "Delayed Sleep Phase Syndrome" OR "Jet Lag" OR "Sleep State Misperception" OR Hypersomnia* OR "Kleine Levin" OR "Paroxysmal Sleep" OR Narcoleptic OR Gelineau OR Narcolepsy OR Cataleptic OR "Restless Legs" OR "Disorders of Initiating and Maintaining Sleep" OR DIMS OR "Early Awakening" OR Insomnia* OR Sleeplessness OR Parasomnia* OR "Sleep Drunkenness" OR "Nocturnal Paroxysmal Dystonia" OR "Sleep Paralysis" OR "Sleep Arousal Disorder*" OR "Sleep Terror Disorder" OR "Night Terror*" OR "Pavor Nocturnus" OR Somnambulism OR Sleepwalking OR "Sleep walking" OR "Sleep Bruxism" OR "Sleep Wake Transition Disorder*" OR "Nocturnal Leg Cramp*" OR "Jactatio Capitis Nocturna"):ti,ab,kw OR [mh "Trauma and Stressor Related Disorders"] OR ("Reactive Disorder*" OR "Adjustment Disorder*" OR Transient Situational Disturbance*" OR "Stress Disorder*" OR "Battered Child Syndrome" OR "Combat Disorders" OR "Shell Shock" OR "psychological trauma*"):ti,ab,kw OR [mh "Fibromyalgia"] OR (Fibromyalgia* OR FMS):ti,ab,kw([mh "Probiotics"] OR (Probiotic* OR Paraprobiotic* OR "bacterial lysate*" OR Psychobiotic*):ti,ab,kw) NOT ((Synbiotic*):ti,ab,kw NOT ([mh "Probiotics"] OR (Probiotic* OR Paraprobiotic* OR "bacterial lysate*" OR Psychobiotic*):ti,ab,kw)) [mh "Treatment Outcome"] OR ("Treatment Outcome" OR "Clinical Effectiveness" OR "Treatment Effectiveness" OR "Treatment Efficacy" OR "Clinical Efficacy"):ti,ab,kw OR [mh "Electroencephalography"] OR (EEG OR Electroencephalogram* OR Electroencephalograph*):ti,ab,kw OR [mh "Magnetoencephalography"] OR (Magnetoencephalograph*):ti,ab,kw OR [mh "Magnetic Resonance Imaging"] OR ("Magnetic Resonance Imaging" OR"NMR Imaging" OR "NMR Tomography" OR MRI OR Zeugmatography OR "Shift Imaging" OR "Functional MRI" OR "Functional Magnetic Resonance Imaging"):ti,ab,kw OR (((Measur* OR Test*):ti,ab,kw) AND ((Behavio?r OR Neuropsychologic* OR Psychometric*):ti,ab,kw)) (Randomized Controlled Trial):pt OR ((randomized NEAR/0 controlled NEAR/0 trial*):ti,ab,kw) OR ((Placebo NEAR/0 controlled NEAR/0 trial*):ti,ab,kw) OR [mh "Double-Blind Method"] OR ("Double Blind Method*" OR "Double Masked Method*" OR "Double Masked Stud*" OR "Double Blind Stud*"):ti,ab,kw

Web of Science-
TS=(Healthy NEAR/3 Volunteer*)TS=("Chronic Fatigue Syndrome*" OR CFS OR (Anxiety NEAR/2 Disorder*) OR Agoraphobia* OR "Separation Anxiet*" OR "Neurocirculatory Asthenia*" OR "Effort Syndrome*" OR (Cardiac NEAR/1 Neuros?s) OR "Hyperkinetic Heart Syndrome" OR (Neurotic NEAR/2 Disorder*) OR Psychoneuros?s OR Neuros?s OR "Obsessive Compulsive Disorder*" OR (Anankastic NEAR/2 Personalit*) OR (Hoarding NEAR/2 Disorder*) OR (Panic NEAR/2 (Attack* OR Disorder*)) OR (Phobic NEAR/2 (Disorder* OR Neuros?s)) OR Phobia*

OR Claustrophobia* OR “Social Anxiety Disorder*” OR (Bipolar NEAR/2 Disorder*) OR “Manic Depressive Psychos?s” OR “Bipolar Affective Psychos?s” OR Mania* OR (Manic NEAR/2 State*) OR (Bipolar NEAR/2 Depression*) OR (Manic NEAR/2 Disorder*) OR ((Feeding OR Eating OR Appetite) NEAR/2 Disorder*) OR “Anorexia Nervosa” OR “Avoidant Restrictive Food Intake Disorder” OR “Food Neophobi*” OR ARFID[tiab] OR “Binge Eating Disorder*” OR “Bulimia Nervosa” OR Diabulimia OR

(Food NEAR/2 Addiction*) OR “Night Eating Syndrome*” OR ((Mood OR Affective OR Cyclothymic OR Dysthymic OR Neurocognitive OR Cognition OR Consciousness) NEAR/2 Disorders*) OR Depression* OR Depressive OR “Premenstrual Dysphoric Disorder” OR "Seasonal Mood Disorder*" OR “Seasonal Affective Disorder*” OR (Traumatic NEAR/2 Psychos?s) OR Amnesia* OR ((Cognition NEAR/0 Disorder*) NOT "Huntington Disease") OROverinclusion* OR "Auditory Perceptual Disorder*" OR "Auditory Comprehension Disorder*" OR (Cognitive NEAR/2 (Dysfunction* OR Impairment* OR Decline)) OR (Mental NEAR/2 Deterioration) OR (Postoperative NEAR/2 Cognitive NEAR/2 Complication*) OR (“Neurodevelopmental Disorder*” NOT ("Stereotypic Movement Disorder*" OR "Tic Disorder*")) OR “Attention Deficit Hyperactivity Disorder*” OR (Hyperkinetic NEAR/2 Syndrome*) OR ADDH OR “Attention Deficit Disorder*” OR “Minimal Brain Dysfunction” OR((Conduct OR (Child NEAR/0 Behavio?r) OR (Autism NEAR/0 Spectrum)) NEAR/2 Disorder*) OR Asperger* OR Autism OR Autistic OR ((Communication OR Communicative) NEAR/2 (Disorder* OR Dysfunction* OR Diabilit*)) OR ((Fluency OR “Speech Sound” OR Phonological) NEAR/2 Disorder*) OR ((Developmental OR Intellectual) NEAR/2 Disabilit*) OR “Intellectual Development Disorder*” OR (Mental NEAR/2 Retardation*) OR Idiocy OR (Mental NEAR/2 Deficienc*) OR (Learning NEAR/2 (Disabilit* OR Disorder* OR Disturbance*)) OR Dyscalculia* OR Acalculia* OR Dyslexia* OR “Word Blindness” OR (Reading NEAR/2 Disorder*) OR Alexia* OR (Language NEAR/2 (Disorder* OR Impairment*)) OR “Motor Skills Disorder*” OR “Developmental Coordination Disorder*” OR Mutism* OR “Reactive Attachment Disorder*” OR “Neurotic Disorder*” OR Psychoneuros?es OR Neuros?s OR (Personality NEAR/2 Disorder*) OR

((Inadequate OR Sociopathic OR Psychopathic OR Antisocial) NEAR/2 Personalit*) OR “Dyssocial Behavio?r” OR Borderline OR Hysteria* OR Paranoid OR Schizoid OR Schizotypa OR Schizophrenia* OR Psychos?s OR Psychotic* OR Capgras OR ((delusional OR Delusory OR Psychogenic) NEAR/2 parasitos?s) OR Ekbom OR Morgellons OR Paranoia* OR Paranoid OR Schizoaffective OR Schizophreniform OR Schizophrenic OR “Sleep Wake Disorder*” OR “Sleep Disorder*” OR "Sleep Related Neurogenic Tachypnea" OR ((Long OR Short) NEAR/2 Sleep*) OR Dyssomnia* OR (Sleep NEAR/0 (Deprivation* OR Fragmentation*)) OR “Insufficient Sleep Syndrome” OR “Circadian Rhythm Sleep Disorder*” OR “Sleep Wake Cycle Disorder*” OR “Advanced Sleep Phase Syndrome*” OR “Delayed Sleep Phase Syndrome*” OR “Jet Lag” OR “Sleep State Misperception*” OR Hypersomnia* OR “Kleine Levin” OR (Paroxysmal NEAR/2 Sleep) OR Narcoleptic* OR Gelineau OR Narcolepsy OR Cataleptic* OR “Restless Legs” OR “Disorders of Initiating and Maintaining Sleep” OR DIMS OR “Early Awakening” OR Insomnia* OR Sleeplessness OR Parasomnia* OR "Sleep Drunkenness" OR “Nocturnal Paroxysmal Dystonia*” OR (Sleep NEAR/0 (Paralysis OR (Arousal NEAR/0 Disorder*) OR (Terror NEAR/0 Disorder*) OR Walking OR Bruxism)) OR “Night Terror*” OR “Pavor Nocturnus” OR Somnambulism OR Sleepwalking OR “Sleep Wake Transition Disorder*” OR “Nocturnal Leg Cramp*” OR “Jactatio Capitis Nocturna” OR "Trauma and Stressor Related Disorder*" OR ((Reactive OR Trauma OR Adjustment OR Stress OR Combat) NEAR/2 Disorder*) OR “Transient Situational Disturbance*” OR “Battered Child Syndrome” OR “Shell Shock” OR “psychological trauma*” OR Fibromyalgia* OR FMS) TS=((Probiotic* OR Paraprobiotic* OR “bacterial lysate*” OR Psychobiotic*) NOT (Synbiotic* NOT (Probiotic* OR Paraprobiotic* OR “bacterial lysate*” OR Psychobiotic*)))TS=("Treatment Outcome" OR ((Clinical OR Treatment) NEAR/2 (Effectiveness OR Efficacy)) OR Electroencephalography OR EEG OR Electroencephalogram* OR Electroencephalograph* OR Magnetoencephalograph* OR “Magnetic Resonance Imaging” OR “NMR Imaging” OR “NMR Tomography” OR MRI OR Zeugmatography OR “Shift Imaging*” OR “Functional Magnetic Resonance Imaging” OR ((Measur* OR Test*) NEAR/5 (Behavio?r OR Neuropsychologic* OR Psychometric*)))TS=("Randomized Controlled Trial" OR (randomized NEAR/2 controlled NEAR/2 trial*) OR (Placebo NEAR/2 controlled NEAR/2 trial*) OR “Double Blind Method*” OR “Double Masked Method*” OR “Double Masked Stud*” OR “Double Blind Stud*”)

Table S1. Bacterial Species of Probiotics.

Article	Name of the product or manufacturer	Species
**Akkasheh et al. (2016)**	Tak Gen Zist Pharmaceutical Company	*Lactobacillus acidophilus*<br>*Lactobacillus casei*<br>*Bifidobacterium bifidum*
**Adikari et al. (2020)**	commercially available by the Ministry of Health, Malaysia	*Lactobacillus Casei Shirota*
**Allen et al. (2016)**	Alimentary Health	*Bifidobacterium longum 1714*
**Bagga et al. (2018)**	Ecologic 825 by Winclove Probiotics	*Lactobacillus casei W56*<br>*Lactobacillus acidophilus W22*<br>*Lactobacillus paracasei W20*<br>*Bifidobacterium lactis W51*<br>*Lactobacillus salivarius W24*<br>*Lactobacillus lactis W19*<br>*Bifidobacterium lactis W52*<br>*Lactobacillus plantarum W62*<br>*Bifidobacterium bifidum W23*
**Bagga et al. (2019)**	Ecologic 825 by Winclove Probiotics	*Lactobacillus casei W56*<br>*Lactobacillus acidophilus W22*<br>*Lactobacillus paracasei W20*<br>*Bifidobacterium lactis W51*<br>*Lactobacillus salivarius W24*<br>*Lactobacillus lactis W19*<br>*Bifidobacterium lactis W52*<br>*Lactobacillus plantarum W62*<br>*Bifidobacterium bifidum W23*
**Benton et al. (2007)**	Yakult	*Lactobacillus casei Shirota*
**Chung et al. (2014)**	sponsored by ILDONG Pharmaceutical Co., Ltd.	*Lactobacillus helveticus IDCC3801*
**De Lorenzo et al. (2017)**	Biocult Strong, HOMEOSYN	*Streptococcus thermophilus SGSt01*<br>*Bifidobacterium animalis subsp. Lactis SGB06*<br>*Streptococcus thermophiles*<br>*Bifidobacterium bifidum SGB02*<br>*Lactobacillus delbrueckii spp. Bulgaricus DSM 20081*<br>*Lactococcus lactis subsp. Lactis SGLc01*<br>*Lactobacillus acidophilus SGL11*<br>*Lactobacillus plantarum SGL07*<br>*Lactobacillus reuteri SGL01*
**Dickerson et al. (2014)**	Bifiform Balance	*Lactobacillus rhamnosus strain GG*<br>*Bifidobacterium animalis subsp. lactis Bb12*
**Dickerson et al. (2018)**	Chr. Hansen	*Lactobacillus GG*<br>*Bifidobacterium lactis strain Bb12*
**Diop et al. (2008)**	Probio Stick	*Lactobacillus acidophilus Rosell - 52*<br>*Bifidobacterium longum Rosell - 175*
**Eskandarzadeh et al. (2021)**	Takgene Zist Company	*Bifidobacterium longum*<br>*Bifidobacterium bifidum*

		Bifidobacterium lactis<br>*Lactobacillus acidophilus bacteria*
**Ghaderi et al. (2019)**	Lacto Care	*Lactobacillus acidophilus*<br>*Bifidobacterium bifidum*<br>*Lactobacillus reuteri*<br>*Lactobacillus fermentum*
**Gualtieri et al. (2020)**	Biocult Strong, HOMEOSYN	*Streptococcus thermophiles (CNCM strain number I-1630)*<br>*Bifidobacterium animalis subsp. Lactis*<br>*Bifidobacterium bifidum*<br>*Streptococcus thermophiles*<br>*Lactobacillus bulgaricus*<br>*Lactococcus lactis subsp. lactis*<br>*Lactobacillus acidophilus*<br>*Lactobacillus plantarum*<br>*Lactobacillus reuteri*
**Ho et al. (2021)**	PS128 by Bened Biomedical Co. Ltd. (Taipei, Taiwan)	*Lactobacillus plantarum PS128*
**Hwang et al. (2019)**	CHEBIGEN Inc.	*DW2009 Lactobacillus plantarum C29*
**Inoue et al. (2018)**	Morinaga Milk Industry Co., Ltd.	*B. longum BB536*<br>*B. infantis M-53*<br>*B. breve M-16V*<br>*B. breve B-3*
**Karbownik et al. (2020)**	LacidoEnter<br>Dicoflor 60	*Saccharomyces boulardii CNCM I - 1079*<br>*Lactobacillus rhamnosus GG (ATCC 53103*
**Kato-Kataoka et al. (2016)**	Yakult Central Institute	*Lactobacillus casei strain Shirota YIT 9029*
**Kazemi et al. (2019)**	Lallemand Health Solution	*Lactobacillus helveticus R0052*<br>*Bifidobacterium longum R0175*
**Kelly et al. (2017)**	Alimentary Health Ltd.	*Lactobacillus rhamnosus (JB-1)*
**Kobayashi et al. (2019)**	Morinaga Milk Industry Co., Ltd.	*Bifidobacterium breve A1*
**Lew et al. (2019)**	JinHua YinHe Biological Technology Co. Ltd.	*Lactobacillus plantarum P8*
**Messaoudi et al. (2011)**	Institut Rosell-Lallemand	*Lactobacillus helveticus R0052*<br>*Bifidobacterium longum R0175*
**Mohammadi et al. (2016)**	yogurt: Pegah Company<br>capsules: ZistTakhmir Co	*yogurt: with a total of min 1x10^7*<br>*Lactobacillus acidophilus LA5*<br>*Bifidobacterium lactis BB12*<br>*capsules:*<br>*Actobacillus casei 3x10^3 CFU/g*<br>*Lactobacillus acidophilus 3x10^7 CFU/g*<br>*Lactobacillus rhamnosus 7x10^9 CFU/g*<br>*Lactobacillus bulgaricus 5x10^8 CFU/g*<br>*Bifidobacterium breve 2x10^10 CFU/g*<br>*Bifidobacterium longum 1x10^9 CFU/g*<br>*Streptococcus thermophilus 3x10^8 CFU/g*
**Murata et al. (2018)**	LAC - Shield by Morinaga Milk Industry	*Lactobacillus paracasei MCC1849*
**Nishida et al. (2017)**	Correspondence Research & Development Center, Asahi Group Holdings, Ltd.	*Lactobacillus gasseri CP2305*
**Nishida et al. (2019)**	funded by Asahi Group Holdings, Ltd.	*Lactobacillus gasseri CP2305*

**Nishihira et al. (2014)**	MegMilk Snow Brand Co. Ltd.	*probiotic group:*<br>*Lactobacillus gasseri SBT2055*<br>*Bifidobacterium longum SBT2928*<br>*placebo group:*<br>*Streptococcus thermophilus*<br>*Lactobacillus delbrueckii subsp. bulgaricus*
**Ohsawa et al. (2018)**	funded by Asahi Group Holdings, Ltd.	*Lactobacillus helveticus CM4*
**Östlund-Lagerström et al. (2016)**	BioGaia AB	*Lactobacillus reuteri DSM 17938*
**Papalini et al. (2019)**	Ecologic Barrier	*Bifidobacterium bifidum W23*<br>*Bifidobacterium lactis W51*<br>*Bifidobacterium lactis W52*<br>*Lactobacillus acidophilus W37*<br>*Lactobacillus brevis W63*<br>*Lactobacillus casei W56*<br>*Lactobacillus salivarius W24*<br>*Lactococcus lactis W19*<br>*Lactococcus lactis W58*
**Patterson et al. (2020)**	DuPont Nutrition & Biosciences	*Lacticaseibacillus paracasei Lpc-37*
**Rao et al. (2009)**	Yakult	*Lactobacillus casei strain Shirota*
**Raygan et al. (2018)**	LactoCare	*Lactobacillus acidophilus*<br>*Lactobacillus reuteri*<br>*Lactobacillus fermentum*<br>*Bifidobacterium bifidum*
**Raygan et al. (2019)**	LactoCare	*Lactobacillus acidophilus*<br>*Lactobacillus reuteri*<br>*Lactobacillus fermentum*<br>*Bifidobacterium bifidum*
**Reininghaus (2020)**	OMNi-BiOTiC Stress Repair	*B. bifidum W23*<br>*B. lactis W51*<br>*B. lactis W52*<br>*L. acidophilus W22*<br>*L. casei W56*<br>*L. paracasei W20*<br>*L. plantarum W62*<br>*L. salivarius W24*<br>*L. lactis W19*
**Roman et al. (2018)**	ERGYPHILUS Plus	*Lactobacillus rhamnosus GG*<br>*Lactobacillus casei*<br>*Lactobacillus acidophilus*<br>*Bifidobacterium bifius*
**Romijn et al. (2017)**	Lallemand Health Solution	*Lactobacillus helveticus R0052 (CNCM strain I-1722)*<br>*Bifidobacterium longum R0175 (CNCM strain I-3470)*
**Rudzki et al. (2019)**	Sanprobi IBS by Probi AB	*Lactobacillus plantarum 299v*
**Sanchez et al. (2017)**	sponsored by the Nestlé Research Center	*Lactobacillus rhamnosus CGMCC1.3724*
**Sashihara et al. (2013)**	Meiji Co. Ltd.	*Lactobacillus gasseri OLL2809*

**Sawada et al. (2017)**	Asahi Group Holdings, Ltd.	*Lactobacillus gasseri CP2305*
**Severance et al. (2017)**	Bifiform Balance	*Lactobacillus rhamnosus strain GG*<br>*Bifidobacterium animalis subsp. lactis Bb12*
**Shinkai et al. (2013)**	Faculty of applied Bioscience, Tokyo University of Agriculture providing lactic acid bacteria, study conducted under the contract of the Tokyo Metropolitan Institute of Gerontology (TMIG) and Otsuka Pharmaceutical Company Limited	*Lactobacillus pentosus strain b240*
**Steenbergen et al. (2015)**	Ecologic Barrier by Winclove	*Bifidobacterium bifidum W23*<br>*Bifidobacterium lactis W52*<br>*Lactobacillus acidophilus W37*<br>*Lactobacillus brevis W63*<br>*Lactobacillus casei W56*<br>*Lactobacillus salivarius W24*<br>*Lactococcus lactis (W19 and W58)*
**Takada et al. (2016)**	Yakult Central Institute	*Lactobacillus casei YIT 9029*
**Takada et al. (2017)**	Yakult Central Institute	*Lactobacillus casei strain Shirota YIT 9029*
**Tillisch et al. (2013)**	Danone Research facilities	*Bifidobacterium animalis subsp lactis CNCM strain number I-2494 -> 1,25x10^10 CUF/cup Streptococcus thermophilus CNCM strain number I-1630 -> 1,2x10^9 CFU/cup Lactobacillus bulgaricus CNCM strain numbers I-1632 and I-1519 -> 1,2x10^9 CFU/cup Lactococcus lactis subsp lactis CNCM strain number I-1631*
**Tran et al. (2019)**	"commercially available as over - the - counter products (e.g., Amazon)"	*Bifidobacterium:*<br>*B. lactis*<br>*B. bifidum*<br>*B. breve*<br>*B. longum*<br>*B. infantis*<br>*B. adolescentis*<br>*Lactobacillus:*<br>*L. acidophilus*<br>*L. brevis*<br>*L. bulgaricus*<br>*L. casei*<br>*L. paracasei*<br>*L. plantarum*<br>*L. salivarius*<br>*L. rhamnosus*<br>*L. fermentum*<br>*L. gasseri*<br>*L. helveticus*<br>*L. reuteri*<br>*L. crispatus*<br>*Lactococcus lactis*

		Streptococcus thermophilus<br>*Bacillus coagulans*
**Wang et al. (2019)**	Alimentary Health Group	*Bifidobacterium longum 1714*
**Xiao et al. (2020)**	Morinaga Culture Collection, Zama, Japan	*Bifidobacterium breve A1 MCC1274*
**Yamamura et al. (2009)**	NR	*Lactobacillus helveticus strain CM4*
**Zhang et al. (2021)**	manufactured by Yakult Corporation, Shanghai, China	*Lacticaseibacillus paracasei YIT 9029 (strain Shirota: LcS)*

NR: not reported; CFU: Colony forming unit

Table S2. Questionnaires and Tests applied by the included studies.

Abbreviation	Description
ACQ-R	Anxiety control questionnaire-revised
ADS	Allgemeine Depressionsskala
APL	Everyday Problem List
APT	Attention and Perceptivity Test
BAI	Beck Anxiety Inventory
BAS	Behavioural Approach Scale
BDI	Beck Depression Inventory score
BDI II	Beck Depression Inventory score II
BIS	Behavioural Inhibition Scale
BPRS	Brief Psychiatric Rating Scale
BUT	Body Uneasiness Test
CANTAB	Cambridge Neuropsychological Test Automated Battery
CBB	CogState Brief Battery
CBG	Cyberball stress - social stress
CCL	Coping Checklist
CVLT	California Verbal Learning Test
DASS-42	Depression, Anxiety and Stress Scale questionnaire
DST	Digiti Span Test
DVT	Digit Vigilance Test
EAT - 26	Eating Attitudes Test - 26
ECG	Electrocardiogram
EDI-2	Eating Disorder Inventory 2
EDR	Electrodermal Responses
EEG	Electroencephalogram
EMG	Electromyogram
EOG	Electrooculogram
EPDS	Edinburgh Postnatal Depression Scale
ESS	Epworth Sleepiness Scale
GAD-7	Generalised Anxiety Disorder - 7 (anxiety score)
GAF	Global Assessment of Functioning
GDS - SF	geriatric depression scale - short form
GHQ-28	28item General Health Questionnaire
GLQI	Gastrointestinal Quality of life questionnaire
GSI	Global Severity Index

HADS	Hospital Anxiety and Depression Scale
HAM-A	Hamilton Anxiety Rating Scale
HAM-D 17	Hamilton Depression Rating
HDI	Henry Ford Hospital Headache Disability Inventory
HSCL-90	Hopkins Symptom Checklist
IBS - SSS	Irritable Bowel Syndrome Severity Scale
ICGI	Improved Clinical Global Impressions scale
IGT	Iowa Gambling Task
ISI	Insomnia Severity Index
JMCIS	Japanese version of the MCI Screen
LEIDS	Leiden index of depression severity
MAAS	Maternal Antenatal Attachment Scale
MADRS	Montgomery-Åsberg Depression Rating Scale
MIDAS	Migraine Disability Assessment Scale
MMSE	Mini - Mental State Examination
MoCA-J	Japanese version of the Montreal Cognitive Assessment instrument
MPAS	Maternal Postnatal Attachment Scale
MQ	Mood Questionnaire
MSS	Mania Self Rating Scale
NART	National Adult Reading Test
NEO - FFI	NEO - Five - Factor Inventory
NMR	Negative mood regulation
NTS	Need Threat Scale
OSA	Oguri-Shirakawa-Azumi sleep inventory
PAL	Paired Associate Learning test
PANAS	Positive And Negative Affect Schedule
PES	Pregnancy Experience Scale
PHQ-9	Patient Health Questionnaire -9 (depression score)
POMS	Profile of Mood States
PRAQ - R	Pregnancy related Anxiety Questionnaires - Revised
PSDI	Positive Symptom Distress Index
PSQI	Pittsburgh Sleep Quality Index
PSS	Perceived Stress Scale
PST	Positive Symptom Total
PSWQ	Penn state worry questionnaire
QIDS-SR16	Quick Inventory of Depressive Symptomatology
RBANS	Repeatable Battery for the Assessment of Neuropsychological Status
RFFT	Ruff Figural Fluency Test
RVIP	Rapid Visual Information - Processing Task
SCID - I	Semi - Structured - Interview
SCL-90	Symptoms Checklist-90
SCL90R	Symptom Checklist - Revised
SECPT	Socially Evaluated Cold Pressure Test
SEP	Subjective "Exclusion Perception"
SF-36	Short Form Health Survey
SHRI	Sleep Health Risk Index
SRT	Story Recall Test
ST	Stroop Colour - Word Test
STAI	State Trait Anxiety Inventory
TICS	Trier Inventory for Chronic Stress
TMT	Trail Making Test parts A and B

TSST	Trier Social Stress Test
VLT	Verbal Learning Test
WHO - QOL - BREF	WHO - Quality of Life Questionnaire
YMRS	Young Mania Rating Scale
Zung - SDS	Zung Self-rating Depression Scale

Table S3. Study characteristics across studies.

		**all trials (n=54)**	**trials in meta-analysis (n=30)**
**Sample size**			
**Sample randomized**			
	Sum	4449	3017
	Mean	82.4	100.6
	Median	63	77
	Minimum	20	39
	Maximum	300	300
	IQR	40.8-96.5	50.5-117.5
**Sample at the end of trial**			
	Sum	3700	2563
	Mean	78.7	91.5
	Median	60	64.5
	Minimum	19	31
	Maximum	278	278
	IQR	42-93	52.3-101
**Sample analysed**			
	Sum	4130	2595
	Mean	76.5	96.1
	Median	60	69
	Minimum	19	31
	Maximum	294	294
	IQR	40-93.5	50.5-113.5
**NR**		7	2
**No dropouts/NA**		6	4
**Age**			
	Mean (SD)	42.7 (20)	42.2 (20.6)
	Median	35.6	36.2
	IQR	24.9-50.1	22.8-51.4
	Minimum	18	18
	Maximum	98	98
	NR	3	0
**Sex**			
**Female**			
	Number	2473	1723
	Percentage	60.1%	62.9%
	Trials only female	4	2
**Male**			
	Number	1641	1017

	Percentage	39.9%	37.1%
	Trials only male	5	1
**Continent**			
	Europe (n; %)	18; 33.3%	10; 33.3%
	Asia (n; %)	28; 51.9%	18; 60%
	America (n; %)	7; 12%	2; 6,7%
	Oceania (n; %)	1; 1.9%	0
**Country**			
	Japan (n; %)	15; 27.8%	9; 30%
	Iran (n; %)	7; 13.5%	6; 20%
	USA (n; %)	5; 9.6%	0
**Date (Years)**			
	Range	2007 - 2021	2011- 2021
	Most (%)	2019 (22.2%)	2016, 2019, 2020 (each 16.7%)
**Intake Time (Weeks)**			
	Total	484	279
	Mean	9	9.3
	Median	8	8
	IQR	4-12	4.3-12
	Range	3 - 24	4 – 24
	Most (%)	4, 8 (each 24.1%)	4, 8 (26.7%)
**Strain numbers**			
	Total (Strains)	167	105
	Mean	3.1	3.5
	Median	1	1
	Minimum	1	1
	Maximum	20	20
	IQR	1-4	1-4
	Most (%)	1 (53.7%)	1 (53.3%)
**CFU**			
	Total	9.9×10^{11}	5.9×10^{11}
	Mean	2×10^{10}	2.3×10^{10}
	Median	1×10^{10}	9×10^{9}
	Min	2.3×10^{7}	2.4×10^{7}
	Max	1.4×10^{11}	1.4×10^{11}
	IQR	2.5×10^{9}-2.1×10^{10}	3.5×10^{9}-2×10^{10}
	NR	5 (9.3%)	4 (13.3%)
**Application**			
	Capsules (n; %)	13; 24.1%	6; 26.7%
	Tablets/pills (n; %)	7; 13%	5; 16.7%
	Liquids/Yoghurt (n; %)	12; 22.2%	7; 23.3%
	Powder/Sachet/Sticks (n; %)	19; 35.2%	8; 26.7%
	NR (n; %)	3; 5.6%	2; 6.7%
**Daily Intake**			
	1/day (n; %)	46; 85.2%	24; 80%
	2/day (n; %)	3; 5.6%	3; 10%

3/day (n; %)	3; 5.6%	1; 3.3%
4/day (n; %)	2; 3.7%	2; 6.7%

n: number; SD: standard deviation; IQR: interquartile range; NR: not reported; CFU: colony forming unit

Table S4. Overview of the most used questionnaires and their results of all included studies.

Author	BAI	BDI/BDI-II	DASS-42	GHQ	HADS-A	HADS-D	HAM-A	HAM-D	LEIDs	PANSS	POMS	PSQI	PSS	RBANS	SCL90	STAI state	STAI trait
Akkasheh et al. (2016)	NR	↓	NR	NR	NR	NR	NR	NR	NR	NR	NR	NR	NR	NR	NR	NR	NR
Adikari et al. (2020)	NR	NR	NR	NR	NR	NR	NR	NR	NR	NR	NR	NR	NR	NR	NR	NR	NR
Allen et al. (2016)	NR	NR	NR	NR	NR	NR	NR	NR	NR	NR	NR	NR	↓	NR	NR	↔	NR
Bagga et al. (2018)	NR	NR	NR	NR	NR	NR	NR	NR	NR	NR	NR	NR	NR	NR	↔	NR	NR
Bagga et al. (2019)	NR	NR	NR	NR	NR	NR	NR	NR	NR	NR	NR	NR	NR	NR	NR	NR	NR
Benton et al. (2007)	NR	NR	NR	NR	NR	NR	NR	NR	NR	NR	↔	NR	NR	NR	NR	NR	NR
Chung et al. (2014)	NR	NR	NR	NR	NR	NR	NR	NR	NR	NR	NR	NR	↔	NR	NR	NR	NR
De Lorenzo et al. (2017)	NR	NR	NR	NR	NR	NR	NR	NR	NR	NR	NR	NR	NR	NR	↔	NR	NR
Dickerson et al. (2014)	NR	NR	NR	NR	NR	NR	NR	NR	NR	↔	NR	NR	NR	NR	NR	NR	NR
Dickerson et al. (2018)	NR	NR	NR	NR	NR	NR	NR	NR	NR	NR	NR	NR	NR	NR	NR	NR	NR
Diop et al. (2008)	NR	NR	NR	NR	NR	NR	NR	NR	NR	NR	NR	NR	NR	NR	NR	NR	NR

Eskandarzadeh et al. (2021)	↔	NR	NR	NR	NR	NR	↓↓	NR	NR	NR	NR	NR	NR	NR	NR	↓	↔
Ghaderi et al. (2019)	NR	NR	NR	NR	NR	NR	NR	NR	NR	↓↓	NR	NR	NR	NR	NR	NR	NR
Gualtieri et al. (2020)	NR	NR	NR	NR	NR	NR	↓↓	NR	NR	NR	NR	NR	NR	NR	↔	NR	NR
Ho et al. (2021)	↔	↓	NR	NR	NR	NR	NR	NR	NR	NR	NR	↔	NR	NR	NR	NR	NR
Hwang et al. (2019)	NR	NR	NR	NR	NR	NR	NR	NR	NR	NR	NR	NR	NR	NR	NR	NR	NR
Inoue et al. (2018)	NR	NR	NR	NR	NR	NR	NR	NR	NR	NR	NR	NR	NR	NR	NR	NR	NR
Karbownik et al. (2020)	NR	NR	NR	NR	NR	NR	NR	NR	NR	NR	NR	NR	NR	NR	NR	↔	↔
Kato-Kataoka et al. (2016)	NR	NR	NR	NR	NR	NR	NR	NR	NR	NR	NR	NR	NR	NR	NR	↔	↔
Kazemi et al. (2019)	NR	↓	NR	NR	NR	NR	NR	NR	NR	NR	NR	NR	NR	NR	NR	NR	NR
Kelly et al. (2017)	↔	↔	NR	NR	NR	NR	NR	NR	NR	NR	NR	↔	↔	NR	↔	↔	↔
Kobayashi et al. (2019)	NR	NR	NR	NR	NR	NR	NR	NR	NR	NR	NR	NR	NR	↔	NR	NR	NR
Lew et al. (2019)	NR	NR	↓	NR	NR	NR	NR	NR	NR	NR	NR	NR	↔	NR	NR	NR	NR
Messaoudi et al. (2011)	NR	NR	NR	NR	↔	↔	NR	NR	NR	NR	NR	NR	↔	NR	↓	NR	NR

Mohammadi et al. (2016)	NR	NR	↔	↔	NR	NR	NR	NR	NR	NR	NR	NR	NR	NR	NR	NR	NR
Murata et al. (2018)	NR	NR	NR	NR	NR	NR	NR	NR	NR	NR	↔	NR	NR	NR	NR	NR	NR
Nishida et al. (2017)	NR	NR	NR	↔	↔	↔	NR	NR	NR	NR	NR	↔	NR	NR	NR	↔	↔
Nishida et al. (2019)	NR	NR	NR	↔	NR	↔	↔	NR	NR	NR	NR	↓	NR	NR	NR	↔	↓
Nishihira et al. (2014)	NR	NR	NR	↔	NR	NR	NR	NR	NR	NR	NR	NR	NR	NR	NR	NR	NR
Ohsawa et al. (2018)	NR	NR	NR	NR	NR	NR	NR	NR	NR	NR	↔	NR	NR	↔	NR	NR	NR
Östlund-Lagerström et al. (2016)	NR	NR	NR	NR	↔	↔	NR	NR	NR	NR	NR	NR	↔	NR	NR	NR	NR
Papalini et al. (2019)	NR	↔	NR	NR	NR	NR	NR	NR	↔	NR	NR	NR	NR	NR	NR	NR	NR
Patterson et al. (2020)	↔	NR	↔	NR	NR	NR	NR	NR	NR	NR	NR	NR	↔	NR	NR	↔	↔
Rao et al. (2009)	↓	↔	NR	NR	NR	NR	NR	NR	NR	NR	NR	NR	NR	NR	NR	NR	NR
Raygan et al. (2018)	↓↓	↓↓	NR	↓↓	NR	NR	NR	NR	NR	NR	NR	NR	NR	NR	NR	NR	NR
Raygan et al. (2019)	↓	↓↓	NR	NR	NR	NR	NR	NR	NR	NR	NR	↔	NR	NR	NR	NR	NR

Reininghaus et al.(2020)	NR	↔	NR	NR	NR	NR	NR	↔	NR	NR	NR	NR	NR	NR	↔	NR	NR
Roman et al. (2018)	NR	↔	NR	NR	NR	NR	NR	NR	NR	NR	NR	NR	NR	NR	NR	↔	↔
Romijn et al. (2017)	NR	NR	↔	NR	NR	NR	NR	NR	NR	NR	NR	NR	NR	NR	NR	NR	NR
Rudzki et al. (2019)	NR	NR	NR	NR	NR	NR	NR	↔	NR	NR	NR	NR	↔	NR	↔	NR	NR
Sanchez et al. (2017)	NR	↓	NR	NR	NR	NR	NR	NR	NR	NR	NR	NR	↔	NR	NR	↔	↔
Sashihara et al. (2013)	NR	NR	NR	NR	NR	NR	NR	NR	NR	NR	NR	NR	NR	NR	NR	NR	NR
Sawada et al. (2017)	NR	NR	NR	↔	↓	↓	NR	NR	NR	NR	NR	↓	NR	NR	NR	↓	NR
Severance et al. (2017)	NR	NR	NR	NR	NR	NR	NR	NR	NR	↔	NR	NR	NR	NR	NR	NR	NR
Shinkai et al. (2013)	NR	NR	NR	NR	NR	NR	NR	NR	NR	NR	↔	NR	NR	NR	NR	NR	NR
Steenbergen et al. (2015)	↔	↔	NR	NR	NR	NR	NR	NR	↓↓↓	NR	NR	NR	NR	NR	NR	NR	NR
Takada et al. (2016)	NR	NR	NR	NR	NR	NR	NR	NR	NR	NR	NR	NR	NR	NR	NR	↔	↔
Takada et al. (2017)	NR	NR	NR	↔	NR	NR	NR	NR	NR	NR	NR	↔	NR	NR	NR	↔	↔
Tillisch et al. (2013)	NR	NR	NR	NR	↔	↔	NR	NR	NR	NR	NR	NR	NR	NR	NR	NR	NR

Tran et al. (2019)	↔	NR	NR	NR	NR	NR	NR	NR	NR	NR	NR	NR	NR	NR	NR	NR	NR
Wang et al. (2019)	NR	NR	NR	NR	NR	NR	NR	NR	NR	NR	NR	NR	NR	NR	NR	NR	NR
Xiao et al. (2020)	NR	NR	NR	NR	NR	NR	NR	NR	NR	NR	NR	NR	NR	↑↑↑	NR	NR	NR
Yamamura et al. (2009)	NR	NR	NR	NR	NR	NR	NR	NR	NR	NR	NR	NR	NR	NR	NR	NR	NR
Zhang et al. (2021)	NR	NR	NR	NR	NR	NR	NR	NR	NR	NR	NR	NR	NR	NR	NR	NR	NR

A summary of the raw data as direction of change of the applied questionnaires are presented. It is questionnaire specific whether or not a decrease implies improvement or deterioration and accordingly, whether or not an increase implies improvement or deterioration.↔: no significant differences between the groups; ↓: significant decrease $p<0.05$; ↓↓: significant decrease $p<0.01$; ↓↓↓: significant decrease $p<0.001$; ↑: significant increase $p<0.05$; ↑↑: significant increase $p<0.01$; ↑↑↑: significant increase $p<0.001$; NR: not reported; BAI: Beck Anxiety Inventory; BDI: Beck Depression Inventory; DASS-42: Depression, Anxiety and Stress Scale ; GHQ: General Health Questionnaire ; HADS-A/-D: Hospital Anxiety and Depression Scale; HAM-A: Hamilton Anxiety Rating Scale; HAM-D: Hamilton Depression Rating Scale ; LEIDs: Leiden index of depression severity; POMS: Profile of Mood States; PSQI: Pittsburgh Sleep Quality Index; PSS: Perceived Stress Scale; RBANS: Repeatable Battery for the Assessment of Neuropsychological Status; SCL90: Symptoms Checklist-90; STAI: State Trait Anxiety Inventory

Table S5. Study Outcomes at qualitative level for the most used questionnaires of the studies included in the meta-analysis.

Author	Year	Anxiety					Depression			Psychiatric Distress		Mood	Stress
		STAI state	STAI trait	BAI	HADS-A	HAM-A	BDI	HADS-D	HAM-D	GHQ (total)	SCL-90 (GSI)	POMS	PSS
**Akkasheh et al.**	2016						↓						
**Chung, Y.C. et al.**	2014												↔
**Eskandarzadeh et al.**	2021	↓	↔	↔		↓↓							
**Gualtieri et al.**	2021					↓↓					↔		
**Ho et al.**	2021			↔			↓						
**Karbownik et al.**	2020	↔	↔										
**Kato-Kataoka et al.**	2016	↔	↔										
**Kazemi et al.**	2020						↓						
**Messaoudi et al.**	2011				↔			↔			↓		↔
**Mohammadi et al.**	2016									↔			
**Murata, M. et al.**	2018											↔	
**Nishida et al.**	2019	↔	↓		↔			↔		↔			
**Nishihira et al.**	2014									↔			
**Ohsawa, K. et al.**	2018											↔	
**Östlund-Lagerström et al.**	2016				↔			↔					↔
**Papalini et al.**	2019						↔						
**Patterson et al.**	2020	↔		↔									↔
**Raygan et al.**	2019			↓			↓↓						
**Raygan et al.**	2018			↓↓			↓↓			↓↓			
**Reininghaus et al.**	2020						↔		↔		↔		
**Roman et al.**	2018	↔	↔				↔						
**Rudzki et al.**	2019								↔		↔		
**Sanchez et al.**	2017	↔	↔				↓						↔
**Sashihara, T. et al.**	2013											↔	
**Shinkai et al.**	2013											↔	
**Steenbergen et al.**	2015			↔			↔						
**Takada et al.**	2016	↔	↔										
**Takada et al.**	2017	↔	↔							↔			
**Tran et al.**	2019			↔									
**Zhang et al.**	2021						↔		↔				

A summary of the raw data as direction of change of the applied questionnaires are presented. It is questionnaire specific whether or not a decrease implies improvement or deterioration and accordingly, whether or not an increase implies improvement or deterioration.

↔: no significant differences between the groups; ↓: significant decrease p<0.05; ↓↓: significant decrease p<0.01; ↓↓↓: significant decrease p<0.001; BAI: Beck Anxiety Inventory; BDI: Beck Depression Inventory; GHQ: General Health Questionnaire ; HADS-A/-

D: Hospital Anxiety and Depression Scale; HAM-A: Hamilton Anxiety Rating Scale; HAM-D: Hamilton Depression Rating Scale; POMS: Profile of Mood States; PSS: Perceived Stress Scale; SCL90: Symptoms Checklist-90; STAI: State Trait Anxiety Inventor

Figure S1. Effect of intake period on Anxiety of randomized controlled trials receiving either probiotics or placebo treatment.

Study or Subgroup	Probiotics Mean	SD	Total	Placebo Mean	SD	Total	Weight	Std. Mean Difference IV, Random, 95% CI
**2.1.1 4-8 weeks**								
Eskandarzadeh 2021 [30]	-15.94	6.78	18	-7.08	7.43	21	5.3%	-1.22 [-1.91, -0.52]
Ho 2021 [55]	-4.1	5.42	21	-2.37	5.05	19	5.5%	-0.32 [-0.95, 0.30]
Karbownik 2020 [64]	16.2	14.18	31	16.9	9.33	29	6.0%	-0.06 [-0.56, 0.45]
Kato-Kataoka 2016 [37]	8.95	6.81	23	8.92	7.95	24	5.8%	0.00 [-0.57, 0.58]
Messaoudi 2011 [66]	-2	1.63	26	7	2.27	29	4.0%	-4.45 [-5.46, -3.44]
Patterson 2020 [68]	1.87	5.79	58	1.03	5.91	59	6.6%	0.14 [-0.22, 0.51]
Roman 2018 [70]	1.75	9.5	16	4.26	12.28	15	5.2%	-0.22 [-0.93, 0.48]
Steenbergen 2015 [72]	-1.4	5.44	20	-1	5.57	20	5.6%	-0.07 [-0.69, 0.55]
Takada 2016 [52]	12.03	10.96	70	11.31	9.36	70	6.7%	0.07 [-0.26, 0.40]
Tran 2019 [77]	-0.88	5.91	55	-2.67	7.8	9	5.2%	0.29 [-0.42, 0.99]
**Subtotal (95% CI)**			**338**			**295**	**55.9%**	**-0.50 [-1.03, 0.04]**
Heterogeneity: Tau² = 0.65; Chi² = 84.81, df = 9 (P < 0.00001); I² = 89%								
Test for overall effect: Z = 1.82 (P = 0.07)								
**2.1.2 9-24 weeks**								
Gualtieri 2020 [63]	-2.99	5.46	65	-0.37	4.3	34	6.4%	-0.51 [-0.93, -0.09]
Nishida 2019 [44]	8.8	1.22	31	7.6	1.5	29	5.9%	0.87 [0.34, 1.40]
Östlund-Lagerström 2016 [67]	-0.22	2.7	125	0.06	3	124	6.9%	-0.10 [-0.35, 0.15]
Raygan 2018 [48]	-2.1	2.3	30	-0.8	1.4	30	6.0%	-0.67 [-1.20, -0.15]
Raygan 2019 [47]	-2.4	3.38	27	-1.1	3.39	27	5.9%	-0.38 [-0.92, 0.16]
Sanchez 2017 [78]	-0.6	8.1	62	1.5	8.9	63	6.6%	-0.25 [-0.60, 0.11]
Takada 2017 [51]	17.33	10	50	13.96	11.27	48	6.4%	0.31 [-0.08, 0.71]
**Subtotal (95% CI)**			**390**			**355**	**44.1%**	**-0.11 [-0.43, 0.22]**
Heterogeneity: Tau² = 0.14; Chi² = 26.90, df = 6 (P = 0.0002); I² = 78%								
Test for overall effect: Z = 0.64 (P = 0.52)								
**Total (95% CI)**			**728**			**650**	**100.0%**	**-0.30 [-0.60, 0.01]**
Heterogeneity: Tau² = 0.33; Chi² = 112.03, df = 16 (P < 0.00001); I² = 86%								
Test for overall effect: Z = 1.91 (P = 0.06)								
Test for subgroup differences: Chi² = 1.50, df = 1 (P = 0.22), I² = 33.4%								

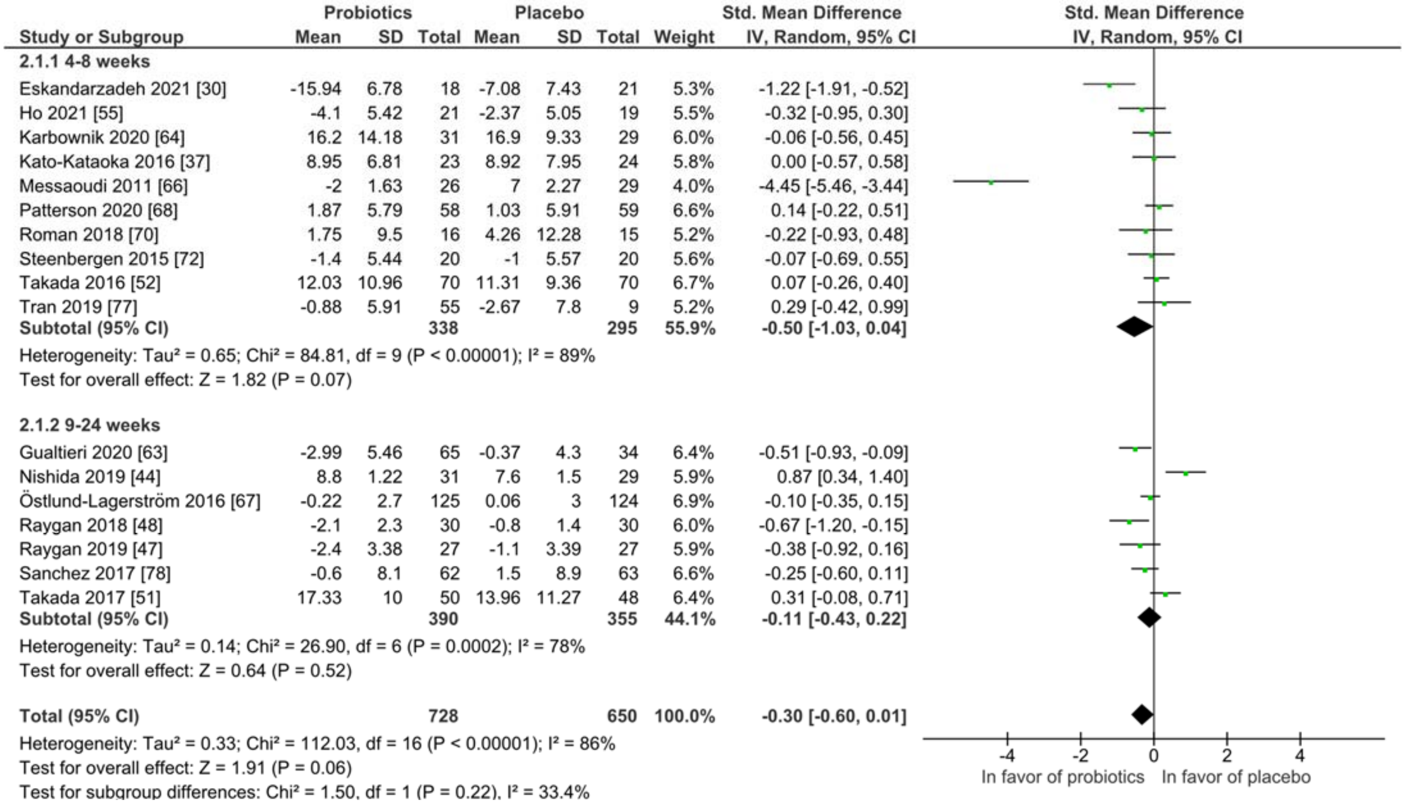

Figure S2. Effect of single-strain versus multi-strain probiotics on Anxiety in randomized controlled trials receiving either probiotics or placebo treatment.

Study or Subgroup	Probiotics Mean	SD	Total	Placebo Mean	SD	Total	Weight	Std. Mean Difference IV, Random, 95% CI
**3.1.1 single-strain**								
Ho 2021 [55]	-4.1	5.42	21	-2.37	5.05	19	5.5%	-0.32 [-0.95, 0.30]
Karbownik 2020 [64]	16.2	14.18	31	16.9	9.33	29	6.0%	-0.06 [-0.56, 0.45]
Kato-Kataoka 2016 [37]	8.95	6.81	23	8.92	7.95	24	5.8%	0.00 [-0.57, 0.58]
Nishida 2019 [44]	8.8	1.22	31	7.6	1.5	29	5.9%	0.87 [0.34, 1.40]
Östlund-Lagerström 2016 [67]	-0.22	2.7	125	0.06	3	124	6.9%	-0.10 [-0.35, 0.15]
Patterson 2020 [68]	1.87	5.79	58	1.03	5.91	59	6.6%	0.14 [-0.22, 0.51]
Sanchez 2017 [78]	-0.6	8.1	62	1.5	8.9	63	6.6%	-0.25 [-0.60, 0.11]
Takada 2016 [52]	12.03	10.96	70	11.31	9.36	70	6.7%	0.07 [-0.26, 0.40]
Takada 2017 [51]	17.33	10	50	13.96	11.27	48	6.4%	0.31 [-0.08, 0.71]
**Subtotal (95% CI)**			**471**			**465**	**56.5%**	**0.06 [-0.13, 0.26]**
Heterogeneity: Tau² = 0.04; Chi² = 16.67, df = 8 (P = 0.03); I² = 52%								
Test for overall effect: Z = 0.63 (P = 0.53)								
**3.1.2 multi-strain**								
Eskandarzadeh 2021 [30]	-15.94	6.78	18	-7.08	7.43	21	5.3%	-1.22 [-1.91, -0.52]
Gualtieri 2020 [63]	-2.99	5.46	65	-0.37	4.3	34	6.4%	-0.51 [-0.93, -0.09]
Messaoudi 2011 [66]	-2	1.63	26	7	2.27	29	4.0%	-4.45 [-5.46, -3.44]
Raygan 2018 [48]	-2.1	2.3	30	-0.8	1.4	30	6.0%	-0.67 [-1.20, -0.15]
Raygan 2019 [47]	-2.4	3.38	27	-1.1	3.39	27	5.9%	-0.38 [-0.92, 0.16]
Roman 2018 [70]	1.75	9.5	16	4.26	12.28	15	5.2%	-0.22 [-0.93, 0.48]
Steenbergen 2015 [72]	-1.4	5.44	20	-1	5.57	20	5.6%	-0.07 [-0.69, 0.55]
Tran 2019 [77]	-0.88	5.91	55	-2.67	7.8	9	5.2%	0.29 [-0.42, 0.99]
**Subtotal (95% CI)**			**257**			**185**	**43.5%**	**-0.84 [-1.52, -0.16]**
Heterogeneity: Tau² = 0.86; Chi² = 69.41, df = 7 (P < 0.00001); I² = 90%								
Test for overall effect: Z = 2.41 (P = 0.02)								
**Total (95% CI)**			**728**			**650**	**100.0%**	**-0.30 [-0.60, 0.01]**
Heterogeneity: Tau² = 0.33; Chi² = 112.03, df = 16 (P < 0.00001); I² = 86%								
Test for overall effect: Z = 1.91 (P = 0.06)								
Test for subgroup differences: Chi² = 6.20, df = 1 (P = 0.01), I² = 83.9%								

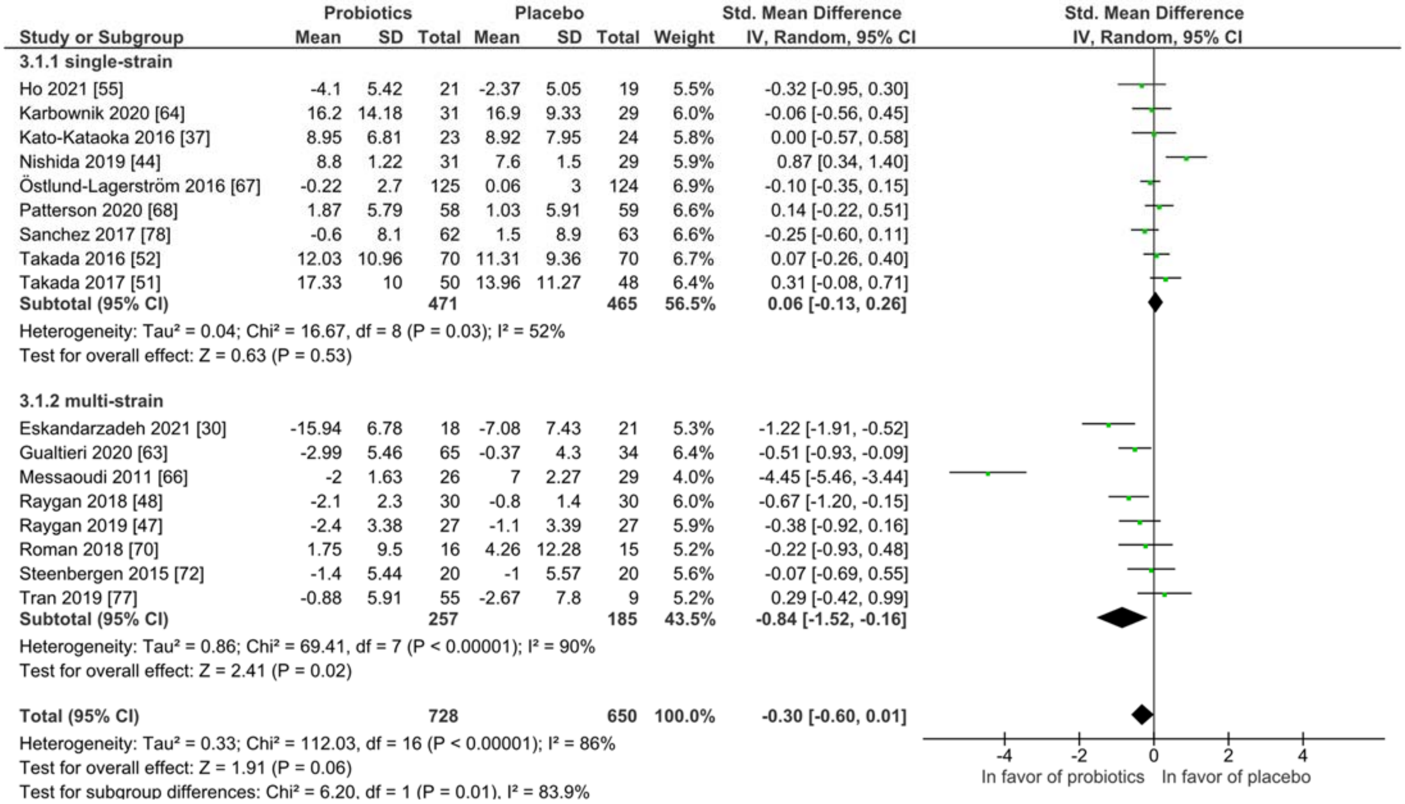

Figure S3. Effect of studies from Asia versus studies from Europe on Anxiety in randomized controlled trials receiving either probiotics or placebo treatment.

Study or Subgroup	Probiotics Mean	Probiotics SD	Probiotics Total	Placebo Mean	Placebo SD	Placebo Total	Weight	Std. Mean Difference IV, Random, 95% CI
**4.1.1 Asia**								
Ho 2021 [55]	-4.1	5.42	21	-2.37	5.05	19	5.5%	-0.32 [-0.95, 0.30]
Karbownik 2020 [64]	16.2	14.18	31	16.9	9.33	29	6.0%	-0.06 [-0.56, 0.45]
Kato-Kataoka 2016 [37]	8.95	6.81	23	8.92	7.95	24	5.8%	0.00 [-0.57, 0.58]
Nishida 2019 [44]	8.8	1.22	31	7.6	1.5	29	5.9%	0.87 [0.34, 1.40]
Raygan 2018 [48]	-2.1	2.3	30	-0.8	1.4	30	6.0%	-0.67 [-1.20, -0.15]
Raygan 2019 [47]	-2.4	3.38	27	-1.1	3.39	27	5.9%	-0.38 [-0.92, 0.16]
Takada 2016 [52]	12.03	10.96	70	11.31	9.36	70	6.7%	0.07 [-0.26, 0.40]
Takada 2017 [51]	17.33	10	50	13.96	11.27	48	6.4%	0.31 [-0.08, 0.71]
**Subtotal (95% CI)**			**283**			**276**	**48.3%**	**-0.01 [-0.31, 0.30]**
Heterogeneity: Tau² = 0.13; Chi² = 22.16, df = 7 (P = 0.002); I² = 68%								
Test for overall effect: Z = 0.05 (P = 0.96)								
**4.1.2 Not Asia**								
Eskandarzadeh 2021 [30]	-15.94	6.78	18	-7.08	7.43	21	5.3%	-1.22 [-1.91, -0.52]
Gualtieri 2020 [63]	-2.99	5.46	65	-0.37	4.3	34	6.4%	-0.51 [-0.93, -0.09]
Messaoudi 2011 [66]	-2	1.63	26	7	2.27	29	4.0%	-4.45 [-5.46, -3.44]
Östlund-Lagerström 2016 [67]	-0.22	2.7	125	0.06	3	124	6.9%	-0.10 [-0.35, 0.15]
Patterson 2020 [68]	1.87	5.79	58	1.03	5.91	59	6.6%	0.14 [-0.22, 0.51]
Roman 2018 [70]	1.75	9.5	16	4.26	12.28	15	5.2%	-0.22 [-0.93, 0.48]
Sanchez 2017 [78]	-0.6	8.1	62	1.5	8.9	63	6.6%	-0.25 [-0.60, 0.11]
Steenbergen 2015 [72]	-1.4	5.44	20	-1	5.57	20	5.6%	-0.07 [-0.69, 0.55]
Tran 2019 [77]	-0.88	5.91	55	-2.67	7.8	9	5.2%	0.29 [-0.42, 0.99]
**Subtotal (95% CI)**			**445**			**374**	**51.7%**	**-0.60 [-1.11, -0.09]**
Heterogeneity: Tau² = 0.52; Chi² = 83.52, df = 8 (P < 0.00001); I² = 90%								
Test for overall effect: Z = 2.30 (P = 0.02)								
**Total (95% CI)**			**728**			**650**	**100.0%**	**-0.30 [-0.60, 0.01]**
Heterogeneity: Tau² = 0.33; Chi² = 112.03, df = 16 (P < 0.00001); I² = 86%								
Test for overall effect: Z = 1.91 (P = 0.06)								
Test for subgroup differences: Chi² = 3.78, df = 1 (P = 0.05), I² = 73.6%								

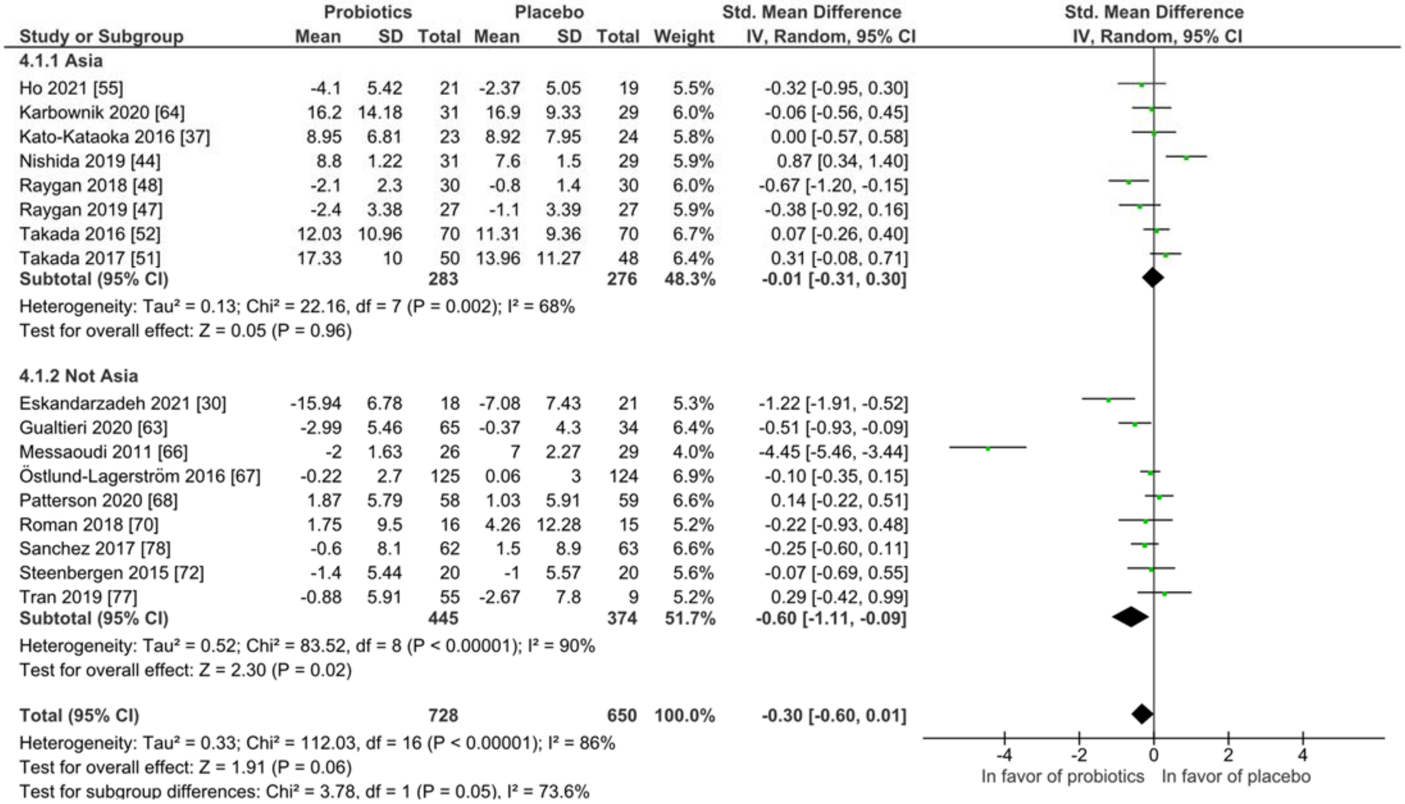

Figure S4. Effect of application form on Anxiety of randomized controlled trials receiving either probiotics or placebo treatment.

Study or Subgroup	Probiotics Mean	Probiotics SD	Probiotics Total	Placebo Mean	Placebo SD	Placebo Total	Weight	Std. Mean Difference IV, Random, 95% CI
**5.1.1 liquid/powder**								
Gualtieri 2020 [63]	-2.99	5.46	65	-0.37	4.3	34	6.4%	-0.51 [-0.93, -0.09]
Kato-Kataoka 2016 [37]	8.95	6.81	23	8.92	7.95	24	5.8%	0.00 [-0.57, 0.58]
Messaoudi 2011 [66]	-2	1.63	26	7	2.27	29	4.0%	-4.45 [-5.46, -3.44]
Östlund-Lagerström 2016 [67]	-0.22	2.7	125	0.06	3	124	6.9%	-0.10 [-0.35, 0.15]
Steenbergen 2015 [72]	-1.4	5.44	20	-1	5.57	20	5.6%	-0.07 [-0.69, 0.55]
Takada 2016 [52]	12.03	10.96	70	11.31	9.36	70	6.7%	0.07 [-0.26, 0.40]
Takada 2017 [51]	17.33	10	50	13.96	11.27	48	6.4%	0.31 [-0.08, 0.71]
**Subtotal (95% CI)**			**379**			**349**	**41.7%**	**-0.53 [-1.12, 0.06]**
Heterogeneity: Tau² = 0.56; Chi² = 79.51, df = 6 (P < 0.00001); I² = 92%								
Test for overall effect: Z = 1.76 (P = 0.08)								
**5.1.2 capsule/pills**								
Eskandarzadeh 2021 [30]	-15.94	6.78	18	-7.08	7.43	21	5.3%	-1.22 [-1.91, -0.52]
Ho 2021 [55]	-4.1	5.42	21	-2.37	5.05	19	5.5%	-0.32 [-0.95, 0.30]
Karbownik 2020 [64]	16.2	14.18	31	16.9	9.33	29	6.0%	-0.06 [-0.56, 0.45]
Nishida 2019 [44]	8.8	1.22	31	7.6	1.5	29	5.9%	0.87 [0.34, 1.40]
Patterson 2020 [68]	1.87	5.79	58	1.03	5.91	59	6.6%	0.14 [-0.22, 0.51]
Roman 2018 [70]	1.75	9.5	16	4.26	12.28	15	5.2%	-0.22 [-0.93, 0.48]
Sanchez 2017 [78]	-0.6	8.1	62	1.5	8.9	63	6.6%	-0.25 [-0.60, 0.11]
Tran 2019 [77]	-0.88	5.91	55	-2.67	7.8	9	5.2%	0.29 [-0.42, 0.99]
**Subtotal (95% CI)**			**292**			**244**	**46.4%**	**-0.07 [-0.44, 0.29]**
Heterogeneity: Tau² = 0.19; Chi² = 26.53, df = 7 (P = 0.0004); I² = 74%								
Test for overall effect: Z = 0.40 (P = 0.69)								
**5.1.3 Not reported**								
Raygan 2018 [48]	-2.1	2.3	30	-0.8	1.4	30	6.0%	-0.67 [-1.20, -0.15]
Raygan 2019 [47]	-2.4	3.38	27	-1.1	3.39	27	5.9%	-0.38 [-0.92, 0.16]
**Subtotal (95% CI)**			**57**			**57**	**11.9%**	**-0.53 [-0.91, -0.16]**
Heterogeneity: Tau² = 0.00; Chi² = 0.60, df = 1 (P = 0.44); I² = 0%								
Test for overall effect: Z = 2.78 (P = 0.005)								
**Total (95% CI)**			**728**			**650**	**100.0%**	**-0.30 [-0.60, 0.01]**
Heterogeneity: Tau² = 0.33; Chi² = 112.03, df = 16 (P < 0.00001); I² = 86%								
Test for overall effect: Z = 1.91 (P = 0.06)								
Test for subgroup differences: Chi² = 3.47, df = 2 (P = 0.18), I² = 42.4%								

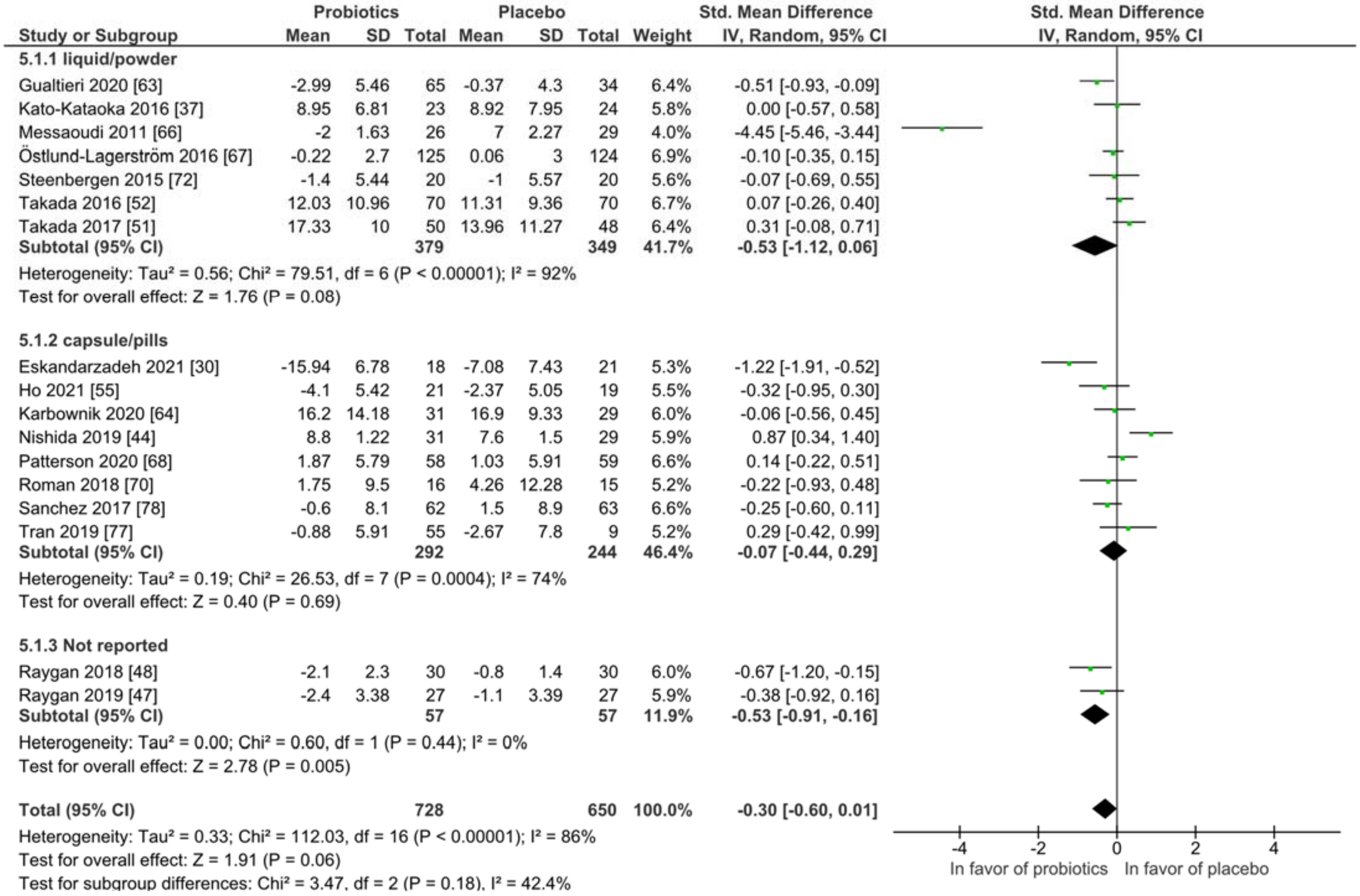

Figure S5. Effect of studies with healthy participants on Anxiety in randomized controlled trials receiving either probiotics or placebo treatment. Only one study included patients with diagnosed anxiety so that no subgroup-analysis could be conducted for the patient group.

Study or Subgroup	Probiotics Mean	Probiotics SD	Probiotics Total	Placebo Mean	Placebo SD	Placebo Total	Weight	Std. Mean Difference IV, Random, 95% CI
Gualtieri 2020 [63]	-2.99	5.46	65	-0.37	4.3	34	6.7%	-0.51 [-0.93, -0.09]
Ho 2021 [55]	-4.1	5.42	21	-2.37	5.05	19	5.8%	-0.32 [-0.95, 0.30]
Karbownik 2020 [64]	16.2	14.18	31	16.9	9.33	29	6.4%	-0.06 [-0.56, 0.45]
Kato-Kataoka 2016 [37]	8.95	6.81	23	8.92	7.95	24	6.1%	0.00 [-0.57, 0.58]
Messaoudi 2011 [66]	-2	1.63	26	7	2.27	29	4.2%	-4.45 [-5.46, -3.44]
Nishida 2019 [44]	8.8	1.22	31	7.6	1.5	29	6.3%	0.87 [0.34, 1.40]
Östlund-Lagerström 2016 [67]	-0.22	2.7	125	0.06	3	124	7.4%	-0.10 [-0.35, 0.15]
Patterson 2020 [68]	1.87	5.79	58	1.03	5.91	59	7.0%	0.14 [-0.22, 0.51]
Raygan 2018 [48]	-2.1	2.3	30	-0.8	1.4	30	6.3%	-0.67 [-1.20, -0.15]
Raygan 2019 [47]	-2.4	3.38	27	-1.1	3.39	27	6.2%	-0.38 [-0.92, 0.16]
Roman 2018 [70]	1.75	9.5	16	4.26	12.28	15	5.5%	-0.22 [-0.93, 0.48]
Sanchez 2017 [78]	-0.6	8.1	62	1.5	8.9	63	7.0%	-0.25 [-0.60, 0.11]
Steenbergen 2015 [72]	-1.4	5.44	20	-1	5.57	20	5.9%	-0.07 [-0.69, 0.55]
Takada 2016 [52]	12.03	10.96	70	11.31	9.36	70	7.1%	0.07 [-0.26, 0.40]
Takada 2017 [51]	17.33	10	50	13.96	11.27	48	6.8%	0.31 [-0.08, 0.71]
Tran 2019 [77]	-0.88	5.91	55	-2.67	7.8	9	5.5%	0.29 [-0.42, 0.99]
**Total (95% CI)**			**710**			**629**	**100.0%**	**-0.24 [-0.55, 0.06]**

Heterogeneity: Tau² = 0.31; Chi² = 102.44, df = 15 (P < 0.00001); I² = 85%
Test for overall effect: Z = 1.56 (P = 0.12)

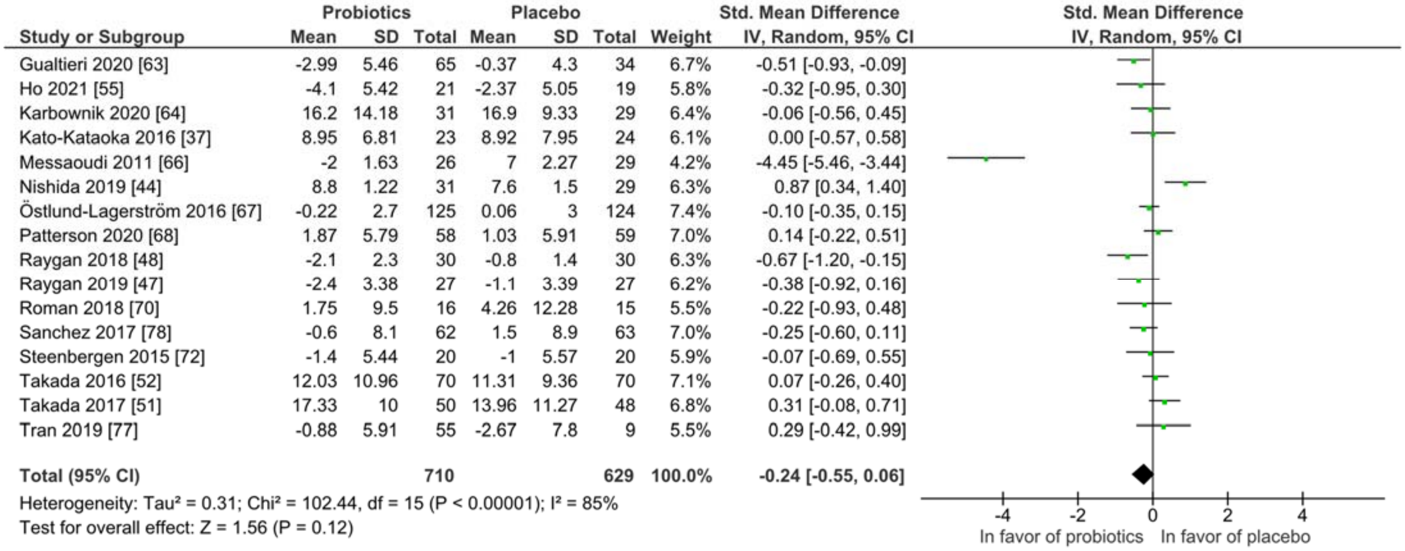

Figure S6. Overview of Anxiety symptom outcomes according to questionnaire. Since several studies incorporated more than one questionnaire for the same outcome, no total summary across all questionnaires is presented here.

Study or Subgroup	Probiotics Mean	Probiotics SD	Probiotics Total	Placebo Mean	Placebo SD	Placebo Total	Weight	Std. Mean Difference IV, Random, 95% CI
**1.1.1 STAI state**								
Eskandarzadeh 2021 [30] STAI state	-15.94	6.78	18	-7.08	7.43	21	3.6%	-1.22 [-1.91, -0.52]
Karbownik 2020 [64] STAI state	16.2	14.18	31	16.9	9.33	29	4.1%	-0.06 [-0.56, 0.45]
Kato-Kataoka 2016 [37] STAI state	8.95	6.81	23	8.92	7.95	24	3.9%	0.00 [-0.57, 0.58]
Nishida 2019 [44] STAI state	8.8	1.22	31	7.6	1.5	29	4.1%	0.87 [0.34, 1.40]
Patterson 2020 [68] STAI state	1.87	5.79	58	1.03	5.91	59	4.5%	0.14 [-0.22, 0.51]
Roman 2018 [70] STAI state	1.75	9.5	16	4.26	12.28	15	3.6%	-0.22 [-0.93, 0.48]
Sanchez 2017 [78] STAI state	-0.6	8.1	62	1.5	8.9	63	4.5%	-0.25 [-0.60, 0.11]
Takada 2016 [52] STAI state	12.03	10.96	70	11.31	9.36	70	4.6%	0.07 [-0.26, 0.40]
Takada 2017 [51] STAI state	17.33	10	50	13.96	11.27	48	4.4%	0.31 [-0.08, 0.71]
**Subtotal (95% CI)**			**359**			**358**	**37.3%**	**0.00 [-0.29, 0.29]**
Heterogeneity: Tau² = 0.13; Chi² = 27.42, df = 8 (P = 0.0006); I² = 71%								
Test for overall effect: Z = 0.00 (P = 1.00)								
**1.1.2 STAI trait**								
Eskandarzadeh 2021 [30] STAI trait	-11.04	7.36	18	-7.58	7.12	21	3.8%	-0.47 [-1.11, 0.17]
Nishida 2019 [44] STAI trait	-1.9	1.24	31	1.1	1.52	29	3.7%	-2.14 [-2.79, -1.50]
Roman 2018 [70] STAI trait	-3	8.5	16	-3.4	9.65	15	3.6%	0.04 [-0.66, 0.75]
Sanchez 2017 [78] STAI trait	-2.7	5.4	62	-1.4	5.8	62	4.5%	-0.23 [-0.58, 0.12]
**Subtotal (95% CI)**			**127**			**127**	**15.6%**	**-0.69 [-1.58, 0.19]**
Heterogeneity: Tau² = 0.72; Chi² = 29.47, df = 3 (P < 0.00001); I² = 90%								
Test for overall effect: Z = 1.53 (P = 0.13)								
**1.1.3 BAI**								
Eskandarzadeh 2021 [30] BAI	-16.15	9.27	18	-11.25	8.34	21	3.7%	-0.55 [-1.19, 0.10]
Ho 2021 [55] BAI	-4.1	5.42	21	-2.37	5.05	19	3.8%	-0.32 [-0.95, 0.30]
Patterson 2020 [68] BAI	-0.83	3.54	58	0.42	4.94	59	4.5%	-0.29 [-0.65, 0.08]
Raygan 2018 [48] BAI	-2.1	2.3	30	-0.8	1.4	30	4.1%	-0.67 [-1.20, -0.15]
Raygan 2019 [47] BAI	-2.4	3.38	27	-1.1	3.39	27	4.0%	-0.38 [-0.92, 0.16]
Steenbergen 2015 [72] BAI	-1.4	5.44	20	-1	5.57	20	3.8%	-0.07 [-0.69, 0.55]
Tran 2019 [77] BAI	-0.88	5.91	55	-2.67	7.8	9	3.6%	0.29 [-0.42, 0.99]
**Subtotal (95% CI)**			**229**			**185**	**27.5%**	**-0.32 [-0.52, -0.12]**
Heterogeneity: Tau² = 0.00; Chi² = 5.77, df = 6 (P = 0.45); I² = 0%								
Test for overall effect: Z = 3.07 (P = 0.002)								
**1.1.4 HADS-A**								
Messaoudi 2011 [66] HADS-A	-2	1.63	26	7	2.27	29	2.7%	-4.45 [-5.46, -3.44]
Nishida 2019 [44] HADS-A	0.7	2.31	31	1.3	2.58	29	4.1%	-0.24 [-0.75, 0.27]
Östlund-Lagerström 2016 [67] HADS-A	-0.22	2.7	125	0.06	3	124	4.7%	-0.10 [-0.35, 0.15]
**Subtotal (95% CI)**			**182**			**182**	**11.6%**	**-1.51 [-3.21, 0.20]**
Heterogeneity: Tau² = 2.17; Chi² = 67.07, df = 2 (P < 0.00001); I² = 97%								
Test for overall effect: Z = 1.73 (P = 0.08)								
**1.1.5 HAM-A**								
Eskandarzadeh 2021 [30] HAM-A	-16.07	6.63	18	-8.79	6.1	21	3.6%	-1.12 [-1.81, -0.44]
Gualtieri 2020 [63] HAM-A	-2.99	5.46	65	-0.37	4.3	34	4.4%	-0.51 [-0.93, -0.09]
**Subtotal (95% CI)**			**83**			**55**	**8.0%**	**-0.76 [-1.34, -0.17]**
Heterogeneity: Tau² = 0.10; Chi² = 2.25, df = 1 (P = 0.13); I² = 55%								
Test for overall effect: Z = 2.52 (P = 0.01)								

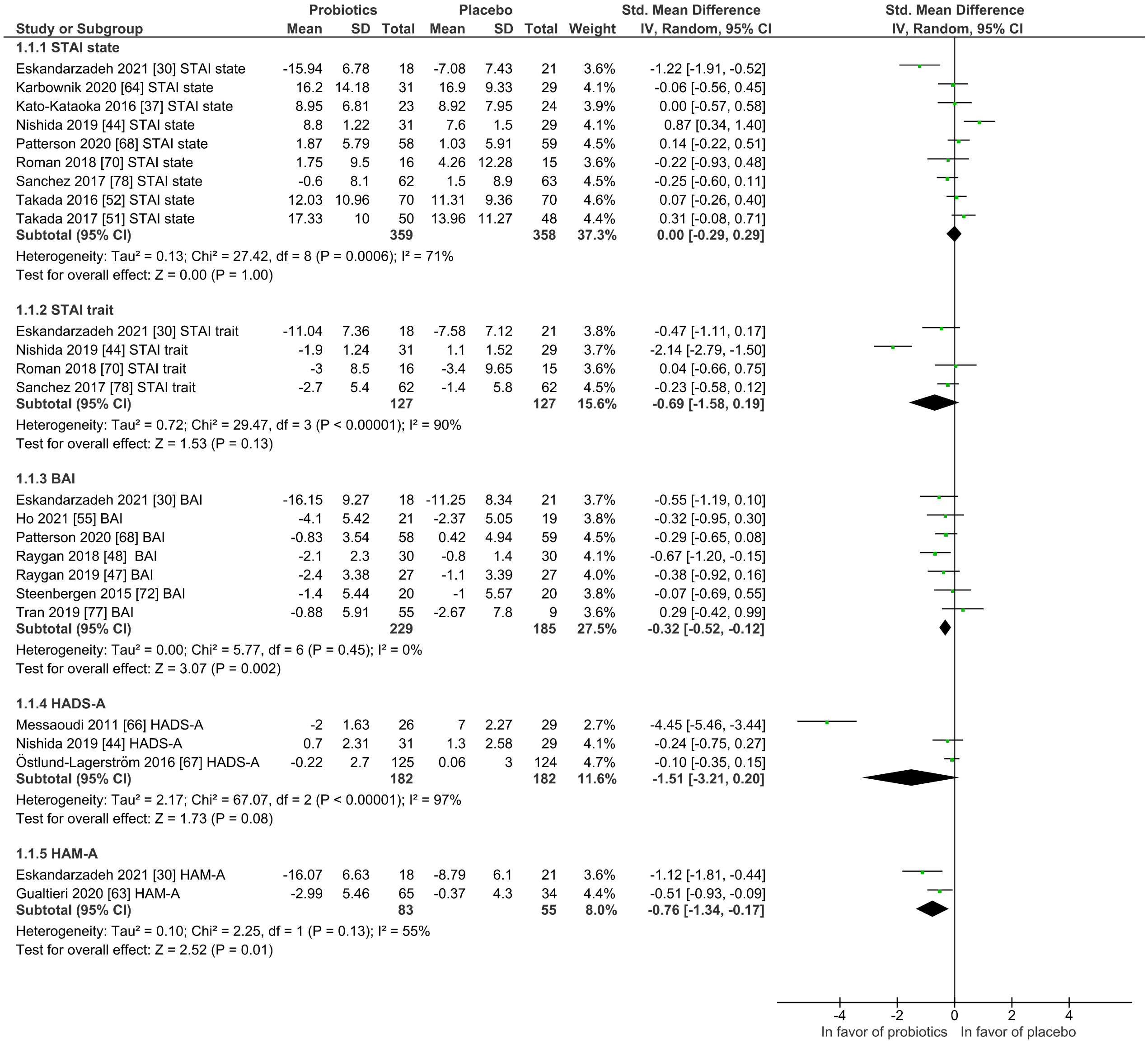

Figure S7. Effect of intake period on Depression of randomized controlled trials receiving either probiotics or placebo treatment.

Study or Subgroup	Probiotics Mean	Probiotics SD	Probiotics Total	Placebo Mean	Placebo SD	Placebo Total	Weight	Std. Mean Difference IV, Random, 95% CI
**2.1.1 4-8 weeks**								
Akkasheh 2016 [32]	-5.3	5.37	20	-1.8	5.37	20	5.0%	-0.64 [-1.28, -0.00]
Ho 2021 [55]	-5.1	6.85	21	-0.26	5.46	19	4.9%	-0.76 [-1.41, -0.12]
Kazemi 2019 [38]	-9.25	7.99	38	-3.19	10.34	36	7.2%	-0.65 [-1.12, -0.18]
Messaoudi 2011 [66]	-2.5	2.54	26	-1	2.03	29	6.1%	-0.65 [-1.19, -0.10]
Papalini 2019 [20]	-0.32	1.51	29	-0.38	2.23	29	6.5%	0.03 [-0.48, 0.55]
Reininghaus 2020 [69]	-15.64	6.01	28	-14.4	8.27	30	6.5%	-0.17 [-0.68, 0.35]
Roman 2018 [70]	-2.87	6.33	16	-5.8	10.86	15	4.3%	0.32 [-0.39, 1.03]
Rudzki 2019 [71]	-15.5	4.18	40	-12	5.7	39	7.4%	-0.69 [-1.15, -0.24]
Steenbergen 2015 [72]	-0.65	3.68	20	0	3.68	20	5.2%	-0.17 [-0.79, 0.45]
**Subtotal (95% CI)**			**238**			**237**	**53.2%**	**-0.40 [-0.64, -0.16]**
Heterogeneity: Tau² = 0.05; Chi² = 13.22, df = 8 (P = 0.10); I² = 39%								
Test for overall effect: Z = 3.27 (P = 0.001)								
**2.1.2 9-24 weeks**								
Nishida 2019 [44]	1.2	3.08	31	1.5	2.62	29	6.6%	-0.10 [-0.61, 0.40]
Östlund-Lagerström 2016 [67]	0.04	2.2	125	-0.01	2.8	124	11.4%	0.02 [-0.23, 0.27]
Raygan 2018 [48]	-2.8	3.8	30	-0.9	2.1	30	6.5%	-0.61 [-1.13, -0.09]
Raygan 2019 [47]	-2.7	3.12	27	-1	2.87	27	6.1%	-0.56 [-1.10, -0.01]
Sanchez 2017 [78]	-1.5	3	62	0.5	3.3	63	9.1%	-0.63 [-0.99, -0.27]
Zhang 2021 [57]	-8.34	5.08	38	-6.61	5.17	31	7.1%	-0.33 [-0.81, 0.14]
**Subtotal (95% CI)**			**313**			**304**	**46.8%**	**-0.34 [-0.61, -0.07]**
Heterogeneity: Tau² = 0.06; Chi² = 12.31, df = 5 (P = 0.03); I² = 59%								
Test for overall effect: Z = 2.50 (P = 0.01)								
**Total (95% CI)**			**551**			**541**	**100.0%**	**-0.37 [-0.55, -0.20]**
Heterogeneity: Tau² = 0.05; Chi² = 26.85, df = 14 (P = 0.02); I² = 48%								
Test for overall effect: Z = 4.14 (P < 0.0001)								
Test for subgroup differences: Chi² = 0.09, df = 1 (P = 0.77), I² = 0%								

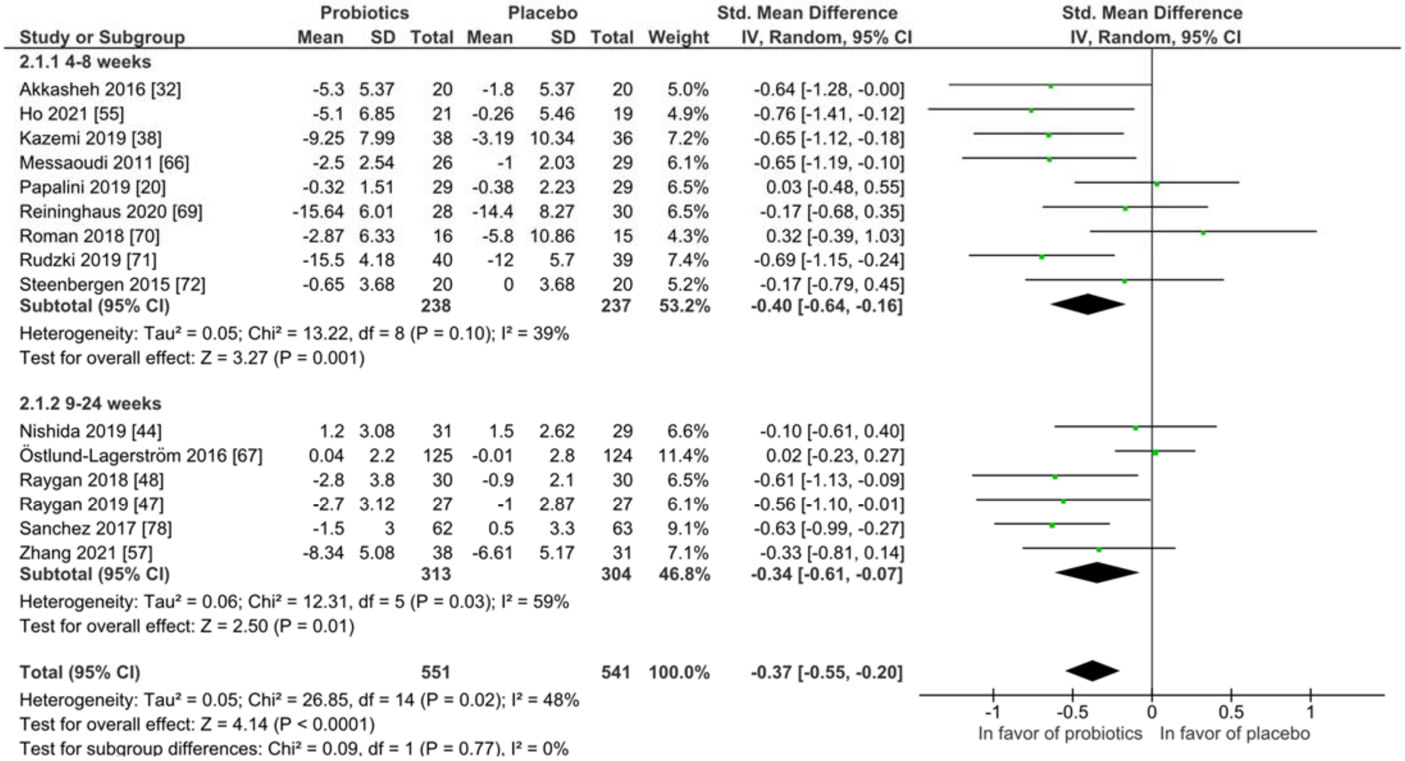

Figure S8. Effect of single-strain versus multi-strain probiotics on Depression in randomized controlled trials receiving either probiotics or placebo treatment.

Study or Subgroup	Probiotics Mean	Probiotics SD	Probiotics Total	Placebo Mean	Placebo SD	Placebo Total	Weight	Std. Mean Difference IV, Random, 95% CI
**3.1.1 single-strain**								
Ho 2021 [55]	-5.1	6.85	21	-0.26	5.46	19	4.9%	-0.76 [-1.41, -0.12]
Nishida 2019 [44]	1.2	3.08	31	1.5	2.62	29	6.6%	-0.10 [-0.61, 0.40]
Östlund-Lagerström 2016 [67]	0.04	2.2	125	-0.01	2.8	124	11.4%	0.02 [-0.23, 0.27]
Roman 2018 [70]	-2.87	6.33	16	-5.8	10.86	15	4.3%	0.32 [-0.39, 1.03]
Rudzki 2019 [71]	-15.5	4.18	40	-12	5.7	39	7.4%	-0.69 [-1.15, -0.24]
Sanchez 2017 [78]	-1.5	3	62	0.5	3.3	63	9.1%	-0.63 [-0.99, -0.27]
Zhang 2021 [57]	-8.34	5.08	38	-6.61	5.17	31	7.1%	-0.33 [-0.81, 0.14]
**Subtotal (95% CI)**			**333**			**320**	**50.9%**	**-0.32 [-0.61, -0.03]**
Heterogeneity: Tau² = 0.10; Chi² = 17.83, df = 6 (P = 0.007); I² = 66%								
Test for overall effect: Z = 2.14 (P = 0.03)								
**3.1.2 multi-strain**								
Akkasheh 2016 [32]	-5.3	5.37	20	-1.8	5.37	20	5.0%	-0.64 [-1.28, -0.00]
Kazemi 2019 [38]	-9.25	7.99	38	-3.19	10.34	36	7.2%	-0.65 [-1.12, -0.18]
Messaoudi 2011 [66]	-2.5	2.54	26	-1	2.03	29	6.1%	-0.65 [-1.19, -0.10]
Papalini 2019 [20]	-0.32	1.51	29	-0.38	2.23	29	6.5%	0.03 [-0.48, 0.55]
Raygan 2018 [48]	-2.8	3.8	30	-0.9	2.1	30	6.5%	-0.61 [-1.13, -0.09]
Raygan 2019 [47]	-2.7	3.12	27	-1	2.87	27	6.1%	-0.56 [-1.10, -0.01]
Reininghaus 2020 [69]	-15.64	6.01	28	-14.4	8.27	30	6.5%	-0.17 [-0.68, 0.35]
Steenbergen 2015 [72]	-0.65	3.68	20	0	3.68	20	5.2%	-0.17 [-0.79, 0.45]
**Subtotal (95% CI)**			**218**			**221**	**49.1%**	**-0.43 [-0.62, -0.23]**
Heterogeneity: Tau² = 0.00; Chi² = 7.29, df = 7 (P = 0.40); I² = 4%								
Test for overall effect: Z = 4.33 (P < 0.0001)								
**Total (95% CI)**			**551**			**541**	**100.0%**	**-0.37 [-0.55, -0.20]**
Heterogeneity: Tau² = 0.05; Chi² = 26.85, df = 14 (P = 0.02); I² = 48%								
Test for overall effect: Z = 4.14 (P < 0.0001)								
Test for subgroup differences: Chi² = 0.38, df = 1 (P = 0.54), I² = 0%								

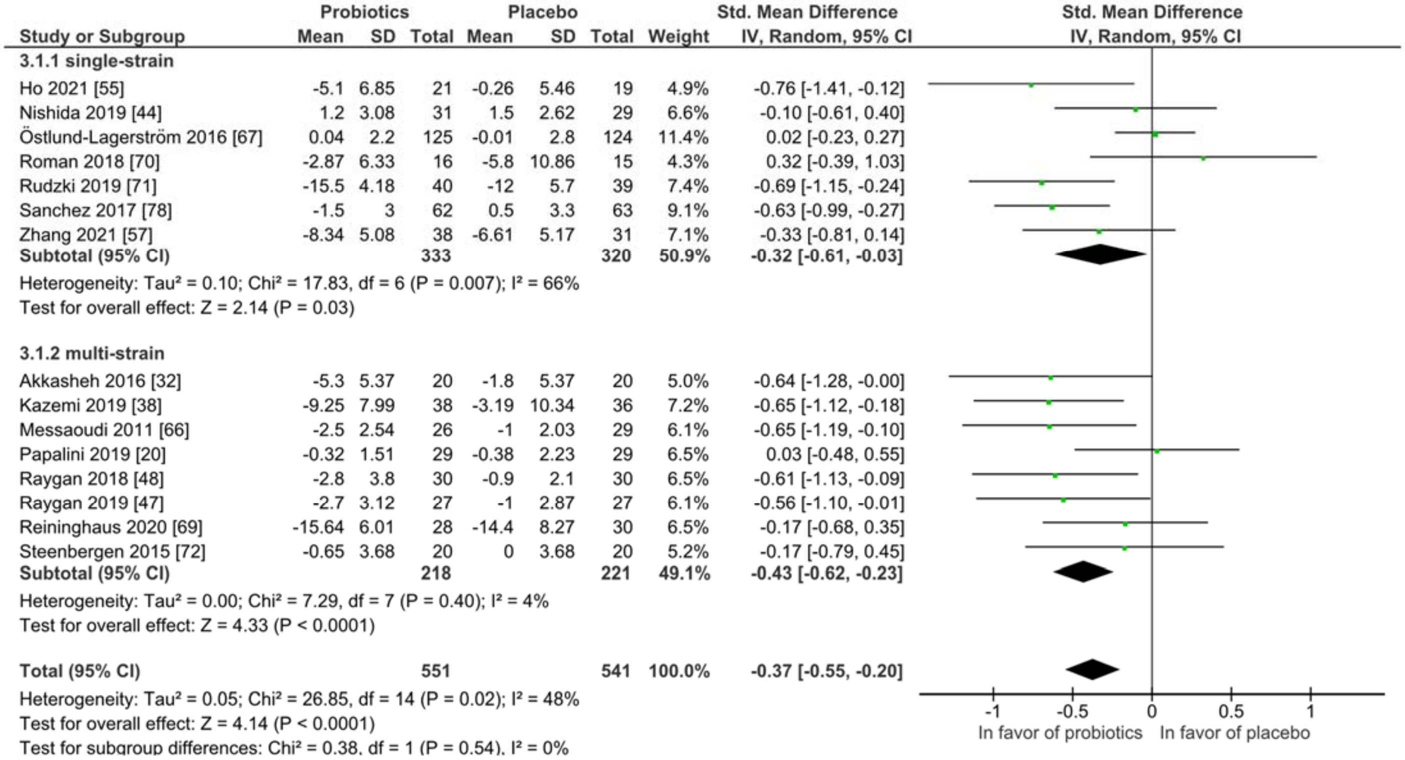

Figure S9. Effect of studies from Asia versus studies from Europe on Depression in randomized controlled trials receiving either probiotics or placebo treatment.

Study or Subgroup	Probiotics Mean	Probiotics SD	Probiotics Total	Placebo Mean	Placebo SD	Placebo Total	Weight	Std. Mean Difference IV, Random, 95% CI
**4.1.1 Asia**								
Akkasheh 2016 [32]	-5.3	5.37	20	-1.8	5.37	20	5.0%	-0.64 [-1.28, -0.00]
Ho 2021 [55]	-5.1	6.85	21	-0.26	5.46	19	4.9%	-0.76 [-1.41, -0.12]
Kazemi 2019 [38]	-9.25	7.99	38	-3.19	10.34	36	7.2%	-0.65 [-1.12, -0.18]
Nishida 2019 [44]	1.2	3.08	31	1.5	2.62	29	6.6%	-0.10 [-0.61, 0.40]
Raygan 2018 [48]	-2.8	3.8	30	-0.9	2.1	30	6.5%	-0.61 [-1.13, -0.09]
Raygan 2019 [47]	-2.7	3.12	27	-1	2.87	27	6.1%	-0.56 [-1.10, -0.01]
Zhang 2021 [57]	-8.34	5.08	38	-6.61	5.17	31	7.1%	-0.33 [-0.81, 0.14]
**Subtotal (95% CI)**			**205**			**192**	**43.4%**	**-0.50 [-0.70, -0.30]**
Heterogeneity: Tau² = 0.00; Chi² = 4.25, df = 6 (P = 0.64); I² = 0%								
Test for overall effect: Z = 4.88 (P < 0.00001)								
**4.1.2 Not Asia**								
Messaoudi 2011 [66]	-2.5	2.54	26	-1	2.03	29	6.1%	-0.65 [-1.19, -0.10]
Östlund-Lagerström 2016 [67]	0.04	2.2	125	-0.01	2.8	124	11.4%	0.02 [-0.23, 0.27]
Papalini 2019 [20]	-0.32	1.51	29	-0.38	2.23	29	6.5%	0.03 [-0.48, 0.55]
Reininghaus 2020 [69]	-15.64	6.01	28	-14.4	8.27	30	6.5%	-0.17 [-0.68, 0.35]
Roman 2018 [70]	-2.87	6.33	16	-5.8	10.86	15	4.3%	0.32 [-0.39, 1.03]
Rudzki 2019 [71]	-15.5	4.18	40	-12	5.7	39	7.4%	-0.69 [-1.15, -0.24]
Sanchez 2017 [78]	-1.5	3	62	0.5	3.3	63	9.1%	-0.63 [-0.99, -0.27]
Steenbergen 2015 [72]	-0.65	3.68	20	0	3.68	20	5.2%	-0.17 [-0.79, 0.45]
**Subtotal (95% CI)**			**346**			**349**	**56.6%**	**-0.26 [-0.53, -0.00]**
Heterogeneity: Tau² = 0.08; Chi² = 18.32, df = 7 (P = 0.01); I² = 62%								
Test for overall effect: Z = 1.96 (P = 0.05)								
**Total (95% CI)**			**551**			**541**	**100.0%**	**-0.37 [-0.55, -0.20]**
Heterogeneity: Tau² = 0.05; Chi² = 26.85, df = 14 (P = 0.02); I² = 48%								
Test for overall effect: Z = 4.14 (P < 0.0001)								
Test for subgroup differences: Chi² = 1.95, df = 1 (P = 0.16), I² = 48.6%								

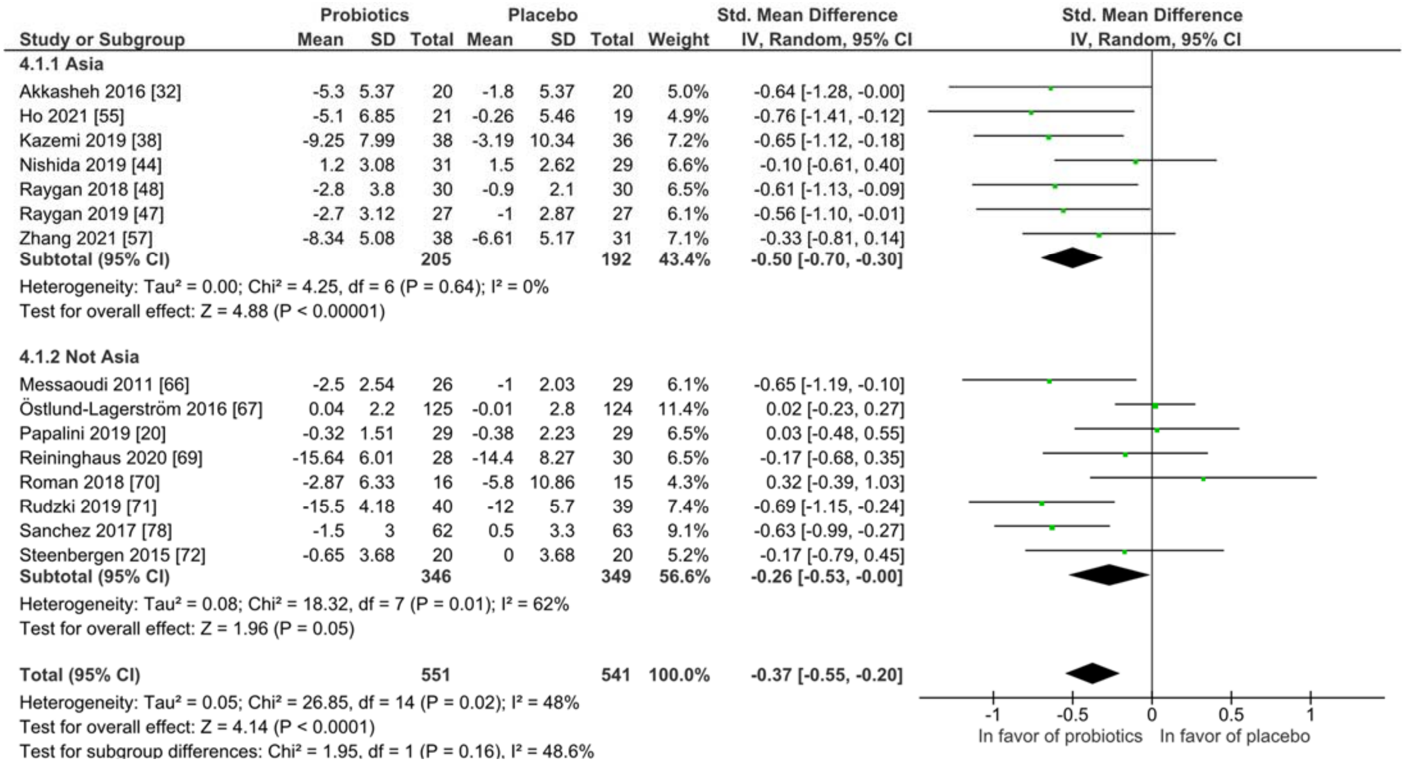

Figure S10. Effect of application form on Depression of randomized controlled trials receiving either probiotics or placebo treatment.

Study or Subgroup	Probiotics Mean	Probiotics SD	Probiotics Total	Placebo Mean	Placebo SD	Placebo Total	Weight	Std. Mean Difference IV, Random, 95% CI
**5.1.1 liquid/powder**								
Kazemi 2019 [38]	-9.25	7.99	38	-3.19	10.34	36	7.2%	-0.65 [-1.12, -0.18]
Messaoudi 2011 [66]	-2.5	2.54	26	-1	2.03	29	6.1%	-0.65 [-1.19, -0.10]
Östlund-Lagerström 2016 [67]	0.04	2.2	125	-0.01	2.8	124	11.4%	0.02 [-0.23, 0.27]
Papalini 2019 [20]	-0.32	1.51	29	-0.38	2.23	29	6.5%	0.03 [-0.48, 0.55]
Reininghaus 2020 [69]	-15.64	6.01	28	-14.4	8.27	30	6.5%	-0.17 [-0.68, 0.35]
Steenbergen 2015 [72]	-0.65	3.68	20	0	3.68	20	5.2%	-0.17 [-0.79, 0.45]
Zhang 2021 [57]	-8.34	5.08	38	-6.61	5.17	31	7.1%	-0.33 [-0.81, 0.14]
**Subtotal (95% CI)**			**304**			**299**	**50.0%**	**-0.24 [-0.47, -0.02]**
Heterogeneity: Tau² = 0.04; Chi² = 10.24, df = 6 (P = 0.11); I² = 41%								
Test for overall effect: Z = 2.11 (P = 0.04)								
**5.1.2 capsule/pills**								
Akkasheh 2016 [32]	-5.3	5.37	20	-1.8	5.37	20	5.0%	-0.64 [-1.28, -0.00]
Ho 2021 [55]	-5.1	6.85	21	-0.26	5.46	19	4.9%	-0.76 [-1.41, -0.12]
Nishida 2019 [44]	1.2	3.08	31	1.5	2.62	29	6.6%	-0.10 [-0.61, 0.40]
Roman 2018 [70]	-2.87	6.33	16	-5.8	10.86	15	4.3%	0.32 [-0.39, 1.03]
Rudzki 2019 [71]	-15.5	4.18	40	-12	5.7	39	7.4%	-0.69 [-1.15, -0.24]
Sanchez 2017 [78]	-1.5	3	62	0.5	3.3	63	9.1%	-0.63 [-0.99, -0.27]
**Subtotal (95% CI)**			**190**			**185**	**37.5%**	**-0.46 [-0.75, -0.16]**
Heterogeneity: Tau² = 0.06; Chi² = 9.54, df = 5 (P = 0.09); I² = 48%								
Test for overall effect: Z = 2.99 (P = 0.003)								
**5.1.3 Not reported**								
Raygan 2018 [48]	-2.8	3.8	30	-0.9	2.1	30	6.5%	-0.61 [-1.13, -0.09]
Raygan 2019 [47]	-2.7	3.12	27	-1	2.87	27	6.1%	-0.56 [-1.10, -0.01]
**Subtotal (95% CI)**			**57**			**57**	**12.6%**	**-0.59 [-0.96, -0.21]**
Heterogeneity: Tau² = 0.00; Chi² = 0.02, df = 1 (P = 0.89); I² = 0%								
Test for overall effect: Z = 3.06 (P = 0.002)								
**Total (95% CI)**			**551**			**541**	**100.0%**	**-0.37 [-0.55, -0.20]**
Heterogeneity: Tau² = 0.05; Chi² = 26.85, df = 14 (P = 0.02); I² = 48%								
Test for overall effect: Z = 4.14 (P < 0.0001)								
Test for subgroup differences: Chi² = 2.76, df = 2 (P = 0.25), I² = 27.6%								

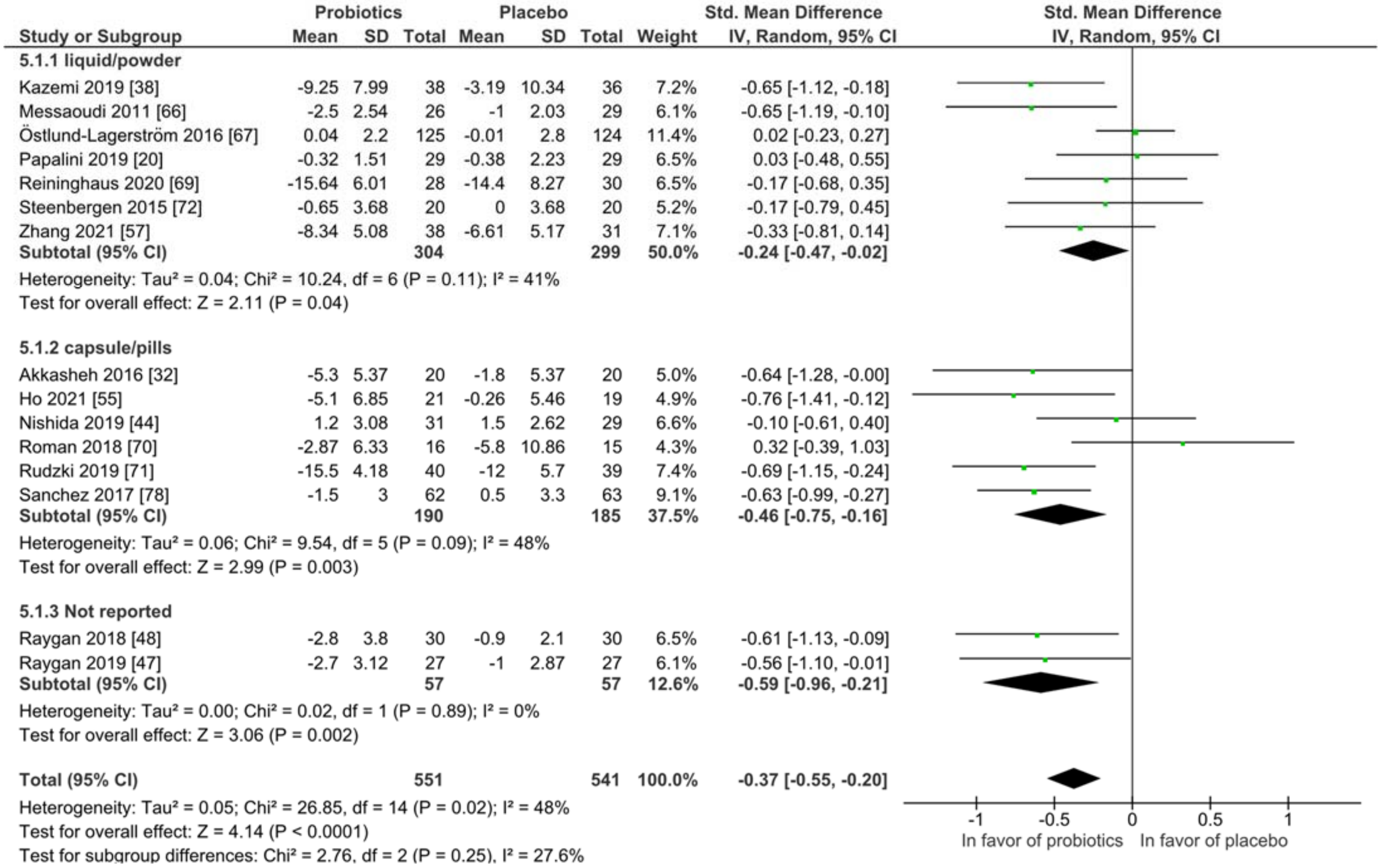

Figure S11. Effect of studies with healthy participants on Depression in randomized controlled trials receiving either probiotics or placebo treatment.

Study or Subgroup	Probiotics Mean	Probiotics SD	Probiotics Total	Placebo Mean	Placebo SD	Placebo Total	Weight	Std. Mean Difference IV, Random, 95% CI
**6.1.1 Healthy**								
Ho 2021 [55]	-5.1	6.85	21	-0.26	5.46	19	4.9%	-0.76 [-1.41, -0.12]
Messaoudi 2011 [66]	-2.5	2.54	26	-1	2.03	29	6.1%	-0.65 [-1.19, -0.10]
Nishida 2019 [44]	1.2	3.08	31	1.5	2.62	29	6.6%	-0.10 [-0.61, 0.40]
Östlund-Lagerström 2016 [67]	0.04	2.2	125	-0.01	2.8	124	11.4%	0.02 [-0.23, 0.27]
Papalini 2019 [20]	-0.32	1.51	29	-0.38	2.23	29	6.5%	0.03 [-0.48, 0.55]
Raygan 2018 [48]	-2.8	3.8	30	-0.9	2.1	30	6.5%	-0.61 [-1.13, -0.09]
Raygan 2019 [47]	-2.7	3.12	27	-1	2.87	27	6.1%	-0.56 [-1.10, -0.01]
Reininghaus 2020 [69]	-15.64	6.01	28	-14.4	8.27	30	6.5%	-0.17 [-0.68, 0.35]
Roman 2018 [70]	-2.87	6.33	16	-5.8	10.86	15	4.3%	0.32 [-0.39, 1.03]
Sanchez 2017 [78]	-1.5	3	62	0.5	3.3	63	9.1%	-0.63 [-0.99, -0.27]
Steenbergen 2015 [72]	-0.65	3.68	20	0	3.68	20	5.2%	-0.17 [-0.79, 0.45]
**Subtotal (95% CI)**			**415**			**415**	**73.3%**	**-0.30 [-0.51, -0.09]**
Heterogeneity: Tau² = 0.06; Chi² = 20.51, df = 10 (P = 0.02); I² = 51%								
Test for overall effect: Z = 2.75 (P = 0.006)								
**6.1.2 Diagnosed Depression Disorder**								
Akkasheh 2016 [32]	-5.3	5.37	20	-1.8	5.37	20	5.0%	-0.64 [-1.28, -0.00]
Kazemi 2019 [38]	-9.25	7.99	38	-3.19	10.34	36	7.2%	-0.65 [-1.12, -0.18]
Rudzki 2019 [71]	-15.5	4.18	40	-12	5.7	39	7.4%	-0.69 [-1.15, -0.24]
Zhang 2021 [57]	-8.34	5.08	38	-6.61	5.17	31	7.1%	-0.33 [-0.81, 0.14]
**Subtotal (95% CI)**			**136**			**126**	**26.7%**	**-0.58 [-0.82, -0.33]**
Heterogeneity: Tau² = 0.00; Chi² = 1.38, df = 3 (P = 0.71); I² = 0%								
Test for overall effect: Z = 4.55 (P < 0.00001)								
**Total (95% CI)**			**551**			**541**	**100.0%**	**-0.37 [-0.55, -0.20]**
Heterogeneity: Tau² = 0.05; Chi² = 26.85, df = 14 (P = 0.02); I² = 48%								
Test for overall effect: Z = 4.14 (P < 0.0001)								
Test for subgroup differences: Chi² = 2.84, df = 1 (P = 0.09), I² = 64.8%								

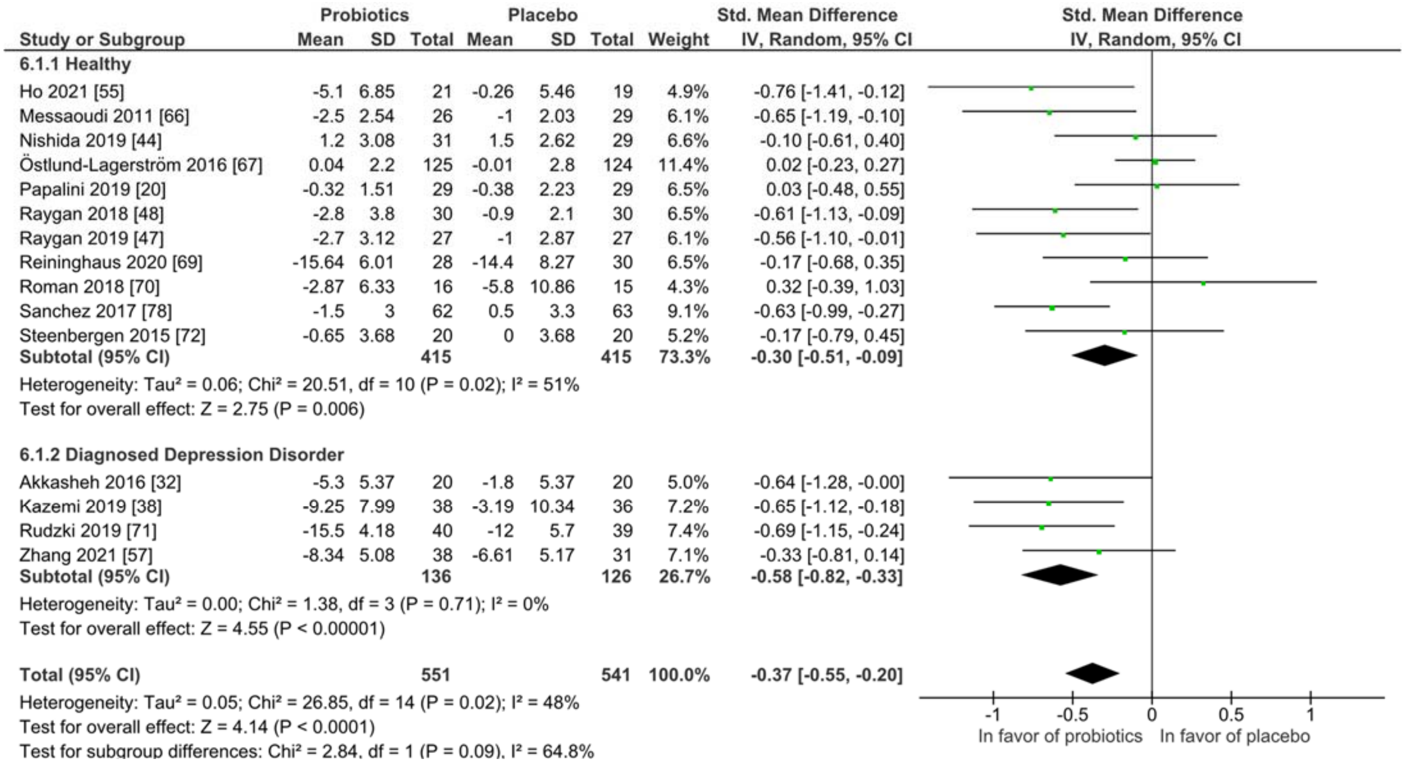

Figure S12. Overview of depression symptom outcomes according to questionnaire. Since several studies incorporated more than one questionnaire for the same outcome, no total summary across all questionnaires is presented here.

Study or Subgroup	Probiotics Mean	Probiotics SD	Probiotics Total	Placebo Mean	Placebo SD	Placebo Total	Weight	Std. Mean Difference IV, Random, 95% CI
**1.1.1 BDI**								
Akkasheh 2016 [32] BDI	-5.3	5.37	20	-1.8	5.37	20	4.4%	-0.64 [-1.28, -0.00]
Ho 2021 [55] BDI	-5.1	6.85	21	-0.26	5.46	19	4.3%	-0.76 [-1.41, -0.12]
Kazemi 2019 [38] BDI	-9.25	7.99	38	-3.19	10.34	36	6.3%	-0.65 [-1.12, -0.18]
Papalini 2019 [20] BDI	-0.32	1.51	29	-0.38	2.23	29	5.7%	0.03 [-0.48, 0.55]
Raygan 2018 [48] BDI	-2.8	3.8	30	-0.9	2.1	30	5.7%	-0.61 [-1.13, -0.09]
Raygan 2019 [47] BDI	-2.7	3.12	27	-1	2.87	27	5.3%	-0.56 [-1.10, -0.01]
Reininghaus 2020 [69] BDI	-15.64	6.01	28	-14.4	8.27	30	5.7%	-0.17 [-0.68, 0.35]
Roman 2018 [70] BDI	-2.87	6.33	16	-5.8	10.86	15	3.7%	0.32 [-0.39, 1.03]
Sanchez 2017 [78] BDI	-1.5	3	62	0.5	3.3	63	8.2%	-0.63 [-0.99, -0.27]
Steenbergen 2015 [72] BDI	-0.65	3.68	20	0	3.68	20	4.5%	-0.17 [-0.79, 0.45]
Zhang 2021 [57] BDI	-8.34	5.08	38	-6.61	5.17	31	6.2%	-0.33 [-0.81, 0.14]
**Subtotal (95% CI)**			**329**			**320**	**60.0%**	**-0.41 [-0.59, -0.23]**
Heterogeneity: Tau² = 0.02; Chi² = 13.36, df = 10 (P = 0.20); I² = 25%								
Test for overall effect: Z = 4.36 (P < 0.0001)								
**1.1.2 HADS-D**								
Messaoudi 2011 [66] HADS-D	-2.5	2.54	26	-1	2.03	29	5.3%	-0.65 [-1.19, -0.10]
Nishida 2019 [44] HADS-D	1.2	3.08	31	1.5	2.62	29	5.8%	-0.10 [-0.61, 0.40]
Östlund-Lagerström 2016 [67] HADS-D	0.04	2.2	125	-0.01	2.8	124	10.4%	0.02 [-0.23, 0.27]
**Subtotal (95% CI)**			**182**			**182**	**21.5%**	**-0.19 [-0.57, 0.19]**
Heterogeneity: Tau² = 0.06; Chi² = 4.78, df = 2 (P = 0.09); I² = 58%								
Test for overall effect: Z = 0.98 (P = 0.33)								
**1.1.3 HAM-D**								
Reininghaus 2020 [69] HAM-D	-5.96	4.35	28	-6.3	4.21	30	5.7%	0.08 [-0.44, 0.59]
Rudzki 2019 [71] HAM-D	-15.5	4.18	40	-12	5.7	39	6.5%	-0.69 [-1.15, -0.24]
Zhang 2021 [57] HAM-D	-12.26	3.38	38	-11.29	4.2	31	6.2%	-0.25 [-0.73, 0.22]
**Subtotal (95% CI)**			**106**			**100**	**18.5%**	**-0.30 [-0.74, 0.14]**
Heterogeneity: Tau² = 0.09; Chi² = 4.98, df = 2 (P = 0.08); I² = 60%								
Test for overall effect: Z = 1.35 (P = 0.18)								

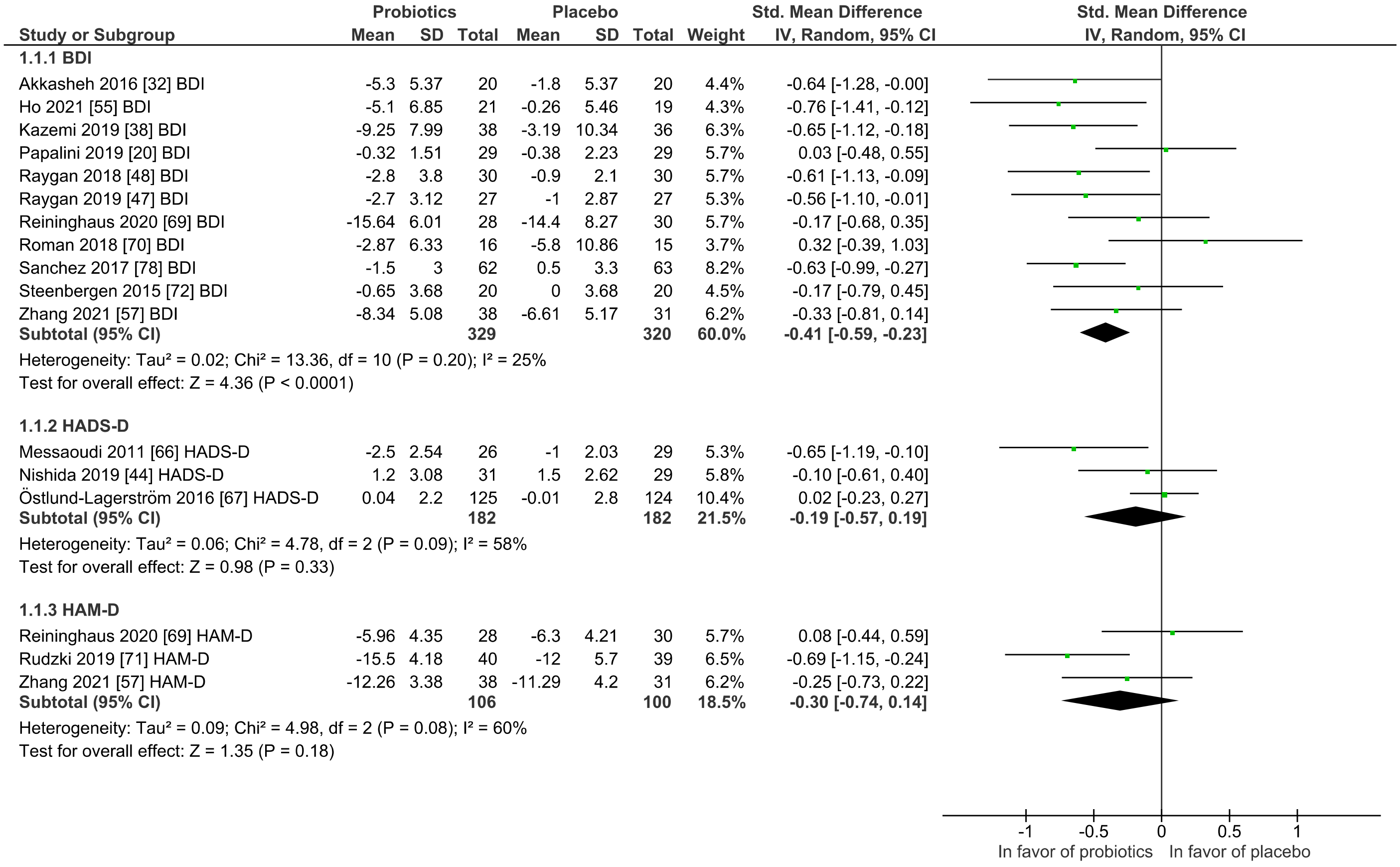

Figure S13. Effect of intake period on Psychiatric Distress of randomized controlled trials receiving either probiotics or placebo treatment.

Study or Subgroup	Probiotics Mean	Probiotics SD	Probiotics Total	Placebo Mean	Placebo SD	Placebo Total	Weight	Std. Mean Difference IV, Random, 95% CI
**2.1.1 4-8 weeks**								
Messaoudi 2011 [66]	-24	18.27	26	-10	26.33	29	10.0%	-0.60 [-1.15, -0.06]
Mohammadi 2016 [41]	-5.8	8.51	25	-3.3	7.6	20	8.8%	-0.30 [-0.89, 0.29]
Reininghaus 2020 [69]	-9.32	6.85	28	-7.37	7.38	30	10.7%	-0.27 [-0.79, 0.25]
Rudzki 2019 [71]	-8.19	4.03	40	-7.91	4.06	39	13.2%	-0.07 [-0.51, 0.37]
**Subtotal (95% CI)**			**119**			**118**	**42.6%**	**-0.28 [-0.54, -0.03]**
Heterogeneity: Tau² = 0.00; Chi² = 2.25, df = 3 (P = 0.52); I² = 0%								
Test for overall effect: Z = 2.15 (P = 0.03)								
**2.1.2 9-24 weeks**								
Gualtieri 2020 [63]	0.03	0.46	65	0.33	0.39	34	13.8%	-0.68 [-1.11, -0.25]
Nishida 2019 [44]	0.9	2.71	31	1.2	0.55	29	11.0%	-0.15 [-0.66, 0.36]
Nishihira 2014 [45]	-0.8	2.43	115	-0.6	2.61	109	22.2%	-0.08 [-0.34, 0.18]
Raygan 2018 [48]	-3.9	4.1	30	-1.1	3.4	30	10.5%	-0.73 [-1.26, -0.21]
**Subtotal (95% CI)**			**241**			**202**	**57.4%**	**-0.38 [-0.74, -0.03]**
Heterogeneity: Tau² = 0.08; Chi² = 8.76, df = 3 (P = 0.03); I² = 66%								
Test for overall effect: Z = 2.11 (P = 0.03)								
**Total (95% CI)**			**360**			**320**	**100.0%**	**-0.33 [-0.53, -0.13]**
Heterogeneity: Tau² = 0.03; Chi² = 11.02, df = 7 (P = 0.14); I² = 36%								
Test for overall effect: Z = 3.19 (P = 0.001)								
Test for subgroup differences: Chi² = 0.20, df = 1 (P = 0.65), I² = 0%								

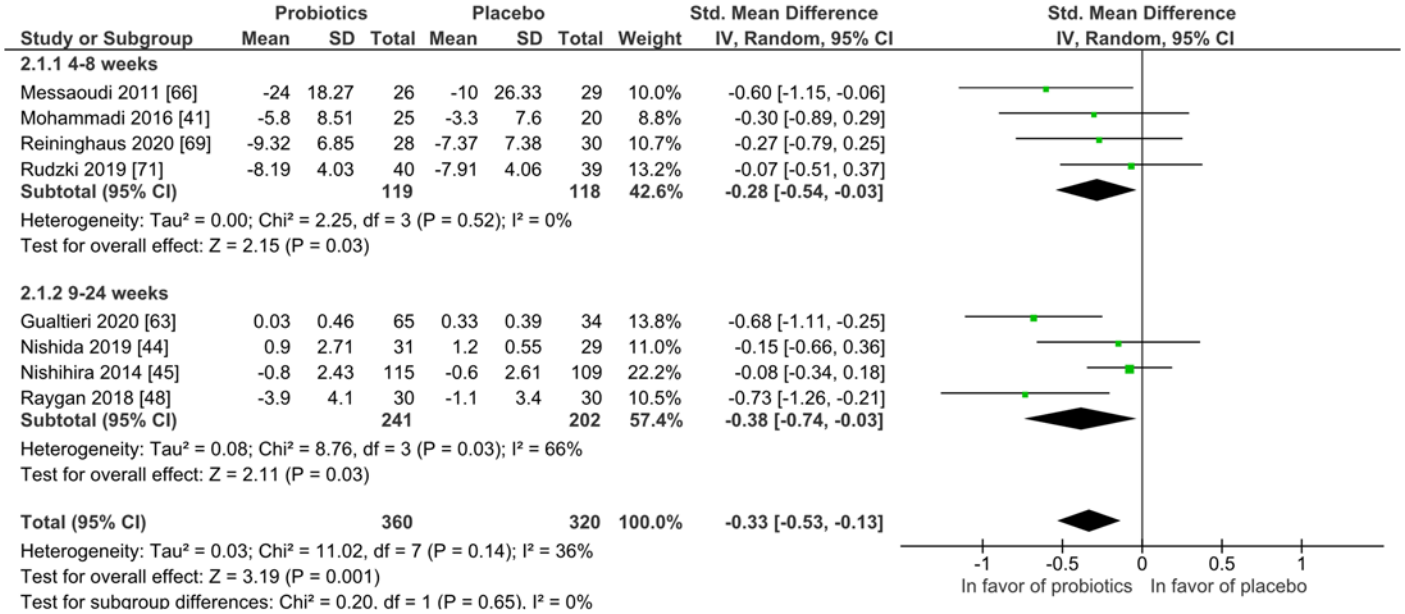

Figure S14. Effect of single-strain versus multi-strain probiotics on Psychiatric Distress in randomized controlled trials receiving either probiotics or placebo treatment. Only two studies used single-strain probiotics so that no subgroup-analysis could be conducted for this subgroup.

Study or Subgroup	Probiotics Mean	Probiotics SD	Probiotics Total	Placebo Mean	Placebo SD	Placebo Total	Weight	Std. Mean Difference IV, Random, 95% CI
**3.1.1 multi-strain**								
Gualtieri 2020 [63]	0.03	0.46	65	0.33	0.39	34	18.2%	-0.68 [-1.11, -0.25]
Messaoudi 2011 [66]	-24	18.27	26	-10	26.33	29	13.8%	-0.60 [-1.15, -0.06]
Mohammadi 2016 [41]	-5.8	8.51	25	-3.3	7.6	20	12.4%	-0.30 [-0.89, 0.29]
Nishihira 2014 [45]	-0.8	2.43	115	-0.6	2.61	109	26.6%	-0.08 [-0.34, 0.18]
Raygan 2018 [48]	-3.9	4.1	30	-1.1	3.4	30	14.4%	-0.73 [-1.26, -0.21]
Reininghaus 2020 [69]	-9.32	6.85	28	-7.37	7.38	30	14.6%	-0.27 [-0.79, 0.25]
**Subtotal (95% CI)**			**289**			**252**	**100.0%**	**-0.41 [-0.67, -0.16]**
Heterogeneity: Tau² = 0.05; Chi² = 9.42, df = 5 (P = 0.09); I² = 47%								
Test for overall effect: Z = 3.16 (P = 0.002)								
**Total (95% CI)**			**289**			**252**	**100.0%**	**-0.41 [-0.67, -0.16]**
Heterogeneity: Tau² = 0.05; Chi² = 9.42, df = 5 (P = 0.09); I² = 47%								
Test for overall effect: Z = 3.16 (P = 0.002)								
Test for subgroup differences: Not applicable								

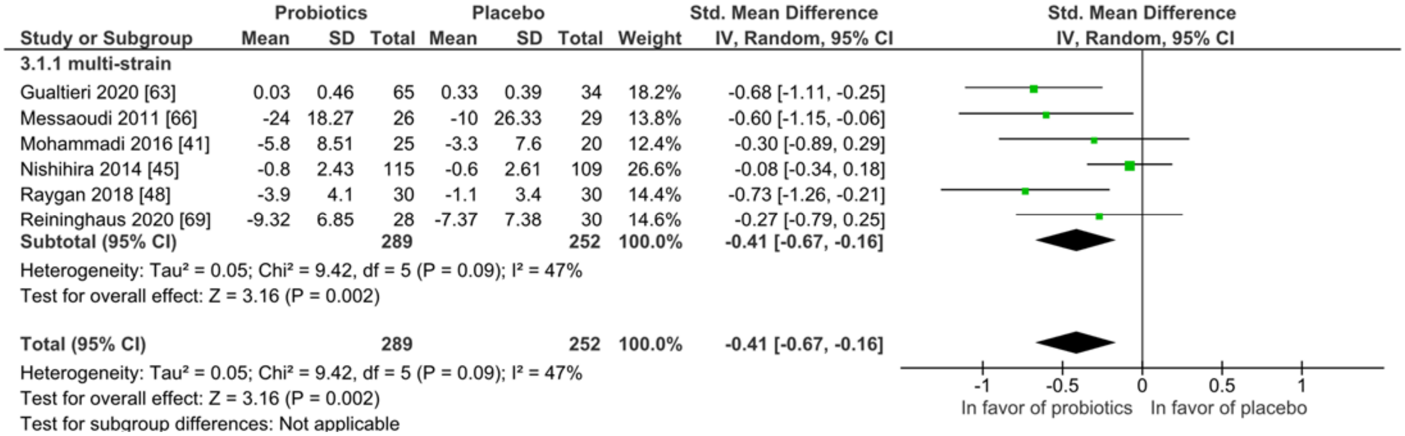

Figure S15. Effect of studies from Asia versus studies from Europe on Psychiatric Distress in randomized controlled trials receiving either probiotics or placebo treatment.

Study or Subgroup	Probiotics Mean	Probiotics SD	Probiotics Total	Placebo Mean	Placebo SD	Placebo Total	Weight	Std. Mean Difference IV, Random, 95% CI
**4.1.1 Asia**								
Mohammadi 2016 [41]	-5.8	8.51	25	-3.3	7.6	20	8.8%	-0.30 [-0.89, 0.29]
Nishida 2019 [44]	0.9	2.71	31	1.2	0.55	29	11.0%	-0.15 [-0.66, 0.36]
Nishihira 2014 [45]	-0.8	2.43	115	-0.6	2.61	109	22.2%	-0.08 [-0.34, 0.18]
Raygan 2018 [48]	-3.9	4.1	30	-1.1	3.4	30	10.5%	-0.73 [-1.26, -0.21]
**Subtotal (95% CI)**			**201**			**188**	**52.4%**	**-0.27 [-0.55, 0.02]**
Heterogeneity: Tau² = 0.03; Chi² = 4.95, df = 3 (P = 0.18); I² = 39%								
Test for overall effect: Z = 1.81 (P = 0.07)								
**4.1.2 Not Asia**								
Gualtieri 2020 [63]	0.03	0.46	65	0.33	0.39	34	13.8%	-0.68 [-1.11, -0.25]
Messaoudi 2011 [66]	-24	18.27	26	-10	26.33	29	10.0%	-0.60 [-1.15, -0.06]
Reininghaus 2020 [69]	-9.32	6.85	28	-7.37	7.38	30	10.7%	-0.27 [-0.79, 0.25]
Rudzki 2019 [71]	-8.19	4.03	40	-7.91	4.06	39	13.2%	-0.07 [-0.51, 0.37]
**Subtotal (95% CI)**			**159**			**132**	**47.6%**	**-0.40 [-0.70, -0.11]**
Heterogeneity: Tau² = 0.03; Chi² = 4.62, df = 3 (P = 0.20); I² = 35%								
Test for overall effect: Z = 2.67 (P = 0.008)								
**Total (95% CI)**			**360**			**320**	**100.0%**	**-0.33 [-0.53, -0.13]**
Heterogeneity: Tau² = 0.03; Chi² = 11.02, df = 7 (P = 0.14); I² = 36%								
Test for overall effect: Z = 3.19 (P = 0.001)								
Test for subgroup differences: Chi² = 0.43, df = 1 (P = 0.51), I² = 0%								

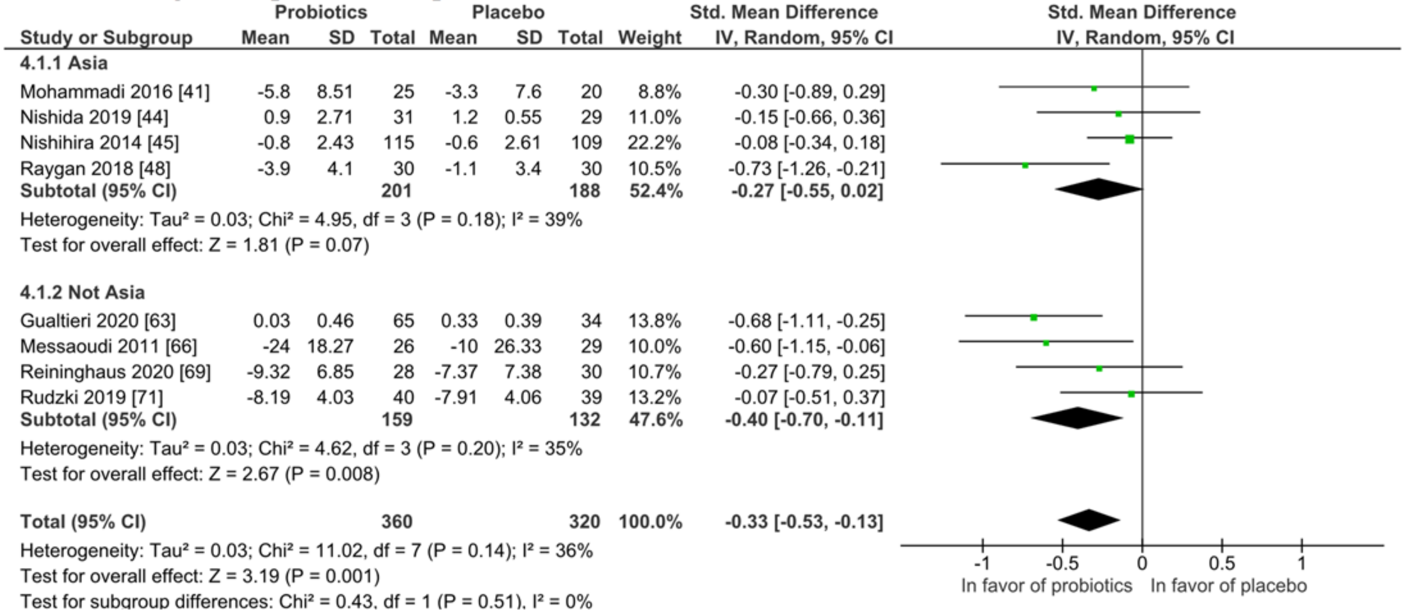

Figure S16. Effect of application form on Psychiatric Distress of randomized controlled trials receiving either probiotics or placebo treatment.

Study or Subgroup	Probiotics Mean	Probiotics SD	Probiotics Total	Placebo Mean	Placebo SD	Placebo Total	Weight	Std. Mean Difference IV, Random, 95% CI
**5.1.1 capsule/pills**								
Gualtieri 2020 [63]	0.03	0.46	65	0.33	0.39	34	13.8%	-0.68 [-1.11, -0.25]
Messaoudi 2011 [66]	-24	18.27	26	-10	26.33	29	10.0%	-0.60 [-1.15, -0.06]
Nishihira 2014 [45]	-0.8	2.43	115	-0.6	2.61	109	22.2%	-0.08 [-0.34, 0.18]
Reininghaus 2020 [69]	-9.32	6.85	28	-7.37	7.38	30	10.7%	-0.27 [-0.79, 0.25]
**Subtotal (95% CI)**			**234**			**202**	**56.6%**	**-0.37 [-0.69, -0.05]**
Heterogeneity: Tau² = 0.06; Chi² = 7.00, df = 3 (P = 0.07); I² = 57%								
Test for overall effect: Z = 2.27 (P = 0.02)								
**5.1.2 liquid/powder**								
Mohammadi 2016 [41]	-5.8	8.51	25	-3.3	7.6	20	8.8%	-0.30 [-0.89, 0.29]
Nishida 2019 [44]	0.9	2.71	31	1.2	0.55	29	11.0%	-0.15 [-0.66, 0.36]
Rudzki 2019 [71]	-8.19	4.03	40	-7.91	4.06	39	13.2%	-0.07 [-0.51, 0.37]
**Subtotal (95% CI)**			**96**			**88**	**32.9%**	**-0.15 [-0.44, 0.14]**
Heterogeneity: Tau² = 0.00; Chi² = 0.39, df = 2 (P = 0.82); I² = 0%								
Test for overall effect: Z = 1.02 (P = 0.31)								
**5.1.3 Not reported**								
Raygan 2018 [48]	-3.9	4.1	30	-1.1	3.4	30	10.5%	-0.73 [-1.26, -0.21]
**Subtotal (95% CI)**			**30**			**30**	**10.5%**	**-0.73 [-1.26, -0.21]**
Heterogeneity: Not applicable								
Test for overall effect: Z = 2.74 (P = 0.006)								
**Total (95% CI)**			**360**			**320**	**100.0%**	**-0.33 [-0.53, -0.13]**
Heterogeneity: Tau² = 0.03; Chi² = 11.02, df = 7 (P = 0.14); I² = 36%								
Test for overall effect: Z = 3.19 (P = 0.001)								
Test for subgroup differences: Chi² = 3.80, df = 2 (P = 0.15), I² = 47.4%								

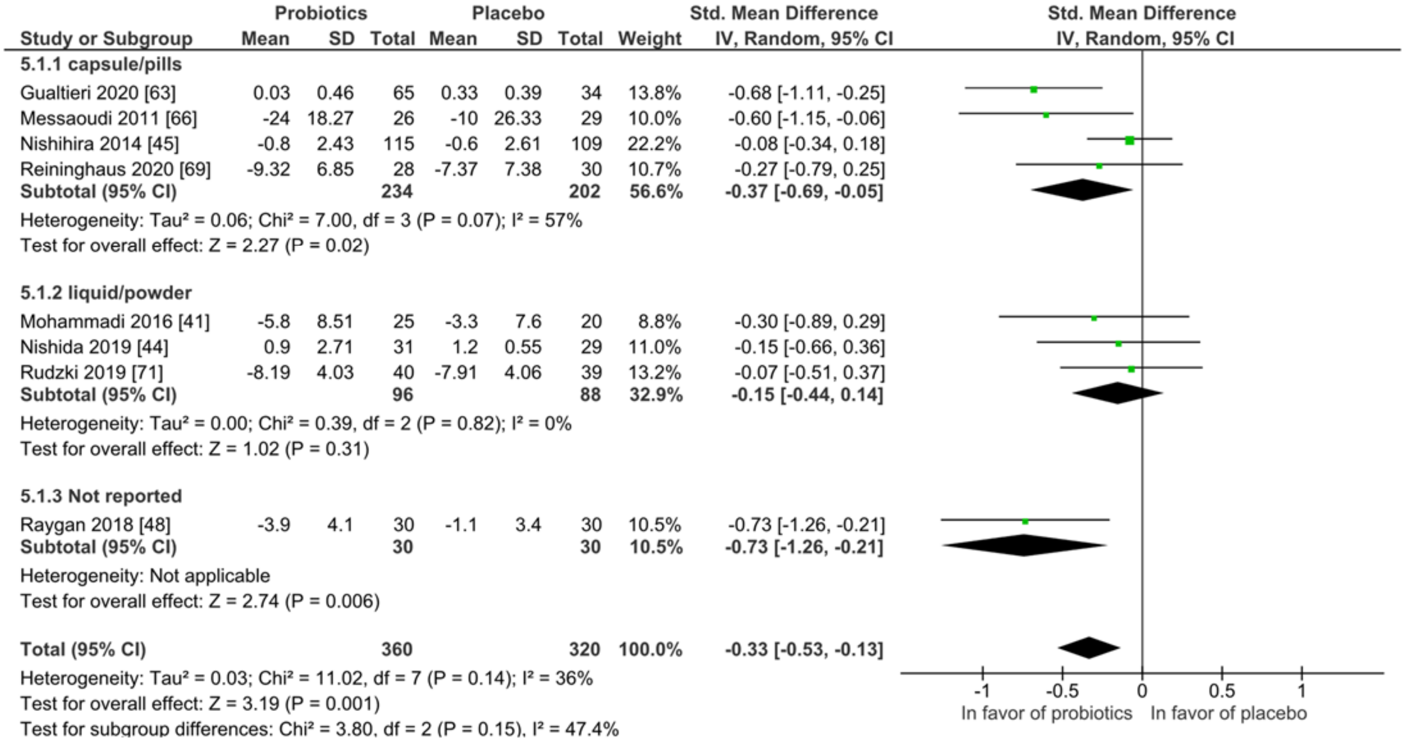

Figure S17. Effect of studies with healthy participants on Psychiatric Distress in randomized controlled trials receiving either probiotics or placebo treatment.

Study or Subgroup	Probiotics Mean	Probiotics SD	Probiotics Total	Placebo Mean	Placebo SD	Placebo Total	Weight	Std. Mean Difference IV, Random, 95% CI
Gualtieri 2020 [63]	0.03	0.46	65	0.33	0.39	34	15.9%	-0.68 [-1.11, -0.25]
Messaoudi 2011 [66]	-24	18.27	26	-10	26.33	29	11.7%	-0.60 [-1.15, -0.06]
Mohammadi 2016 [41]	-5.8	8.51	25	-3.3	7.6	20	10.3%	-0.30 [-0.89, 0.29]
Nishida 2019 [44]	0.9	2.71	31	1.2	0.55	29	12.8%	-0.15 [-0.66, 0.36]
Nishihira 2014 [45]	-0.8	2.43	115	-0.6	2.61	109	24.7%	-0.08 [-0.34, 0.18]
Raygan 2018 [48]	-3.9	4.1	30	-1.1	3.4	30	12.2%	-0.73 [-1.26, -0.21]
Reininghaus 2020 [69]	-9.32	6.85	28	-7.37	7.38	30	12.4%	-0.27 [-0.79, 0.25]
**Total (95% CI)**			**320**			**281**	**100.0%**	**-0.37 [-0.59, -0.15]**

Heterogeneity: Tau² = 0.03; Chi² = 9.91, df = 6 (P = 0.13); I² = 39%
Test for overall effect: Z = 3.26 (P = 0.001)

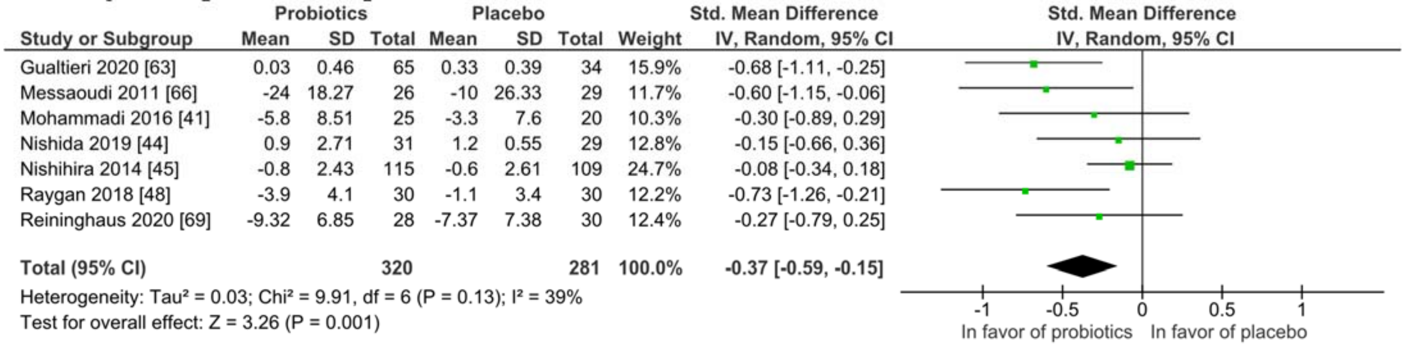

Figure S18. Overview of psychiatric distress symptom outcomes according to questionnaire. Since several studies incorporated more than one questionnaire for the same outcome, no total summary across all questionnaires is presented here.

Study or Subgroup	Probiotics Mean	Probiotics SD	Probiotics Total	Placebo Mean	Placebo SD	Placebo Total	Weight	Std. Mean Difference IV, Random, 95% CI
**1.1.1 GHQ**								
Mohammadi 2016 [41] GHQ	-5.8	8.51	25	-3.3	7.6	20	8.8%	-0.30 [-0.89, 0.29]
Nishida 2019 [44] GHQ	0.9	2.71	31	1.2	0.55	29	11.0%	-0.15 [-0.66, 0.36]
Nishihira 2014 [45] GHQ	-0.8	2.43	115	-0.6	2.61	109	22.2%	-0.08 [-0.34, 0.18]
Raygan 2018 [48] GHQ	-3.9	4.1	30	-1.1	3.4	30	10.5%	-0.73 [-1.26, -0.21]
**Subtotal (95% CI)**			**201**			**188**	**52.4%**	**-0.27 [-0.55, 0.02]**
Heterogeneity: Tau² = 0.03; Chi² = 4.95, df = 3 (P = 0.18); I² = 39%								
Test for overall effect: Z = 1.81 (P = 0.07)								
**1.1.2 SCL90**								
Gualtieri 2020 [63] SCL90	0.03	0.46	65	0.33	0.39	34	13.8%	-0.68 [-1.11, -0.25]
Messaoudi 2011 [66] SCL90	-24	18.27	26	-10	26.33	29	10.0%	-0.60 [-1.15, -0.06]
Reininghaus 2020 [69] SCL90	-9.32	6.85	28	-7.37	7.38	30	10.7%	-0.27 [-0.79, 0.25]
Rudzki 2019 [71] SCL90	-8.19	4.03	40	-7.91	4.06	39	13.2%	-0.07 [-0.51, 0.37]
**Subtotal (95% CI)**			**159**			**132**	**47.6%**	**-0.40 [-0.70, -0.11]**
Heterogeneity: Tau² = 0.03; Chi² = 4.62, df = 3 (P = 0.20); I² = 35%								
Test for overall effect: Z = 2.67 (P = 0.008)								

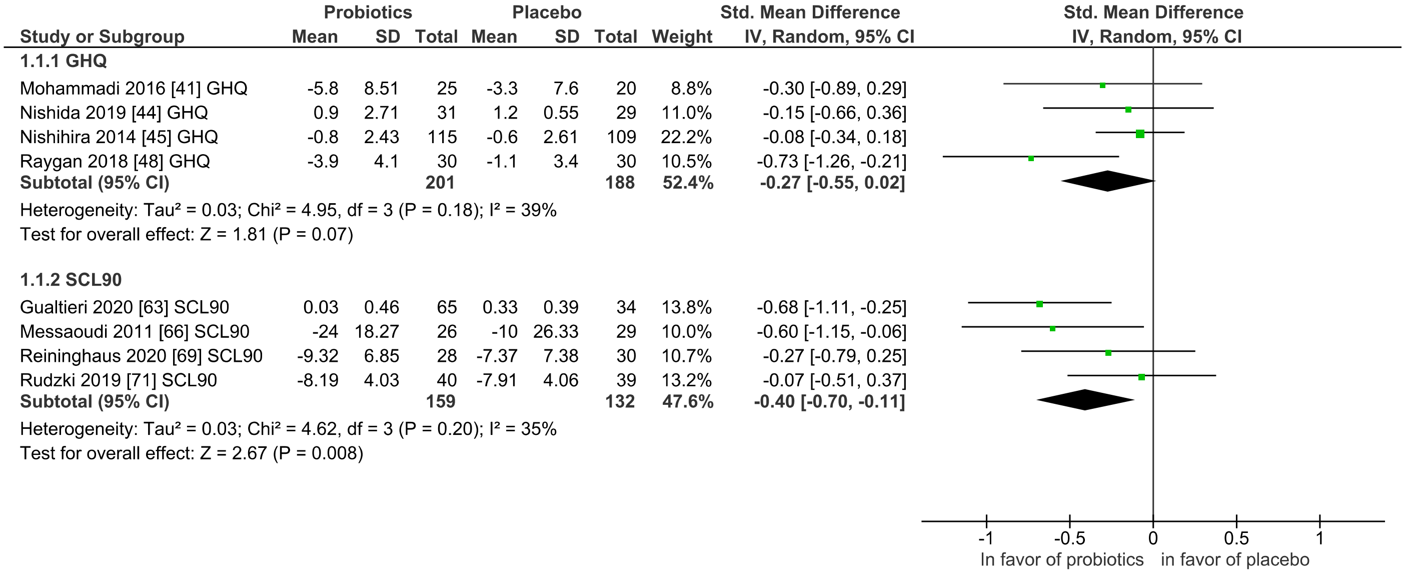

Supplement: Supplementary file 1 [file nutrients-14-00621-s001.zip › nutrients-1534290-supplementary.pdf]
